# Whole Transcriptome Profiling Identifies CD93 and Other Plasma Cell Survival Factor Genes Associated with Measles-Specific Antibody Response after Vaccination

**DOI:** 10.1371/journal.pone.0160970

**Published:** 2016-08-16

**Authors:** Iana H. Haralambieva, Michael T. Zimmermann, Inna G. Ovsyannikova, Diane E. Grill, Ann L. Oberg, Richard B. Kennedy, Gregory A. Poland

**Affiliations:** 1 Mayo Clinic Vaccine Research Group—Department of Medicine, Mayo Clinic and Foundation, Rochester, MN, United States of America; 2 Division of Biomedical Statistics and Informatics- Department of Health Science Research, Mayo Clinic and Foundation, Rochester, MN, United States of America; Imperial College London, UNITED KINGDOM

## Abstract

**Background:**

There are insufficient system-wide transcriptomic (or other) data that help explain the observed inter-individual variability in antibody titers after measles vaccination in otherwise healthy individuals.

**Methods:**

We performed a transcriptome(mRNA-Seq)-profiling study after *in vitro* viral stimulation of PBMCs from 30 measles vaccine recipients, selected from a cohort of 764 schoolchildren, based on the highest and lowest antibody titers. We used regression and network biology modeling to define markers associated with neutralizing antibody response.

**Results:**

We identified 39 differentially expressed genes that demonstrate significant differences between the high and low antibody responder groups (p-value≤0.0002, q-value≤0.092), including the top gene *CD93* (p<1.0E^-13^, q<1.0E^-09^), encoding a receptor required for antigen-driven B-cell differentiation, maintenance of immunoglobulin production and preservation of plasma cells in the bone marrow. Network biology modeling highlighted plasma cell survival (*CD93*, *IL6*, *CXCL12*), chemokine/cytokine activity and cell-cell communication/adhesion/migration as biological processes associated with the observed differential response in the two responder groups.

**Conclusion:**

We identified genes and pathways that explain in part, and are associated with, neutralizing antibody titers after measles vaccination. This new knowledge could assist in the identification of biomarkers and predictive signatures of protective immunity that may be useful in the design of new vaccine candidates and in clinical studies.

## Introduction

Despite the widespread use of measles vaccines (especially the two-dose immunization schedule introduced in 1989) and the dramatic decrease in the occurrence of measles in the U.S., annual outbreaks involving hundreds of cases continue to occur.[1] In 2014 alone, the U.S. experienced 23 measles outbreaks with 644 cases (the highest number of cases since measles elimination was declared a priority in 2000).[2] In 2015, 189 people from 24 states and the District of Columbia were reported to have measles.[2] Recent outbreaks among highly vaccinated populations in North America and in Europe are an indication that, even with the two-dose schedule, vaccine failure still accounts for a small to sizeable percentage (1%-24%) of measles cases.[3,4] A significant portion of these cases involve recipients of two doses of measles-containing vaccine.[1,3]

There are insufficient data on the immunologic, genetic and genomic basis of inter-individual immune response variations after measles vaccination to help uncover the molecular mechanisms underlying vaccine failure. Comprehensive omics-level information on measles vaccine immune responses and waning immunity has not been previously generated. To fill these knowledge gaps, we developed a new vaccinomics paradigm that utilizes unbiased, high-dimensional omics data and advanced statistical/bioinformatics approaches for deep immune profiling of viral vaccine responses.[5,6,7] The ultimate goal is to apply these approaches to identify signatures (containing both individual variables and their interactions) that can discriminate among measles vaccine immune phenotypes, serve as biomarkers of protective immunity, and inform the development of novel vaccine candidates.

In this study, we assessed gene expression in 30 measles-mumps-rubella (MMR) vaccine recipients following two vaccine doses using a whole transcriptome sequencing approach (mRNA-Seq), and evaluated how high or low neutralizing antibody response impacts and/or is associated with observed transcriptional changes after *in vitro* measles virus stimulation. Our study was designed to better understand the genetic factors, mechanisms and pathways underlying the biological spectrum of immune responses to measles vaccination.

## Methods

The methods described herein are similar or identical to those we have previously published. [8,9,10,11,12,13,14]

### 2.1. Study subjects

A total of 30 subjects were selected for an mRNA-Seq transcriptome-profiling study based on their plaque reduction microneutralization assay (PRMN) antibody titers (15 highest and 15 lowest antibody responders) from a combined cohort of 764 randomly selected healthy schoolchildren and young adults (age 11–22 years) from Olmsted County, MN, who received two doses of MMR-II vaccine (Merck) that contained the Edmonston strain of measles virus (TCID_50_ ≥1,000). Details on the recruitment, demographic and immune characteristics of the study population have been previously described.[10,11,12] The Institutional Review Board (IRB) of the Mayo Clinic approved the study, and written, informed consent was obtained from subjects’ parents/guardians, as well as written assent from age-appropriate subjects at the time of enrollment.

### 2.2. Plaque reduction microneutralization assay (PRMN)

Anti-measles neutralizing antibody titers were quantified using a high throughput fluorescence-based PRMN, as previously published.[10,11,12] Estimates of 50% end point titer (Neutralizing Dose, ND50) were calculated using Karber’s formula and ND50 values were converted to mIU/ml values using the 3rd WHO anti-measles antibody standard (NIBSC code no. 97/648), as previously published. [12] The coefficient of variation for this assay in our laboratory, based on the third WHO standard runs was 5.7%.[12]

### 2.3. mRNA-Seq transcriptome profiling

The sequencing methods are similar to those previously published.[13,14] In brief, subjects’ cryopreserved peripheral blood mononuclear cells (PBMCs) were thawed and stimulated with live Edmonston measles virus at a multiplicity of infection of 0.5 for 24 hours (for each subject, an aliquot of the cells was left unstimulated). Cells were stabilized with RNAprotect cell reagent (Qiagen) and total RNA was extracted using RNeasy Plus mini kit (Qiagen). Quality and quantity of RNA was determined by Nanodrop spectrophotometry (Thermo Fisher Scientific). Poly-A RNA was isolated using magnetic beads coated with olido-dT, then reverse transcribed after fragmentation into cDNA, and combined with Illumina adaptor sequences. Libraries were generated using Illumina’s mRNA TruSeq (v1) kit. After validation, cDNA libraries were sequenced (paired end sequencing) on an Illumina HiSeq 2000 (Illumina;San Diego, CA) with Illumina’s TruSeq Cluster kit (v3-cBot-HS) and 51 Cycle Illumina TruSeq SBS Sequencing Kit (v3). The sequencing reads were aligned to the human genome build 37.1 using TopHat (1.3.3) and Bowtie (0.12.7). Gene counts were performed using HTSeq (0.5.3p3), while BEDTools software (2.7.1) was used to map normalized read count to individual exons.[15,16,17]

### 2.4. Statistical analysis

The analysis consisted of complementary gene-to-biology (an inductive, data-driven approach performed at the gene level) and biology-to-gene (a deductive knowledge-driven approach performed at the geneset level) analytical strategies, as previously described.[18]

The samples (n = 60, 30 measles virus-stimulated and 30 unstimulated samples) were randomized to ensure balance of important characteristics over assay run order: immune response group (high or low antibody responder), stimulation status (virus stimulated or unstimulated sample), and sex (male or female) for the cell culture setup, preparation of libraries, and sequencing (flow cell and lane run).

Quality control methods used were similar to those reported in our previous studies. [13,14] Conditional Quantile Normalization (CQN) was used to normalize the mRNA-sequence data adjusting for the differences in library size, GC content and gene length.[19,20] Differential expression analysis comparing statistical “interaction” of the high vs low responders’ response to viral stimulation; viral stimulation relative to the unstimulated samples (regardless of neutralizing antibody status) was performed using generalized estimating equations.[21] Specifically, the raw counts were modeled in a generalized linear negative binomial model [22], utilizing the offset from the CQN normalization, estimating the variance for each gene with the edgeR tagwise dispersion option, and estimating the within subject correlation structure to provide a robust estimate of the variance.[23] Results for the differential expression analysis are reported as the interaction comparing the fold change (FC) for the difference in high antibody responders’ response to viral stimulation, relative to the response of low antibody responders’ to viral stimulation, and the response to viral stimulation for the high and low responder subjects combined. All reported fold-change values (including the interaction fold change) for gene expression are for virus-stimulated PBMCs relative to the unstimulated PBMCs, and thus take into account resting differences in gene expression. False discovery rates (FDR) were calculated using standard methods and are reported as q-values.[24] Pathway analysis was conducted using Ingenuity® software, (Ingenuity® Systems; Redwood City, CA). Geneset analysis was done using the gamma method with 1,000 permutations of the high and low antibody response of the subjects permuting the stimulated status within each subject.[25]

### 2.5. Network biology

To comprehensively identify high-confidence interactions, we combined multiple resources: HPRD [26]; CCSB [27]; the 7.8% of STRING [28] with highest confidence (score ≥ 70%); and the 12.9% of HumanNet [29] with highest confidence (score ≥ 2). Networks were visualized using Cytoscape [30] version 3.2.1 and layouts refined using AllegroLayout v.2.2.1.[31] Pathway enrichment was performed using 138 pathway definitions downloaded from MSigDB’s index of KEGG canonical pathways [32,33] after removing those that are disease-associated (e.g., type-II diabetes and leishmania infection), or derived from other pathways (e.g., pathways in cancer). The biologic functions of genesets were assessed using GO term enrichment [34] and evaluated using hypergeometric tests. Genes considered in our network biology approach are all those exhibiting stimulation and interaction fold changes (|log_2_(FC)| of ≥ 0.5)and an interaction p-value ≤ 0.01 (n = 115), or a stimulation p-value of ≤ 0.001 (n = 207 additional genes). One-hundred forty (43%) of these 322 genes share connections within the high-confidence network used. We also generated an inclusive network where the score-based filters were not employed, and where 282 (88%) genes share connections (data not shown).

## Results

### 3.1. Characteristics of the study subjects

The enrolled study subjects were primarily white (67% for the high-responders group and 73% for the low-responders group); African-Americans comprised 27% and 20% of the high- and low-responders group, respectively. Sixty percent of the participants were males and forty percent were females. The median age of subjects at study enrollment was 16 (IQR 12, 17) and 15 (IQR 13, 17) years (for the high and low responders group, respectively); the median age at first measles immunization was 16 (IQR 15, 32) and 15 (IQR 15, 65) months; the median age at second immunization was 6 (IQR 4, 12) and 8 (IQR 5, 11) years; and the median time since second immunization to enrollment was 7.1 (5.7, 9.5) and 7.0 (IQR 4.6, 8.7) years (for the high and low responders group, respectively). Thus, it is important to note that gene expression in PBMCs and all immune measures (neutralizing antibody and cytokine measures after *in vitro* viral stimulation) were quantified in samples approximately seven years after the last/second measles (MMR) vaccination. None of the above mentioned demographic variable differences reached statistical significance (data not shown). The study subjects were selected for mRNA-Seq profiling based on their neutralizing antibody titer. The median antibody titer for the high responders group was 5,188 mIU/mL (which suggests protection against infection/disease), while the titer for the low responders group was 88 mIU/mL (which suggests lack of protection against measles). All other immune response variables were not statistically significant between the two groups and are shown in S1 Table.

### 3.2. Gene expression after in vitro stimulation with measles virus in high and low antibody responders (interaction analysis)

Our overall analysis (differential gene expression in response to viral stimulation in all samples/subjects) identified 1,761 significantly expressed genes (FC>2 or FC<0.5, FDR<0.004), including chemokine and chemokine receptor genes, cytokine and cytokine receptor genes, and genes encoding innate receptors, antiviral proteins and HLA (S2 Table).

Importantly, we identified 39 differentially expressed genes (FDR<0.1, Table 1) that demonstrated significant gene expression differences between the two antibody responder groups to measles vaccination (p-value range 1.0E^-13^ to 0.0002, q-values range 1.0E^-09^ to 0.092, Table 1). An additional 11 differentially expressed genes demonstrated suggestive gene expression differences (as per our gene-to-biology approach) between the two study groups (FDR<0.15, Table 1).

**Table 1:** Differential response to measles virus stimulation in high vs. low antibody vaccine responders (genes with q-value <0.15).

Gene symbol	FC low^a^	FC low log2^b^	FC high^c^	FC high log2^d^	FC interaction^e^	FC int. log2^f^	p-value^g^	q-value
*CD93*	0.06	-3.99	0.41	-1.28	6.55	2.71	<1.0E^-13^	<1.0E^-09^
*IL24*	0.75	-0.42	1.52	0.6	2.02	1.02	5.80E^-13^	4.55E^-09^
*PID1*	0.01	-6.5	0.14	-2.83	12.78	3.68	3.34E^-11^	1.75E^-07^
*CCL20*	1.4	0.48	0.73	-0.45	0.52	-0.93	1.85E^-09^	7.24E^-06^
*ITGB8*	3.73	1.9	1.71	0.77	0.46	-1.13	3.78E^-08^	0.0001
*EHD2*	0.96	-0.05	0.61	-0.72	0.63	-0.67	5.96E^-07^	0.0016
*IL6*	3.1	1.63	1.53	0.61	0.49	-1.02	8.77E^-07^	0.0017
*GPR124*	0.26	-1.93	0.7	-0.51	2.68	1.42	9.17E^-07^	0.0017
*S1PR3*	1.61	0.68	1.03	0.04	0.64	-0.64	9.69E^-07^	0.0017
*TFPI2*	1.94	0.95	0.77	-0.37	0.4	-1.33	1.89E^-06^	0.0027
*C9orf6*	0.76	-0.4	0.85	-0.24	1.12	0.17	1.91E^-06^	0.0027
*RFX8*	3.26	1.7	0.75	-0.42	0.23	-2.13	3.80E^-06^	0.005
*VNN1*	0.04	-4.56	0.34	-1.54	8.09	3.02	6.01E^-06^	0.007
*CYP3A5*	0.88	-0.19	0.49	-1.03	0.56	-0.85	6.41E^-06^	0.007
*BCL2A1*	2.12	1.08	1.04	0.06	0.49	-1.02	8.04E^-06^	0.008
*SLC35B3*	1.02	0.03	0.96	-0.06	0.93	-0.1	9.33E^-06^	0.009
*SMPDL3A*	1.27	0.34	0.51	-0.98	0.4	-1.32	1.25E^-05^	0.012
*NAMPT*	2.3	1.2	1.04	0.05	0.45	-1.15	1.90E^-05^	0.017
*CHST7*	3.42	1.78	2.08	1.05	0.61	-0.72	2.35E^-05^	0.019
*LIF*	5.23	2.39	1.37	0.45	0.26	-1.93	3.41E^-05^	0.027
*FLT1*	1.03	0.04	0.64	-0.65	0.62	-0.69	3.73E^-05^	0.028
*HTR7*	0.14	-2.88	0.36	-1.48	2.64	1.4	4.09E^-05^	0.029
*VEGFA*	0.82	-0.29	0.46	-1.12	0.56	-0.83	4.91E^-05^	0.033
*MAP1LC3A*	1.99	0.99	1.12	0.16	0.56	-0.83	5.52E^-05^	0.036
*FAM149B1*	1.11	0.15	1.03	0.05	0.93	-0.1	6.13E^-05^	0.038
*AK4*	6.65	2.73	1.98	0.98	0.3	-1.75	6.24E^-05^	0.038
*FPR2*	1.96	0.97	0.37	-1.43	0.19	-2.4	8.03E^-05^	0.047
*SIGLEC15*	0.14	-2.87	0.83	-0.27	6.09	2.61	9.11E^-05^	0.051
*MSC*	8.36	3.06	3.18	1.67	0.38	-1.39	0.0001	0.054
*HES1*	1.34	0.42	0.82	-0.29	0.61	-0.72	0.0001	0.054
*SGCD*	0.33	-1.62	0.46	-1.12	1.42	0.5	0.0001	0.06
*CYP1A1*	4.6	2.2	1.65	0.72	0.36	-1.48	0.0001	0.066
*MET*	2.41	1.27	1	0	0.42	-1.27	0.0001	0.066
*IL36RN*	78.19	6.29	12.19	3.61	0.16	-2.68	0.0001	0.066
*LOC154092*	1.13	0.18	0.52	-0.95	0.46	-1.13	0.0001	0.066
*G0S2*	0.29	-1.77	1.01	0.01	3.44	1.78	0.0002	0.078
*OR52N4*	2.56	1.36	1.71	0.77	0.67	-0.58	0.0002	0.081
*TRPA1*	2.22	1.15	1.14	0.19	0.52	-0.96	0.0002	0.086
*LAMB3*	0.28	-1.81	0.68	-0.56	2.38	1.25	0.0002	0.092
*ST20*	0.92	-0.11	0.71	-0.49	0.77	-0.38	0.0003	0.101
*STK31*	0.95	-0.08	0.71	-0.49	0.75	-0.42	0.0003	0.101
*CCL24*	0.02	-5.70	0.07	-3.93	3.40	1.76	0.0003	0.109
*CD33*	0.07	-3.93	0.13	-2.97	1.95	0.96	0.0003	0.109
*IL10*	0.61	-0.72	1.69	0.76	2.78	1.48	0.0003	0.110
*LZTS1*	1.12	0.17	0.86	-0.22	0.76	-0.39	0.0003	0.113
*ITGB3*	1.60	0.68	1.07	0.10	0.67	-0.57	0.0004	0.120
*DLEU2*	1.74	0.80	1.44	0.52	0.82	-0.28	0.0004	0.134
*STX1A*	0.79	-0.34	0.52	-0.93	0.66	-0.59	0.0004	0.135
*LOC730227*	1.11	0.15	0.77	-0.37	0.70	-0.52	0.0005	0.146
*CXCL2*	0.61	-0.71	1.24	0.31	2.04	1.03	0.0005	0.146

^a^Fold change gene expression for the virus-stimulated PBMCs (low responders) relative to the unstimulated PBMCs (low responders}

^b^Log_2_ of the fold change, described in (a).

^c^Fold change gene expression for the virus-stimulated PBMCs (high responders) relative to the unstimulated PBMCs (high responders}.

^d^Log_2_ of the fold change, described in (c).

^e^Interaction fold change is the ratio of the fold change for the stimulated (high responders) relative to unstimulated (high responders) (c) relative to the stimulated (low responders) relative to the unstimulated (low responders) (a)

^f^Log_2_ fold change for the interaction (e).

^g^P-value associated with the test for a gene expression difference in high responders (response to viral stimulation), relative the response of low responders (response to viral stimulation). For *CADM1*, p-value = 0.0008 and q-value = 0.203

### 3.3. Pathway and geneset analysis

Among the Ingenuity pathways that were significantly enriched in high vs. low antibody responders (enrichment p<1.0E^-03^), the top three pathways (consisting of highly overlapping genes) are related to immune adhesion and function, chemotaxis/inflammatory response, and cytokine regulation (Table 2). To augment Ingenuity-based analysis, we used other independent annotation sources, including GO and a filtered set of canonical pathways (see Methods). The top results from each comparison are listed in Table 3, with most sources highlighting chemokine/cytokine activity, inflammatory response, and cell-cell communication/adhesion as biological processes significantly involved in differential response to viral stimulation in high vs. low measles vaccine antibody responders.

**Table 2:** Top Ingenuity enriched pathways of differentially expressed genes to measles virus stimulation in high vs. low antibody vaccine responders.

Top Ingenuity canonical pathways	Pathway enrichment p-value	Genes	Individual gene expression p-values
1. Granulocyte Adhesion and Diapedesis	1.34E^-07^	*CCL2*, *CCL3*, *CCL16*, *CCL18*, *CCL20*, *CCL24*, *CLDN11*, *CLDN12*, *CLDN14*, *CLDN23*, *CXCL2*, *CXCL3*, *CXCL5*, *CXCL6*, *CXCL13*, *FPR2*, *GNAI1*, *IL1B*, *IL1R1*, *IL36RN*, *ITGB3*, *MMP14*, *MMP19*, *PPBP*, *SDC4*	4.97E^-02^ to 1.85E^-09^
2. Lymphocyte and Monocyte Adhesion and Diapedesis	5.76E^-06^	*CCL2*, *CCL3*, *CCL16*, *CCL18*, *CCL20*, *CCL24*, *CLDN11*, *CLDN12*, *CLDN14*, *CLDN23*, *CXCL2*, *CXCL3*, *CXCL5*, *CXCL6*, *CXCL13*, *GNAI1*, *IL1B*, *IL1R1*, *IL36RN*, *MMP14*, *MMP19*, *PPBP*, *SDC4*	4.97E^-02^ to 1.85E^-09^
3. Differential Regulation of Cytokine Production in Macrophages and T Helper Cells by IL-17	7.41E^-04^	*CCL2*, *CCL3*, *CCL16*, *CCL18*, *CCL20*, *CCL24*, *CLDN11*, *CLDN12*, *CLDN14*, *CLDN23*, *CXCL2*, *CXCL3*, *CXCL5*, *CXCL6*, *CXCL13*, *GNAI1*, *IL1B*, *IL1R1*, *IL36RN*, *MMP14*, *MMP19*, *PPBP*, *SDC4*	4.97E^-02^ to 1.85E^-09^

**Table 3:** Pathway enrichment of differentially expressed genes upon measles virus stimulation using independent annotation sources.

			*Effect of Viral Stimulation (in all subjects)*			*Interaction (high vs*. *low Ab responders)*		
Source^a^	Term	M^b^	N^c^	p-value	q-value	N^c^	p-value	q-value
*GOA*	Extracellular Space	685	49	1.9E^-15^	2.9E^-11^	23	7.1E^-10^	6.0E^-06^
*GOA*	Cytokine Activity	92	12	2.1E^-07^	2.2E^-04^	8	3.6E^-07^	1.9E^-03^
*GOA*	Chemokine Mediated Signaling	51	15	2.5E^-14^	2.0E^-10^	6	1.8E^-06^	4.9E^-03^
*GOA*	Inflammatory Response	263	27	1.5E^-12^	4.1E^-09^	12	5.0E^-07^	2.0E^-03^
*MSigDB*	NABA Matrisome	539	58	3.8E^-27^	1.7E^-23^	28	1.3E^-16^	1.2E^-12^
*MSigDB*	Hallmark of Inflammatory Response	181	29	1.3E^-18^	2.9E^-15^	16	2.7E^-13^	6.6E^-10^
*KEGG CP*	Cytokine Cytokine-Receptor Interaction	187	28	3.3E^-17^	4.6E^-15^	10	1.1E^-06^	1.5E^-04^
*KEGG CP*	Chemokine Signaling	164	16	1.3E^-07^	8.6E^-06^	5	7.1E^-03^	2.4E^-01^
*KEGG CP*	Focal Adhesion	161	9	4.2E^-03^	8.3E^-02^	6	1.2E^-03^	8.1E^-02^

^a^The annotation source: GOA, human Gene Ontology Annotation; MSigDB genesets, excluding those derived from GO terms, genomic proximity, and cancer; KEGG CP, our filtered subset of canonical pathways (see Methods).

^b^The total number of genes that are annotated with the given term.

^c^The number of genes from our analysis and passing statistical significance thresholds (see Methods) that are annotated with the given term.

We also performed a geneset analysis using genesets/modules downloaded from MSigDB to identify associations of groups of genes (genesets) with neutralizing antibody response following vaccination. [35] The results identified 112 significant genesets (p < 0.05) with different expression (response to viral stimulation) in high vs. low antibody responders that comprise genes/pathways integral to innate and adaptive immune response, cell adhesion, metabolism and cell signaling. The top 7 significant genesets with p<0.003 are listed in S3 Table.

In search of consistency between our gene-to-biology and biology-to-gene approach results, we compared the top 69 genes from the gene-to-biology results (with an interaction p-value < = 0.001) with the genes from 1,213 genesets (with p-value ≤ 0.002) resulting from our biology-to-gene approach. The intersection (overlap) between the results from the two approaches resulted in six common genes of high interest (i.e., *CD93*, *CD33*, *IL10*, *VEGFA*, *VNN1*, *CADM1*).

### 3.4. Network biology

**Fig 1:**
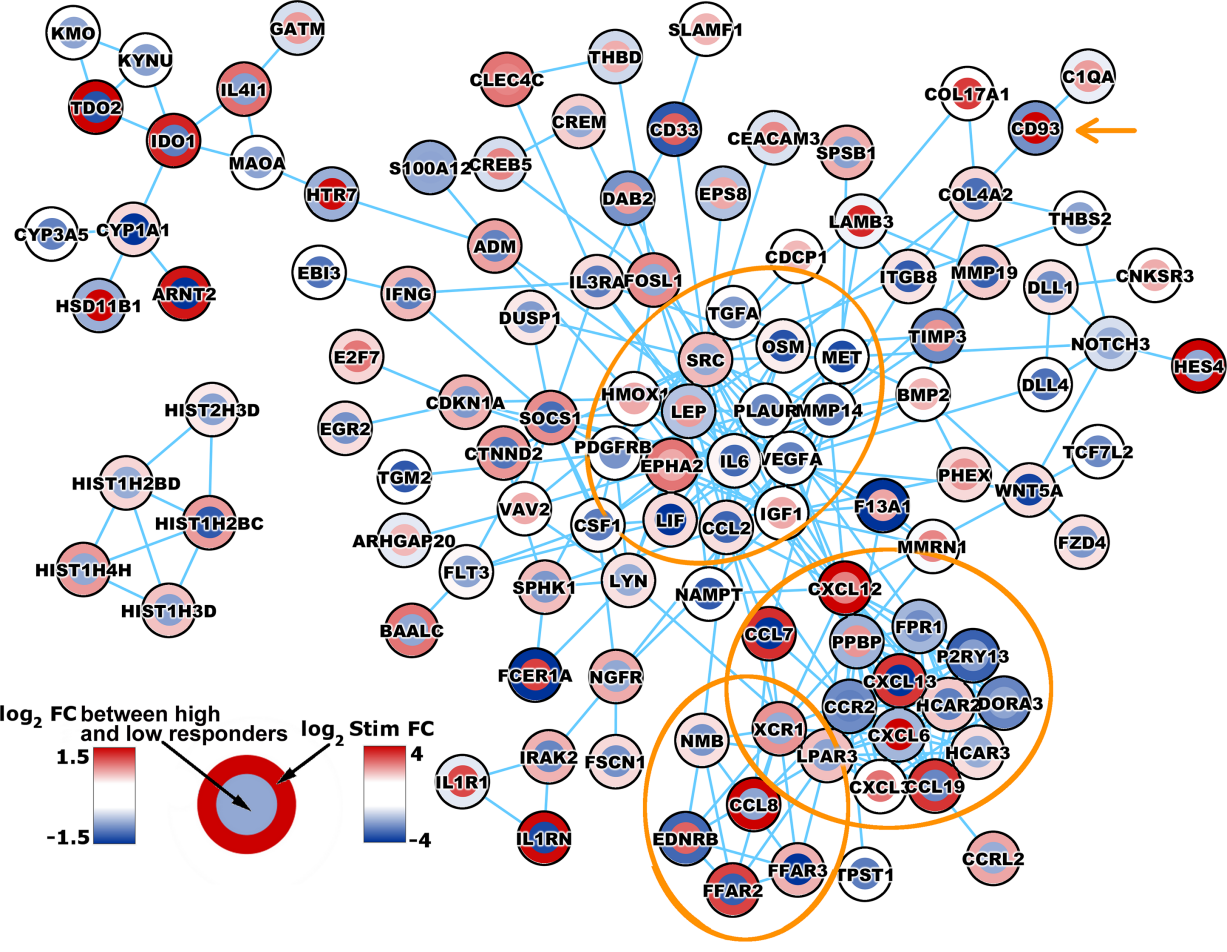
Legend. Network biology of gene expression in measles vaccine high and low antibody responders.Simultaneous visualization of overall stimulation response (gene expression in response to viral stimulation in all subjects) and differential responses (in high vs. low antibody responders) within the context of a high-confidence biologic network. Each gene is represented as two circles, the outer colored by the virus-induced fold change in all subjects (FC, presented as log_2_ Stim FC) and the inner by the statistical interaction between high and low responders (i.e., difference in stimulated FC between the high and low antibody responder groups, presented as log_2_ FC between high and low responders).

To display molecular interactions, we performed simultaneous gene mapping/visualization of: 1.) genes with significant induction/suppression upon viral stimulation (overall response to viral stimulation in all study subjects, regardless of their antibody response); and 2.) genes that show significant differences in gene expression between the two antibody responder groups in response to viral stimulation (i.e., statistical interaction). The result of gene mapping using only high-confidence molecular interactions is shown in Fig 1.

## Discussion

We and others have pointed out the value of using a systems-level vaccinomics approach leveraging unbiased large-scale gene expression profiling (e.g., mRNA-Seq) to delineate molecular signatures of efficient immune response after vaccination. Such approaches have provided important insights into immunity to influenza [36], yellow fever [37], smallpox [14], rubella [13], polysaccharide pneumococcal [38], and other vaccines. Our motivation for performing this study was that despite the continuing measles outbreaks in populations that have received two doses of measles vaccine, comprehensive genome-wide information on gene expression changes after measles vaccination and/or infection is limited.

Our transcriptome profiling study delineates significant gene expression differences that discriminate between high and low neutralizing antibody responders (to measles vaccination) in PBMCs stimulated *in vitro* with measles virus. The identified genes include cytokine genes, cell signaling genes, transcriptional targets of NF-kappa B, C-type lectin transmembrane receptors, and other immune function-related genes (Table 1). The top gene found (p-value<1.0E^-13^, Table 1) is *CD93*, which is a receptor required for antigen-driven B-cell differentiation for maintenance of immunoglobulin production and preservation of plasma cells in the bone marrow. [39] *CD93* was not only the most significant gene, but it was also among the top three genes with the highest fold change (FC interaction = 6.55) for differential gene expression (upon viral stimulation) between high and low antibody responders. The other two genes with high fold change were the phosphotyrosine interaction domain containing 1 gene (*PID1*, FC interaction = 12.78, p-value = 3.34E^-11^) and vanin 1(*VNN1*, FC interaction = 8.09, p-value = 6.01E^-06^). *CD93* (identified also as a complement protein 1q receptor—C1qRp) encodes a C-type lectin transmembrane receptor, expressed primarily during the early stages of B cell differentiation (although can be expressed by different cell lineages, including activated macrophages). CD93 expression is re-induced during plasma cell differentiation and the long-lived plasma cells demonstrate high expression level of this protein, which is crucial for their maintenance in the bone marrow niche.[39,40] Studies in a CD93-deficient mouse model revealed the key role CD93 has in terms of the survival of antigen-specific long-lived plasma cells and the persistence of antigen-specific antibody response.[39]

Interestingly, the expression of CD93 was downregulated upon measles virus stimulation in our study (in both antibody responder groups); however, this downregulation should be interpreted only in the context of gene expression in PBMCs (and not in specific cell populations). A study of gammaherpesvirus infection in mice has demonstrated that the majority of normal B cells were activated in the course infection with downregulation of CD93. [41] Different B cells may undergo different degrees of CD93 expression (including loss of CD93); however, the degree of downregulation or upregulation in different individuals and cell subtypes may underlie differences in cell homing, adhesion and cell survival. The downregulation (FC log2) in the high antibody responder group was limited compared to the low-responder group (i.e., CD93 expression remained at a higher expression level after viral stimulation in the high antibody responders compared to the low antibody responders), which is in agreement with the association of higher CD93 expression with long-term antibody persistence after vaccination. Furthermore, CD93 cytoplasmic domain has been shown to interact with moesin for cytoskeleton remodeling, and thus this protein may have a role beyond maintenance of long-lived plasma cells in the processes of cell adhesion, cell migration and phagocytosis.[42] Another interesting finding is the identification of *IL24* as the second-ranked top gene (p = 5.8E^-13^, Table 1), demonstrating transcriptional differences in high vs. low neutralizing antibody responders. IL-24 is a novel cytokine with a recently described role in regulating antigen-driven driven B cell differentiation and plasma cell vs. memory cell commitment in germinal centers; therefore, its expression can be directly related to antibody production.[43]

Different annotation sources emphasize several major enrichment themes among the genes passing statistical criteria: cytokine/chemokine activity/extracellular messengers, inflammatory response and cell adhesion/chemotaxis/migration. Specifically, Gene Ontology (GO) annotates many genes as cytokine and chemokine signaling localized in the extracellular space. MSigDB genesets highlight the importance of inflammatory response and the matrisome (the “ome” of extracellular matrix proteins). The filtered set of KEGG canonical pathways likewise highlights both cytokine and chemokine signaling pathways and cell adhesion/migration processes, which is similar to Ingenuity pathways results.

Our network biology analysis mapped genes with significant statistical associations with either overall (in all study subjects) virus-induced gene induction/suppression, or significant genes that discriminate the transcriptional patterns between high and low antibody responder groups to reveal their molecular interaction and biological relationships (Fig 1). Several modules are evident: one predominant module is centered on *IL6*, *VEGFA*, *LEP*, and the two tyrosine-protein kinases *SRC* and *EPHA2*, and includes genes with a distinct known relation to innate/adaptive immunity. Interleukin 6 is produced by various cell types, including macrophages and T cells to initiate and promote immune function, and is also a critical survival factor for the resident bone-marrow plasma cells.[44] Leptin is a known protein important for regulation of metabolism and body weight, which plays an important role in the modulation of inflammatory/innate and adaptive immune responses following infection or vaccination.[45] A second predominant module is linked to chemotaxis/inflammation and is centered on *CCR2* (encodes a receptor for monocyte chemoattractant protein-1, involved in monocyte chemotaxis/infiltration in inflammation) and several other key chemoattractants (or their receptors) for B and T cells, monocytes, dendritic cells and granulocytes (i.e., *CXCL3*, *CXCL6*, *CXCL12*, *CXCL13*, *CCL7*, *CCL19*, *XCR1)*. A third smaller module is centered on *CCL8*, which is also a chemotactic protein with a role in inflammatory response and migration/activation of monocytes, T cells and NK cells. Previous studies have identified similar or identical genes (e.g., *IL-6*, *CXCL3*, *CCL7*, *CCL19*, *CCR2*), pathways and biological processes, involved in the differential response of human PBMCs and dendritic cells to measles infection or viral stimulation.[46,47,48] Intriguing is the facts that several of the highlighted genes/proteins (i.e., *IL6*, *CXCL12*, and *CD93*) are known potent survival signals for the maintenance of plasma cells in the bone marrow and thus their expression may have a direct impact on the regulation of long-term humoral immune responses and immune memory.[44]

The overlap between our data-driven gene-to-biology analysis approach and our biology-to-gene knowledge-driven geneset approach indicates and supports the major involvement of six genes in the differential transcriptional responses observed in the two extreme antibody response groups. These include *CD93* (discussed above); *CD33* and *CADM1*, involved in cell adhesion, regulation of proliferation and signal transduction; *VEGFA*, an important cell growth factor; *VNN1*, involved in T cell migration and the response to oxidative stress; and *IL10*, encoding a cytokine with pleiotropic immunosuppressive and immunomodulatory properties, which affects antigen presentation and both T and B cell function (including antibody production).[49,50]

To the best of our knowledge, this is the first study examining and characterizing genome-wide transcriptional responses (using unbiased mRNA-Seq technology) to measles virus in PBMCs of measles-vaccine recipients, which represents the extremes of the neutralizing antibody response after vaccination. Current state-of-the-art paradigms advocate for performing a comprehensive series of unbiased systems biology studies in order to thoroughly define immune/molecular signatures of immune responses. [5,6,7] In this work, we identified immune signatures responsible for (or indicative of) the maintenance of antigen-specific bone marrow plasma cells and development/maintenance of adaptive B cell immune responses to measles vaccination. Due to sample availability, our study design did not include a longitudinal sampling (before and after vaccination), nor the integration of multiple omics-level data into predictive models of vaccine response). At this early stage of knowledge, the current results and identified genes/pathways provide the framework for future systems-level studies in order to better understand measles vaccine-induced immunity.

Two major strengths of our study are the collection of unbiased high-quality mRNA-Seq data from samples of a well-characterized cohort of measles vaccine recipients (after two doses of MMR vaccine and no wild type measles virus exposure), and our statistical (gene-to-biology and biology-to-gene) and bioinformatics approaches, which enabled us to identify specific genes (e.g., *CD93*, *IL24*, *IL6*, *CXCL12*), pathways and processes (e.g., cell adhesion and migration, cytokine/chemokine activity and regulation, inflammatory response) and network components that are biologically relevant. The major limitation of the study is the possibility of false-positive associations of transcriptional changes with antibody titers. To guide interpretation and help evaluate the level of evidence, we report both p-values and q-values (for the per-variable analysis), and use a complementary knowledge-driven biology-to-gene approach (as part of our analysis) to control for false-discovery rate. In addition, the interpretability of our findings should be the context of *in vitro* PBMCs re-stimulation post-vaccination and may not necessarily reflect gene expression is different cell populations *in vivo* after vaccination. Our findings are novel, however, as this is the first reported mRNA-Seq gene expression study assessing gene expression in cells from high and low measles vaccine responders. We believe the differential ability of the cells/PBMCs (from high and low responders) to respond to viral stimulation may reflect differences in cell homing, adhesion, and cell survival of specific cell subtypes (e.g., plasma cells) and, indirectly, antibody titer. However, validation of the identified genes/pathways in a larger measles vaccine systems biology study (including assessment of gene expression in specific cell subtypes) is necessary and is underway in our laboratory.

In conclusion, we report the first comprehensive transcriptome-level characterization (mRNA-Seq data) of responses to measles virus stimulation in high and low antibody responders to measles vaccination, using vaccinomics, statistical and network biology approaches to define plausible regulators (genes/pathways/networks) that drive and/or underlie the observed differences in neutralizing antibody titers after measles vaccination. Studies such as ours enable us to explain how specific markers of adaptive (or innate) immune response are influenced by transcriptional (or other) changes dues to vaccination, and to develop a panel of biomarkers or models that predict and explain the immune response to measles vaccine.

## Supporting Information

S1 Table Immune response variables of the study subjects. (DOCX)

S2 Table Significant genes based on overall response to measles virus stimulation in all vaccine recipients (1,761 significant genes with FC>2 and FC <0.5). (DOCX)

S3 Table Significant genesets, in high vs low antibody vaccine responders (response to viral stimulation) (DOCX)
