# Supplementary material for: Whole Transcriptome Profiling Identifies CD93 and Other Plasma Cell Survival Factor Genes Associated with Measles-Specific Antibody Response after Vaccination
Source: PLoS One. 2016 Aug 16;11(8):e0160970. doi: 10.1371/journal.pone.0160970 (PMC4987012; doi:10.1371/journal.pone.0160970)
Supplement: S1 Table — (DOCX) [file pone.0160970.s001.docx]

**S1 Table.** Immune response variables of the study subjects

| **Immune**  **Outcome** | **Ab Response Category** | **Median (IQR)**^a^ | **p-value**^b^ |
| --- | --- | --- | --- |
| IFNα | High | 284 (171, 805) | 0.075 |
|  | Low | 579 (443, 912) |  |
| IFNλ1 | High  Low | 15 (-10, 41)  31 (12, 73) | 0.062 |
| IFNγ | High | 54 (39, 121) | 0.740 |
|  | Low | 58 (21, 95) |  |
| IL-10 | High | 18 (11, 57) | 0.362 |
|  | Low | 15 (11, 22) |  |
| IL-2 | High | 42 (12, 59) | 0.901 |
|  | Low | 37 (23, 70) |  |
| IL-6 | High | 325 (153, 518) | 0.590 |
|  | Low | 365 (286, 474) |  |
| TNFα | High | 18 (11, 26) | 0.062 |
|  | Low | 8 ( 4, 21) |  |
| IFNγ Elispot | High | 24 (9, 44) | 0.965 |
| SFU x 200000 cells | Low | 44 (3, 77) |  |
| Antibody (PRMN,mIU/mL) | High | 5188 (4594, 5365) | NA^c^ |
|  | Low | 88 (58, 115) |  |

^a^Values are in mIU/mL for antibody titers (PRMN), cytokine spot-forming units (SFUs) per 2 x 10^5^ cells for Elispot responses and pg/mL for cytokines responses ± IQR, inter-quartile range with 25% and 75% quartiles

Elispot response and cytokine response is defined as the median measles virus-stimulated response for each subject (from triplicate measurements) minus the median unstimulated response (also from triplicate measurements). Negative values indicate that stimulated values were on average smaller than unstimulated values.

^b^P-values were calculated using Wilcoxon Rank Sum test

^c^Not applicable, subjects were selected based on high/low antibody titer.
