# Supplementary material for: Whole Transcriptome Profiling Identifies CD93 and Other Plasma Cell Survival Factor Genes Associated with Measles-Specific Antibody Response after Vaccination
Source: PLoS One. 2016 Aug 16;11(8):e0160970. doi: 10.1371/journal.pone.0160970 (PMC4987012; doi:10.1371/journal.pone.0160970)
Supplement: S2 Table — (DOCX) [file pone.0160970.s002.docx]

**S2 Table.** Significant genes based on overall response to measles virus stimulation in all vaccine recipients (1,761 significant genes with FC>2 and FC <0.5).

| **Gene**  **symbol** | **FC low**^a^ | **FC low log2**^b^ | **FC high^c^** | **FC high log2^d^** | **FC**  **overall^e^** | **FC overall log2^f^** | **p-value^g^** |
| --- | --- | --- | --- | --- | --- | --- | --- |
| *VSIG4* | 0.01 | -6.38 | 0.01 | -6.70 | 0.01 | -6.54 | <1.00E-259 |
| *COL23A1* | 0.06 | -4.04 | 0.07 | -3.78 | 0.07 | -3.91 | <1.00E-259 |
| *SNX30* | 0.30 | -1.73 | 0.32 | -1.64 | 0.31 | -1.69 | <1.00E-259 |
| *MYO16* | 0.89 | -0.18 | 0.97 | -0.04 | 4186.57 | 12.03 | <1.00E-259 |
| *TSPO* | 0.39 | -1.36 | 0.41 | -1.27 | 0.40 | -1.31 | 1.19E-259 |
| *PARP3* | 2.85 | 1.51 | 2.55 | 1.35 | 2.69 | 1.43 | 2.36E-246 |
| *FCN1* | 0.12 | -3.05 | 0.13 | -2.96 | 0.12 | -3.00 | 1.13E-216 |
| *TRADD* | 2.22 | 1.15 | 2.15 | 1.10 | 2.18 | 1.13 | 2.73E-205 |
| *CMTM4* | 0.39 | -1.38 | 0.40 | -1.34 | 0.39 | -1.36 | 1.16E-204 |
| *GPR113* | 2.44 | 1.29 | 2.36 | 1.24 | 2.40 | 1.26 | 3.71E-204 |
| *CPVL* | 0.14 | -2.82 | 0.17 | -2.57 | 0.15 | -2.70 | 1.42E-203 |
| *GLIPR1* | 0.22 | -2.17 | 0.24 | -2.08 | 0.23 | -2.13 | 1.71E-202 |
| *IQCD* | 0.08 | -3.56 | 0.10 | -3.37 | 0.09 | -3.47 | 2.10E-198 |
| *LYZ* | 0.24 | -2.05 | 0.22 | -2.21 | 0.23 | -2.13 | 2.34E-193 |
| *S100A4* | 0.08 | -3.69 | 0.09 | -3.40 | 0.09 | -3.55 | 3.21E-192 |
| *FCGRT* | 0.22 | -2.18 | 0.23 | -2.11 | 0.23 | -2.15 | 1.06E-190 |
| *CALM2* | 0.43 | -1.23 | 0.44 | -1.19 | 0.43 | -1.21 | 8.52E-189 |
| *INSR* | 0.18 | -2.44 | 0.24 | -2.08 | 0.21 | -2.26 | 1.77E-186 |
| *ZBTB32* | 5.09 | 2.35 | 5.06 | 2.34 | 5.07 | 2.34 | 2.49E-186 |
| *TK2* | 0.26 | -1.92 | 0.32 | -1.63 | 0.29 | -1.77 | 2.56E-186 |
| *TLR5* | 0.23 | -2.14 | 0.24 | -2.08 | 0.23 | -2.11 | 1.30E-182 |
| *RNASE6* | 0.08 | -3.69 | 0.08 | -3.71 | 0.08 | -3.70 | 7.75E-179 |
| *PRR5* | 4.34 | 2.12 | 4.05 | 2.02 | 4.19 | 2.07 | 7.84E-179 |
| *CTNNBIP1* | 0.31 | -1.68 | 0.35 | -1.52 | 0.33 | -1.60 | 1.72E-176 |
| *TPCN1* | 0.16 | -2.66 | 0.18 | -2.47 | 0.17 | -2.57 | 7.90E-176 |
| *TREM2* | 0.02 | -5.65 | 0.02 | -5.70 | 0.02 | -5.68 | 2.98E-175 |
| *APLP2* | 0.31 | -1.69 | 0.32 | -1.62 | 0.32 | -1.66 | 4.62E-175 |
| *HCST* | 0.42 | -1.25 | 0.45 | -1.16 | 0.43 | -1.20 | 5.18E-175 |
| *PMAIP1* | 5.37 | 2.42 | 4.23 | 2.08 | 4.77 | 2.25 | 2.40E-172 |
| *ELMO2* | 2.35 | 1.23 | 2.18 | 1.12 | 2.26 | 1.18 | 1.85E-169 |
| *IFNGR1* | 0.25 | -2.00 | 0.29 | -1.81 | 0.27 | -1.91 | 3.18E-169 |
| *ARRB2* | 0.36 | -1.47 | 0.40 | -1.32 | 0.38 | -1.40 | 3.93E-169 |
| *LOC728743* | 2.48 | 1.31 | 2.29 | 1.19 | 2.38 | 1.25 | 6.79E-169 |
| *SLC46A1* | 0.10 | -3.29 | 0.14 | -2.86 | 0.12 | -3.07 | 2.24E-167 |
| *RNF130* | 0.36 | -1.48 | 0.40 | -1.32 | 0.38 | -1.40 | 5.44E-167 |
| *GGTA1P* | 0.01 | -6.24 | 0.02 | -5.96 | 0.01 | -6.10 | 2.98E-166 |
| *FAM173B* | 0.42 | -1.25 | 0.42 | -1.27 | 0.42 | -1.26 | 3.89E-164 |
| *RASSF2* | 0.27 | -1.90 | 0.30 | -1.74 | 0.28 | -1.82 | 2.55E-163 |
| *ATP6AP2* | 0.24 | -2.06 | 0.28 | -1.85 | 0.26 | -1.96 | 2.08E-162 |
| *TMEM159* | 2.14 | 1.09 | 2.06 | 1.04 | 2.10 | 1.07 | 1.97E-159 |
| *AKR7A2* | 0.49 | -1.04 | 0.51 | -0.96 | 0.50 | -1.00 | 2.82E-159 |
| *LPPR2* | 0.35 | -1.53 | 0.35 | -1.50 | 0.35 | -1.52 | 6.55E-159 |
| *C2orf65* | 0.19 | -2.41 | 0.21 | -2.23 | 0.20 | -2.32 | 1.20E-158 |
| *TBXAS1* | 0.06 | -4.17 | 0.08 | -3.64 | 0.07 | -3.90 | 4.97E-156 |
| *GPNMB* | 0.17 | -2.58 | 0.18 | -2.51 | 0.17 | -2.55 | 3.08E-155 |
| *TMED10* | 0.48 | -1.05 | 0.51 | -0.97 | 0.50 | -1.01 | 2.77E-152 |
| *SKAP2* | 0.45 | -1.16 | 0.47 | -1.10 | 0.46 | -1.13 | 1.27E-151 |
| *TSPAN5* | 2.11 | 1.08 | 1.99 | 0.99 | 2.05 | 1.03 | 2.09E-151 |
| *ARHGEF3* | 2.12 | 1.09 | 2.02 | 1.02 | 2.07 | 1.05 | 4.62E-151 |
| *FAM198B* | 0.19 | -2.42 | 0.18 | -2.45 | 0.18 | -2.44 | 6.90E-151 |
| *ARRB1* | 0.29 | -1.78 | 0.35 | -1.53 | 0.32 | -1.66 | 8.91E-151 |
| *PPM1M* | 0.44 | -1.18 | 0.44 | -1.20 | 0.44 | -1.19 | 1.59E-150 |
| *ZNRF2* | 3.76 | 1.91 | 3.49 | 1.80 | 3.62 | 1.86 | 1.85E-150 |
| *MAN2B1* | 0.45 | -1.17 | 0.48 | -1.06 | 0.46 | -1.11 | 3.09E-150 |
| *H2AFY* | 0.48 | -1.06 | 0.50 | -1.01 | 0.49 | -1.03 | 2.79E-149 |
| *MST1R* | 4.13 | 2.04 | 3.50 | 1.81 | 3.80 | 1.93 | 1.46E-147 |
| *DEF8* | 0.47 | -1.09 | 0.48 | -1.04 | 0.48 | -1.07 | 1.55E-147 |
| *FLJ90757* | 0.29 | -1.81 | 0.29 | -1.78 | 0.29 | -1.79 | 1.60E-146 |
| *PRR5-ARHGAP8* | 4.01 | 2.00 | 3.75 | 1.91 | 3.88 | 1.96 | 1.23E-145 |
| *APOL2* | 3.70 | 1.89 | 3.41 | 1.77 | 3.55 | 1.83 | 3.73E-145 |
| *CEACAM1* | 4.56 | 2.19 | 4.10 | 2.04 | 4.32 | 2.11 | 7.35E-145 |
| *RAB3D* | 0.19 | -2.36 | 0.23 | -2.12 | 0.21 | -2.24 | 2.11E-144 |
| *APOL3* | 4.18 | 2.06 | 4.16 | 2.06 | 4.17 | 2.06 | 5.65E-144 |
| *CRTAP* | 0.32 | -1.63 | 0.36 | -1.48 | 0.34 | -1.56 | 8.83E-142 |
| *PECAM1* | 0.25 | -2.02 | 0.27 | -1.91 | 0.26 | -1.97 | 1.72E-141 |
| *ASRGL1* | 0.14 | -2.86 | 0.16 | -2.64 | 0.15 | -2.75 | 9.30E-141 |
| *TBC1D14* | 0.35 | -1.50 | 0.39 | -1.37 | 0.37 | -1.44 | 1.56E-140 |
| *SCIN* | 15.54 | 3.96 | 11.62 | 3.54 | 13.44 | 3.75 | 5.61E-140 |
| *FKBP1A* | 0.45 | -1.14 | 0.49 | -1.03 | 0.47 | -1.09 | 1.71E-139 |
| *SLC4A7* | 0.44 | -1.19 | 0.45 | -1.14 | 0.45 | -1.17 | 2.88E-138 |
| *PAPD7* | 3.18 | 1.67 | 2.81 | 1.49 | 2.99 | 1.58 | 3.42E-138 |
| *USP42* | 2.36 | 1.24 | 2.16 | 1.11 | 2.26 | 1.18 | 4.11E-138 |
| *HELB* | 2.72 | 1.44 | 2.61 | 1.38 | 2.66 | 1.41 | 2.31E-137 |
| *SSTR2* | 29.95 | 4.90 | 28.05 | 4.81 | 28.98 | 4.86 | 2.13E-136 |
| *KIAA0408* | 3.32 | 1.73 | 3.06 | 1.61 | 3.19 | 1.67 | 6.96E-136 |
| *COTL1* | 0.32 | -1.63 | 0.33 | -1.59 | 0.33 | -1.61 | 5.46E-135 |
| *PRADC1* | 0.40 | -1.33 | 0.44 | -1.18 | 0.42 | -1.26 | 5.46E-135 |
| *NBN* | 3.57 | 1.83 | 3.37 | 1.75 | 3.47 | 1.79 | 6.74E-135 |
| *SLC37A1* | 3.50 | 1.81 | 3.06 | 1.61 | 3.27 | 1.71 | 9.64E-135 |
| *MDGA1* | 3.62 | 1.86 | 2.99 | 1.58 | 3.27 | 1.71 | 2.69E-134 |
| *PCDH1* | 0.23 | -2.13 | 0.26 | -1.95 | 0.24 | -2.04 | 2.77E-134 |
| *DAD1* | 0.48 | -1.05 | 0.50 | -0.99 | 0.49 | -1.02 | 2.80E-134 |
| *LRP3* | 0.15 | -2.69 | 0.18 | -2.51 | 0.16 | -2.60 | 5.25E-134 |
| *C20orf108* | 0.44 | -1.17 | 0.47 | -1.09 | 0.46 | -1.13 | 1.27E-133 |
| *IMPA2* | 0.21 | -2.25 | 0.26 | -1.96 | 0.23 | -2.10 | 1.94E-133 |
| *SH3BGRL3* | 0.45 | -1.15 | 0.46 | -1.13 | 0.45 | -1.14 | 2.54E-133 |
| *PRRT2* | 3.46 | 1.79 | 3.48 | 1.80 | 3.47 | 1.80 | 3.27E-133 |
| *UBE2Z* | 2.51 | 1.33 | 2.33 | 1.22 | 2.42 | 1.27 | 3.93E-133 |
| *STBD1* | 4.47 | 2.16 | 4.13 | 2.05 | 4.30 | 2.10 | 1.37E-132 |
| *ARMCX1* | 3.62 | 1.86 | 3.74 | 1.90 | 3.68 | 1.88 | 1.47E-132 |
| *COL6A3* | 0.09 | -3.49 | 0.11 | -3.19 | 0.10 | -3.34 | 2.12E-132 |
| *STARD13* | 0.19 | -2.40 | 0.19 | -2.40 | 0.19 | -2.40 | 2.38E-132 |
| *CYBRD1* | 0.18 | -2.51 | 0.21 | -2.24 | 0.19 | -2.38 | 4.60E-132 |
| *AHNAK* | 0.36 | -1.48 | 0.39 | -1.35 | 0.37 | -1.42 | 1.43E-131 |
| *MATK* | 0.37 | -1.44 | 0.35 | -1.51 | 0.36 | -1.48 | 2.18E-131 |
| *KDM6A* | 2.15 | 1.11 | 1.94 | 0.96 | 2.04 | 1.03 | 3.14E-131 |
| *CRYL1* | 0.28 | -1.84 | 0.34 | -1.57 | 0.31 | -1.71 | 7.46E-130 |
| *ITGB5* | 0.13 | -2.99 | 0.17 | -2.54 | 0.15 | -2.77 | 1.13E-128 |
| *EEF2K* | 0.37 | -1.43 | 0.42 | -1.25 | 0.40 | -1.34 | 1.33E-128 |
| *P2RX4* | 4.29 | 2.10 | 3.37 | 1.75 | 3.80 | 1.93 | 1.46E-128 |
| *RPS3A* | 0.46 | -1.13 | 0.49 | -1.03 | 0.47 | -1.08 | 2.52E-128 |
| *SFRP5* | 0.24 | -2.04 | 0.25 | -2.00 | 0.25 | -2.02 | 2.62E-128 |
| *PPT1* | 0.23 | -2.14 | 0.25 | -2.00 | 0.24 | -2.07 | 4.09E-128 |
| *DYNC2H1* | 0.43 | -1.21 | 0.48 | -1.06 | 0.46 | -1.13 | 5.12E-128 |
| *XYLB* | 0.25 | -1.98 | 0.30 | -1.75 | 0.27 | -1.86 | 3.87E-127 |
| *C1orf162* | 0.17 | -2.57 | 0.16 | -2.61 | 0.17 | -2.59 | 4.85E-127 |
| *DPYD* | 0.45 | -1.15 | 0.48 | -1.07 | 0.46 | -1.11 | 8.11E-127 |
| *RCN3* | 0.19 | -2.41 | 0.19 | -2.41 | 0.19 | -2.41 | 1.20E-124 |
| *FNIP2* | 4.11 | 2.04 | 3.44 | 1.78 | 3.76 | 1.91 | 2.17E-124 |
| *ARHGEF4* | 0.24 | -2.04 | 0.28 | -1.82 | 0.26 | -1.93 | 4.73E-124 |
| *ZSWIM5* | 3.44 | 1.78 | 2.68 | 1.42 | 3.04 | 1.60 | 4.88E-124 |
| *HLA-DMB* | 0.17 | -2.53 | 0.18 | -2.45 | 0.18 | -2.49 | 7.45E-124 |
| *CCNA1* | 53.02 | 5.73 | 41.57 | 5.38 | 46.94 | 5.55 | 3.36E-123 |
| *TICAM1* | 2.71 | 1.44 | 2.50 | 1.32 | 2.60 | 1.38 | 1.24E-122 |
| *SIGLEC7* | 0.13 | -2.93 | 0.17 | -2.60 | 0.15 | -2.77 | 2.11E-122 |
| *OPTN* | 2.66 | 1.41 | 2.35 | 1.23 | 2.50 | 1.32 | 6.14E-122 |
| *DOPEY1* | 2.29 | 1.20 | 2.28 | 1.19 | 2.29 | 1.19 | 6.29E-122 |
| *SDK2* | 0.19 | -2.37 | 0.25 | -1.99 | 0.22 | -2.18 | 9.83E-122 |
| *CEBPA* | 0.16 | -2.64 | 0.20 | -2.35 | 0.18 | -2.49 | 1.21E-121 |
| *FAS* | 3.80 | 1.93 | 3.42 | 1.77 | 3.61 | 1.85 | 5.94E-121 |
| *APOBEC3F* | 2.73 | 1.45 | 2.42 | 1.27 | 2.57 | 1.36 | 1.32E-120 |
| *PSAT1* | 3.71 | 1.89 | 3.42 | 1.77 | 3.56 | 1.83 | 1.95E-120 |
| *ATP5F1* | 0.48 | -1.07 | 0.50 | -1.00 | 0.49 | -1.03 | 2.49E-120 |
| *IPCEF1* | 2.33 | 1.22 | 2.25 | 1.17 | 2.29 | 1.19 | 2.97E-120 |
| *TNFAIP8L2* | 0.46 | -1.13 | 0.50 | -1.00 | 0.48 | -1.07 | 1.23E-119 |
| *LOC100131733* | 4.69 | 2.23 | 4.06 | 2.02 | 4.36 | 2.12 | 2.11E-119 |
| *BCL2L13* | 2.24 | 1.16 | 2.10 | 1.07 | 2.17 | 1.12 | 3.28E-119 |
| *ASB13* | 0.29 | -1.78 | 0.35 | -1.53 | 0.32 | -1.66 | 1.30E-118 |
| *ABP1* | 0.20 | -2.34 | 0.22 | -2.15 | 0.21 | -2.24 | 1.43E-118 |
| *KCNAB2* | 0.40 | -1.31 | 0.44 | -1.20 | 0.42 | -1.25 | 2.23E-118 |
| *PDGFC* | 0.26 | -1.95 | 0.26 | -1.94 | 0.26 | -1.94 | 2.69E-118 |
| *LOC80054* | 0.06 | -4.15 | 0.08 | -3.66 | 0.07 | -3.90 | 5.01E-118 |
| *P2RX7* | 6.42 | 2.68 | 5.07 | 2.34 | 5.71 | 2.51 | 6.93E-118 |
| *LOC730102* | 0.13 | -2.96 | 0.15 | -2.73 | 0.14 | -2.84 | 1.24E-117 |
| *SUPT3H* | 2.13 | 1.09 | 1.91 | 0.93 | 2.02 | 1.01 | 1.28E-117 |
| *TNFRSF13B* | 2.33 | 1.22 | 2.15 | 1.10 | 2.24 | 1.16 | 4.80E-117 |
| *DNAJB4* | 2.80 | 1.49 | 2.47 | 1.30 | 2.63 | 1.39 | 7.58E-117 |
| *CASP3* | 2.63 | 1.40 | 2.34 | 1.23 | 2.48 | 1.31 | 8.08E-117 |
| *MMP28* | 0.13 | -2.91 | 0.15 | -2.76 | 0.14 | -2.84 | 8.16E-117 |
| *FASLG* | 2.93 | 1.55 | 2.75 | 1.46 | 2.84 | 1.51 | 1.45E-116 |
| *KCNQ1* | 0.34 | -1.57 | 0.36 | -1.48 | 0.35 | -1.53 | 1.50E-116 |
| *KCTD15* | 0.17 | -2.56 | 0.25 | -2.02 | 0.20 | -2.29 | 1.02E-115 |
| *PTGS1* | 0.33 | -1.61 | 0.33 | -1.59 | 0.33 | -1.60 | 1.14E-115 |
| *C10orf11* | 0.20 | -2.35 | 0.24 | -2.08 | 0.22 | -2.22 | 1.16E-115 |
| *RAB37* | 0.30 | -1.72 | 0.31 | -1.70 | 0.31 | -1.71 | 1.75E-115 |
| *C6orf174* | 4.51 | 2.17 | 3.67 | 1.87 | 4.07 | 2.02 | 1.87E-115 |
| *ARHGAP4* | 0.44 | -1.19 | 0.47 | -1.09 | 0.45 | -1.14 | 2.15E-115 |
| *PCYOX1* | 0.39 | -1.36 | 0.42 | -1.24 | 0.41 | -1.30 | 2.35E-115 |
| *ABCC3* | 0.26 | -1.96 | 0.27 | -1.87 | 0.27 | -1.92 | 2.89E-115 |
| *ADCY4* | 4.34 | 2.12 | 3.47 | 1.79 | 3.88 | 1.96 | 5.59E-115 |
| *EDN1* | 14.94 | 3.90 | 14.38 | 3.85 | 14.66 | 3.87 | 5.62E-115 |
| *AP1S2* | 0.31 | -1.68 | 0.37 | -1.45 | 0.34 | -1.56 | 1.95E-114 |
| *ZDHHC7* | 0.43 | -1.22 | 0.45 | -1.17 | 0.44 | -1.19 | 7.16E-113 |
| *ARAP3* | 0.40 | -1.32 | 0.42 | -1.27 | 0.41 | -1.29 | 1.67E-112 |
| *TIMP2* | 0.32 | -1.64 | 0.33 | -1.61 | 0.32 | -1.62 | 2.25E-112 |
| *NFIA* | 0.21 | -2.26 | 0.28 | -1.83 | 0.24 | -2.05 | 3.78E-112 |
| *UBXN11* | 0.19 | -2.38 | 0.21 | -2.23 | 0.20 | -2.31 | 4.28E-112 |
| *RHEBL1* | 3.61 | 1.85 | 3.41 | 1.77 | 3.51 | 1.81 | 8.02E-112 |
| *RIPK2* | 5.48 | 2.46 | 4.30 | 2.11 | 4.86 | 2.28 | 2.84E-111 |
| *LTA* | 3.31 | 1.73 | 2.73 | 1.45 | 3.01 | 1.59 | 3.83E-111 |
| *TMEM140* | 4.24 | 2.08 | 3.73 | 1.90 | 3.98 | 1.99 | 7.17E-111 |
| *STXBP1* | 0.25 | -1.97 | 0.29 | -1.80 | 0.27 | -1.89 | 8.59E-111 |
| *LTA4H* | 0.14 | -2.85 | 0.14 | -2.81 | 0.14 | -2.83 | 2.31E-110 |
| *PCGF5* | 2.13 | 1.09 | 1.99 | 0.99 | 2.06 | 1.04 | 3.32E-110 |
| *B4GALT5* | 4.39 | 2.14 | 3.50 | 1.81 | 3.92 | 1.97 | 3.84E-110 |
| *SLC6A9* | 10.33 | 3.37 | 7.61 | 2.93 | 8.87 | 3.15 | 7.61E-110 |
| *RGS12* | 0.32 | -1.64 | 0.39 | -1.37 | 0.35 | -1.50 | 1.30E-109 |
| *TMEM170B* | 0.36 | -1.46 | 0.32 | -1.63 | 0.34 | -1.55 | 6.12E-109 |
| *PFDN5* | 0.45 | -1.15 | 0.45 | -1.14 | 0.45 | -1.15 | 6.57E-109 |
| *BTN2A3* | 2.03 | 1.02 | 2.03 | 1.02 | 2.03 | 1.02 | 8.82E-109 |
| *LOC100505746* | 0.19 | -2.38 | 0.21 | -2.27 | 0.20 | -2.32 | 1.65E-108 |
| *TMEM110* | 2.26 | 1.17 | 2.21 | 1.14 | 2.23 | 1.16 | 1.68E-108 |
| *VSIG1* | 0.38 | -1.39 | 0.41 | -1.29 | 0.39 | -1.34 | 2.85E-108 |
| *CD4* | 0.25 | -1.99 | 0.30 | -1.72 | 0.28 | -1.86 | 8.76E-108 |
| *PYGL* | 0.33 | -1.59 | 0.31 | -1.68 | 0.32 | -1.64 | 2.19E-107 |
| *CD300LB* | 0.06 | -3.97 | 0.09 | -3.51 | 0.07 | -3.74 | 2.48E-107 |
| *ELF4* | 2.57 | 1.36 | 2.36 | 1.24 | 2.46 | 1.30 | 2.53E-107 |
| *CBX7* | 0.29 | -1.81 | 0.33 | -1.58 | 0.31 | -1.69 | 2.69E-107 |
| *ANXA9* | 0.22 | -2.20 | 0.27 | -1.91 | 0.24 | -2.05 | 2.79E-107 |
| *SETD7* | 0.38 | -1.40 | 0.45 | -1.15 | 0.41 | -1.28 | 4.62E-107 |
| *AMBRA1* | 2.44 | 1.29 | 2.18 | 1.13 | 2.31 | 1.21 | 4.79E-107 |
| *SIRPB2* | 0.21 | -2.24 | 0.23 | -2.11 | 0.22 | -2.18 | 4.96E-107 |
| *CD9* | 0.10 | -3.36 | 0.07 | -3.74 | 0.09 | -3.55 | 5.88E-107 |
| *EIF3L* | 0.30 | -1.74 | 0.34 | -1.57 | 0.32 | -1.66 | 8.17E-106 |
| *CDHR1* | 0.24 | -2.04 | 0.28 | -1.82 | 0.26 | -1.93 | 2.98E-105 |
| *KIF19* | 6.27 | 2.65 | 4.70 | 2.23 | 5.43 | 2.44 | 1.22E-104 |
| *ARHGDIB* | 0.49 | -1.04 | 0.48 | -1.07 | 0.48 | -1.06 | 3.27E-104 |
| *PLBD1* | 0.20 | -2.29 | 0.22 | -2.19 | 0.21 | -2.24 | 3.38E-104 |
| *CLCN5* | 0.37 | -1.43 | 0.43 | -1.23 | 0.40 | -1.33 | 5.79E-104 |
| *IL12RB2* | 3.64 | 1.86 | 3.22 | 1.68 | 3.42 | 1.77 | 9.26E-104 |
| *SNX29* | 0.42 | -1.23 | 0.47 | -1.10 | 0.44 | -1.17 | 3.34E-103 |
| *HLA-DMA* | 0.26 | -1.95 | 0.28 | -1.83 | 0.27 | -1.89 | 4.14E-103 |
| *CD101* | 0.09 | -3.47 | 0.12 | -3.10 | 0.10 | -3.29 | 7.09E-103 |
| *GPR34* | 0.04 | -4.73 | 0.05 | -4.22 | 0.04 | -4.48 | 2.30E-102 |
| *LOC440461* | 3.01 | 1.59 | 2.73 | 1.45 | 2.87 | 1.52 | 4.24E-102 |
| *KDELC2* | 2.18 | 1.13 | 2.00 | 1.00 | 2.09 | 1.06 | 7.07E-102 |
| *LOXL3* | 0.39 | -1.35 | 0.39 | -1.35 | 0.39 | -1.35 | 9.78E-102 |
| *LOC729178* | 0.25 | -2.00 | 0.24 | -2.07 | 0.24 | -2.03 | 1.71E-101 |
| *CAMK1* | 0.23 | -2.10 | 0.25 | -2.02 | 0.24 | -2.06 | 2.64E-101 |
| *GNAQ* | 0.46 | -1.11 | 0.49 | -1.02 | 0.48 | -1.06 | 5.10E-101 |
| *WDFY1* | 2.46 | 1.30 | 2.29 | 1.19 | 2.37 | 1.24 | 5.25E-101 |
| *MARVELD1* | 0.20 | -2.31 | 0.23 | -2.10 | 0.22 | -2.21 | 7.91E-101 |
| *GLT25D1* | 0.30 | -1.73 | 0.34 | -1.56 | 0.32 | -1.64 | 9.01E-101 |
| *GFRA2* | 0.08 | -3.66 | 0.10 | -3.39 | 0.09 | -3.52 | 2.26E-100 |
| *RPL17* | 0.45 | -1.15 | 0.47 | -1.09 | 0.46 | -1.12 | 2.28E-100 |
| *SYK* | 0.42 | -1.26 | 0.44 | -1.19 | 0.43 | -1.22 | 9.59E-100 |
| *PFKP* | 2.19 | 1.13 | 2.07 | 1.05 | 2.13 | 1.09 | 1.09E-99 |
| *ADI1* | 0.40 | -1.34 | 0.45 | -1.16 | 0.42 | -1.25 | 2.60E-99 |
| *PC* | 0.19 | -2.41 | 0.22 | -2.19 | 0.20 | -2.30 | 5.42E-99 |
| *IGFBP4* | 14.15 | 3.82 | 11.88 | 3.57 | 12.97 | 3.70 | 5.58E-99 |
| *RASA4* | 0.49 | -1.03 | 0.50 | -1.00 | 0.49 | -1.02 | 1.94E-98 |
| *DIP2C* | 2.64 | 1.40 | 2.59 | 1.37 | 2.61 | 1.39 | 2.05E-98 |
| *C11orf45* | 0.27 | -1.90 | 0.26 | -1.93 | 0.27 | -1.91 | 5.52E-98 |
| *SBK1* | 2.59 | 1.37 | 2.59 | 1.37 | 2.59 | 1.37 | 7.59E-98 |
| *HBD* | 5.63 | 2.49 | 4.93 | 2.30 | 5.27 | 2.40 | 1.21E-97 |
| *SLC25A28* | 2.85 | 1.51 | 2.72 | 1.45 | 2.79 | 1.48 | 1.28E-97 |
| *SLC46A2* | 0.18 | -2.49 | 0.17 | -2.56 | 0.17 | -2.52 | 1.31E-97 |
| *CERK* | 0.40 | -1.32 | 0.44 | -1.20 | 0.42 | -1.26 | 1.67E-97 |
| *IRF2* | 2.49 | 1.31 | 2.25 | 1.17 | 2.37 | 1.24 | 1.91E-97 |
| *SUMF1* | 0.43 | -1.20 | 0.46 | -1.11 | 0.45 | -1.16 | 1.97E-97 |
| *SAMD1* | 0.42 | -1.25 | 0.48 | -1.06 | 0.45 | -1.15 | 3.00E-97 |
| *METTL7A* | 0.22 | -2.22 | 0.25 | -2.02 | 0.23 | -2.12 | 5.01E-97 |
| *C8orf83* | 0.47 | -1.08 | 0.53 | -0.92 | 0.50 | -1.00 | 5.35E-97 |
| *RASAL1* | 0.12 | -3.02 | 0.15 | -2.73 | 0.14 | -2.87 | 5.59E-97 |
| *CNN2* | 0.48 | -1.05 | 0.51 | -0.98 | 0.49 | -1.02 | 9.98E-97 |
| *TGM1* | 5.82 | 2.54 | 5.16 | 2.37 | 5.48 | 2.46 | 1.06E-96 |
| *HAPLN3* | 3.30 | 1.72 | 3.43 | 1.78 | 3.36 | 1.75 | 1.14E-96 |
| *TMPRSS13* | 4.65 | 2.22 | 4.24 | 2.08 | 4.44 | 2.15 | 1.23E-96 |
| *PTGFRN* | 0.04 | -4.54 | 0.04 | -4.58 | 0.04 | -4.56 | 1.35E-96 |
| *CR1* | 0.20 | -2.34 | 0.20 | -2.29 | 0.20 | -2.31 | 1.99E-96 |
| *DENND1B* | 4.72 | 2.24 | 3.98 | 1.99 | 4.33 | 2.12 | 2.07E-96 |
| *SIDT1* | 2.25 | 1.17 | 2.04 | 1.03 | 2.15 | 1.10 | 2.96E-96 |
| *CCDC146* | 3.26 | 1.70 | 2.79 | 1.48 | 3.01 | 1.59 | 5.63E-96 |
| *NDC80* | 2.94 | 1.55 | 2.68 | 1.42 | 2.81 | 1.49 | 2.81E-95 |
| *LPIN2* | 2.13 | 1.09 | 2.00 | 1.00 | 2.06 | 1.05 | 7.55E-95 |
| *PLB1* | 0.09 | -3.52 | 0.10 | -3.26 | 0.10 | -3.39 | 7.66E-95 |
| *CLMN* | 0.32 | -1.65 | 0.32 | -1.63 | 0.32 | -1.64 | 2.26E-94 |
| *TRIM38* | 2.56 | 1.35 | 2.36 | 1.24 | 2.45 | 1.29 | 2.51E-94 |
| *AIFM2* | 2.94 | 1.55 | 2.96 | 1.57 | 2.95 | 1.56 | 2.56E-94 |
| *ACOT11* | 0.07 | -3.93 | 0.08 | -3.70 | 0.07 | -3.81 | 2.75E-94 |
| *LOC79015* | 12.11 | 3.60 | 8.43 | 3.07 | 10.10 | 3.34 | 3.15E-94 |
| *OPRL1* | 0.48 | -1.06 | 0.49 | -1.04 | 0.48 | -1.05 | 3.49E-94 |
| *CCDC42* | 3.07 | 1.62 | 3.05 | 1.61 | 3.06 | 1.61 | 5.44E-94 |
| *VAMP8* | 0.40 | -1.34 | 0.46 | -1.11 | 0.43 | -1.22 | 5.74E-94 |
| *KIAA0040* | 4.63 | 2.21 | 4.31 | 2.11 | 4.47 | 2.16 | 5.82E-94 |
| *ZNF684* | 2.41 | 1.27 | 2.35 | 1.23 | 2.38 | 1.25 | 9.36E-94 |
| *NUB1* | 3.14 | 1.65 | 2.98 | 1.58 | 3.06 | 1.61 | 1.07E-93 |
| *LST1* | 0.32 | -1.66 | 0.36 | -1.46 | 0.34 | -1.56 | 2.04E-93 |
| *AIF1* | 0.17 | -2.57 | 0.19 | -2.39 | 0.18 | -2.48 | 2.11E-93 |
| *GCOM1* | 0.16 | -2.68 | 0.18 | -2.49 | 0.17 | -2.59 | 2.12E-93 |
| *NCOA7* | 3.00 | 1.59 | 2.90 | 1.54 | 2.95 | 1.56 | 2.33E-93 |
| *WNT10B* | 0.37 | -1.42 | 0.41 | -1.27 | 0.39 | -1.34 | 2.72E-93 |
| *LOC100287559* | 3.11 | 1.64 | 2.56 | 1.35 | 2.82 | 1.50 | 2.87E-93 |
| *RAPGEF3* | 2.23 | 1.16 | 2.00 | 1.00 | 2.11 | 1.08 | 3.03E-93 |
| *CHODL* | 4.95 | 2.31 | 4.50 | 2.17 | 4.72 | 2.24 | 3.19E-93 |
| *RXRA* | 0.47 | -1.08 | 0.52 | -0.95 | 0.49 | -1.02 | 5.23E-93 |
| *CD33* | 0.07 | -3.93 | 0.13 | -2.97 | 0.09 | -3.45 | 6.35E-93 |
| *ABHD2* | 0.37 | -1.44 | 0.36 | -1.45 | 0.37 | -1.45 | 1.57E-92 |
| *C16orf74* | 0.26 | -1.96 | 0.28 | -1.85 | 0.27 | -1.90 | 2.25E-92 |
| *FAM101B* | 0.21 | -2.25 | 0.28 | -1.85 | 0.24 | -2.05 | 2.36E-92 |
| *ITGB2* | 0.22 | -2.20 | 0.25 | -2.00 | 0.23 | -2.10 | 4.07E-92 |
| *APBB1IP* | 0.48 | -1.06 | 0.51 | -0.96 | 0.50 | -1.01 | 7.82E-92 |
| *TMEM110-MUSTN1* | 2.16 | 1.11 | 1.98 | 0.98 | 2.07 | 1.05 | 1.04E-91 |
| *MSX2P1* | 0.33 | -1.61 | 0.34 | -1.55 | 0.33 | -1.58 | 1.39E-91 |
| *TRERF1* | 0.49 | -1.03 | 0.50 | -1.00 | 0.49 | -1.02 | 1.61E-91 |
| *GREM1* | 11.06 | 3.47 | 10.70 | 3.42 | 10.88 | 3.44 | 2.53E-91 |
| *FAM122C* | 2.79 | 1.48 | 2.54 | 1.35 | 2.66 | 1.41 | 2.59E-91 |
| *FCRLB* | 0.23 | -2.09 | 0.22 | -2.18 | 0.23 | -2.14 | 4.80E-91 |
| *LDHD* | 0.24 | -2.07 | 0.30 | -1.75 | 0.27 | -1.91 | 4.98E-91 |
| *PRKAR2A* | 0.47 | -1.07 | 0.52 | -0.95 | 0.50 | -1.01 | 9.08E-91 |
| *NLRC4* | 0.32 | -1.62 | 0.31 | -1.69 | 0.32 | -1.66 | 1.00E-90 |
| *LINGO3* | 0.36 | -1.47 | 0.37 | -1.43 | 0.37 | -1.45 | 1.20E-90 |
| *IER5L* | 0.29 | -1.78 | 0.33 | -1.61 | 0.31 | -1.70 | 2.21E-90 |
| *SRD5A3* | 0.36 | -1.47 | 0.41 | -1.29 | 0.38 | -1.38 | 6.61E-90 |
| *NOD1* | 2.32 | 1.22 | 2.27 | 1.18 | 2.30 | 1.20 | 1.11E-89 |
| *LOC643733* | 4.91 | 2.30 | 3.95 | 1.98 | 4.40 | 2.14 | 1.46E-88 |
| *ZNF107* | 2.17 | 1.12 | 2.06 | 1.04 | 2.11 | 1.08 | 1.58E-88 |
| *XRN1* | 3.61 | 1.85 | 3.38 | 1.76 | 3.49 | 1.80 | 1.71E-88 |
| *RSPH9* | 4.77 | 2.26 | 3.68 | 1.88 | 4.19 | 2.07 | 2.74E-88 |
| *IDO2* | 12.44 | 3.64 | 11.71 | 3.55 | 12.07 | 3.59 | 3.15E-88 |
| *HGF* | 0.13 | -2.96 | 0.13 | -2.89 | 0.13 | -2.93 | 3.39E-88 |
| *DLG4* | 0.42 | -1.24 | 0.46 | -1.12 | 0.44 | -1.18 | 4.03E-88 |
| *STOML1* | 2.85 | 1.51 | 3.02 | 1.59 | 2.93 | 1.55 | 4.70E-88 |
| *C20orf3* | 0.37 | -1.43 | 0.43 | -1.22 | 0.40 | -1.33 | 5.77E-88 |
| *CD1C* | 0.29 | -1.79 | 0.27 | -1.87 | 0.28 | -1.83 | 8.47E-88 |
| *KLRB1* | 0.36 | -1.47 | 0.39 | -1.36 | 0.37 | -1.42 | 1.95E-87 |
| *LHFPL1* | 5.40 | 2.43 | 6.55 | 2.71 | 5.94 | 2.57 | 3.29E-87 |
| *PRLR* | 21.60 | 4.43 | 16.80 | 4.07 | 19.05 | 4.25 | 6.84E-87 |
| *LOC286467* | 4.66 | 2.22 | 4.29 | 2.10 | 4.47 | 2.16 | 7.45E-87 |
| *PDCD1* | 3.44 | 1.78 | 2.59 | 1.37 | 2.99 | 1.58 | 1.26E-86 |
| *EPPK1* | 0.25 | -2.01 | 0.26 | -1.92 | 0.26 | -1.97 | 1.87E-86 |
| *TLR6* | 0.33 | -1.60 | 0.36 | -1.49 | 0.34 | -1.54 | 2.43E-86 |
| *TRIM26* | 2.52 | 1.33 | 2.39 | 1.26 | 2.45 | 1.29 | 3.36E-85 |
| *DAPP1* | 2.25 | 1.17 | 2.24 | 1.16 | 2.25 | 1.17 | 1.01E-84 |
| *MRPL44* | 2.09 | 1.06 | 1.93 | 0.95 | 2.01 | 1.01 | 1.20E-84 |
| *EIF4EBP1* | 0.35 | -1.53 | 0.40 | -1.33 | 0.37 | -1.43 | 1.29E-84 |
| *LSS* | 2.14 | 1.10 | 2.18 | 1.12 | 2.16 | 1.11 | 3.00E-84 |
| *CATSPER1* | 0.23 | -2.10 | 0.28 | -1.86 | 0.25 | -1.98 | 3.31E-84 |
| *PLXDC1* | 0.27 | -1.87 | 0.30 | -1.72 | 0.29 | -1.79 | 3.72E-84 |
| *HSD17B4* | 0.17 | -2.54 | 0.20 | -2.34 | 0.18 | -2.44 | 5.01E-84 |
| *NEFL* | 0.16 | -2.67 | 0.20 | -2.31 | 0.18 | -2.49 | 5.25E-84 |
| *ME1* | 0.24 | -2.05 | 0.31 | -1.70 | 0.27 | -1.88 | 5.87E-84 |
| *SH3BGRL* | 0.46 | -1.12 | 0.47 | -1.08 | 0.47 | -1.10 | 1.41E-83 |
| *DDIT3* | 2.39 | 1.26 | 2.06 | 1.04 | 2.22 | 1.15 | 1.91E-83 |
| *UCP2* | 0.41 | -1.27 | 0.44 | -1.19 | 0.43 | -1.23 | 4.76E-83 |
| *EDARADD* | 6.31 | 2.66 | 4.64 | 2.21 | 5.41 | 2.44 | 5.67E-83 |
| *HAAO* | 0.31 | -1.69 | 0.33 | -1.59 | 0.32 | -1.64 | 8.70E-83 |
| *SLC38A5* | 7.82 | 2.97 | 7.02 | 2.81 | 7.41 | 2.89 | 1.00E-82 |
| *TNFSF4* | 2.47 | 1.30 | 2.13 | 1.09 | 2.29 | 1.20 | 1.08E-82 |
| *HOPX* | 0.34 | -1.56 | 0.35 | -1.52 | 0.34 | -1.54 | 1.51E-82 |
| *CASZ1* | 3.24 | 1.70 | 2.63 | 1.39 | 2.92 | 1.54 | 2.84E-82 |
| *CENPJ* | 2.44 | 1.28 | 2.23 | 1.16 | 2.33 | 1.22 | 2.86E-82 |
| *ZNF620* | 2.37 | 1.25 | 2.15 | 1.10 | 2.26 | 1.17 | 3.63E-82 |
| *VASH1* | 0.26 | -1.96 | 0.26 | -1.92 | 0.26 | -1.94 | 7.74E-82 |
| *CHST12* | 3.68 | 1.88 | 3.17 | 1.66 | 3.42 | 1.77 | 1.03E-81 |
| *FES* | 0.26 | -1.94 | 0.32 | -1.65 | 0.29 | -1.80 | 1.08E-81 |
| *EIF4E3* | 2.39 | 1.26 | 2.25 | 1.17 | 2.32 | 1.21 | 1.16E-81 |
| *PRR24* | 2.35 | 1.23 | 2.38 | 1.25 | 2.37 | 1.24 | 1.36E-81 |
| *OLFM1* | 0.08 | -3.62 | 0.14 | -2.81 | 0.11 | -3.21 | 1.40E-81 |
| *FAM123B* | 2.10 | 1.07 | 2.07 | 1.05 | 2.09 | 1.06 | 1.63E-81 |
| *HHEX* | 0.38 | -1.39 | 0.38 | -1.41 | 0.38 | -1.40 | 1.66E-81 |
| *FXYD7* | 0.33 | -1.61 | 0.38 | -1.39 | 0.35 | -1.50 | 2.04E-81 |
| *DNAAF1* | 22.58 | 4.50 | 22.24 | 4.48 | 22.41 | 4.49 | 5.22E-81 |
| *REPS2* | 0.44 | -1.18 | 0.50 | -1.01 | 0.47 | -1.09 | 5.85E-81 |
| *TTC3* | 0.45 | -1.16 | 0.50 | -1.00 | 0.47 | -1.08 | 1.64E-80 |
| *TMEM14C* | 0.43 | -1.21 | 0.51 | -0.97 | 0.47 | -1.09 | 1.65E-80 |
| *WNT7A* | 0.24 | -2.07 | 0.28 | -1.85 | 0.26 | -1.96 | 1.98E-80 |
| *ENDOD1* | 4.42 | 2.14 | 4.07 | 2.03 | 4.24 | 2.08 | 2.38E-80 |
| *CPEB2* | 3.60 | 1.85 | 3.18 | 1.67 | 3.39 | 1.76 | 2.83E-80 |
| *RPL4* | 0.48 | -1.06 | 0.52 | -0.96 | 0.50 | -1.01 | 2.83E-80 |
| *LOC100129034* | 0.34 | -1.56 | 0.33 | -1.58 | 0.34 | -1.57 | 3.04E-80 |
| *TGFBR1* | 0.26 | -1.95 | 0.30 | -1.76 | 0.28 | -1.86 | 3.89E-80 |
| *C12orf57* | 0.33 | -1.60 | 0.37 | -1.42 | 0.35 | -1.51 | 6.81E-80 |
| *MAPK8IP2* | 8.47 | 3.08 | 6.68 | 2.74 | 7.52 | 2.91 | 7.35E-80 |
| *KCNC3* | 0.47 | -1.08 | 0.48 | -1.05 | 0.48 | -1.07 | 7.55E-80 |
| *FUCA1* | 0.29 | -1.77 | 0.30 | -1.76 | 0.29 | -1.76 | 8.51E-80 |
| *PI16* | 0.14 | -2.80 | 0.22 | -2.17 | 0.18 | -2.48 | 9.18E-80 |
| *NKX3-1* | 2.94 | 1.56 | 2.70 | 1.43 | 2.81 | 1.49 | 1.46E-79 |
| *NCAM1* | 2.99 | 1.58 | 2.61 | 1.38 | 2.79 | 1.48 | 1.51E-79 |
| *C17orf67* | 3.57 | 1.83 | 3.04 | 1.60 | 3.29 | 1.72 | 1.71E-79 |
| *ACOT9* | 3.42 | 1.77 | 3.15 | 1.66 | 3.28 | 1.71 | 2.03E-79 |
| *CLEC11A* | 0.29 | -1.78 | 0.34 | -1.58 | 0.31 | -1.68 | 2.27E-79 |
| *C9orf139* | 0.40 | -1.34 | 0.39 | -1.35 | 0.39 | -1.34 | 3.87E-79 |
| *RAPGEF2* | 2.89 | 1.53 | 2.76 | 1.46 | 2.82 | 1.50 | 3.95E-79 |
| *RGS14* | 0.40 | -1.32 | 0.44 | -1.19 | 0.42 | -1.26 | 4.56E-79 |
| *KLF6* | 3.45 | 1.79 | 2.93 | 1.55 | 3.18 | 1.67 | 5.87E-79 |
| *IL15* | 4.76 | 2.25 | 3.94 | 1.98 | 4.33 | 2.12 | 7.10E-79 |
| *GAPT* | 0.13 | -2.95 | 0.16 | -2.66 | 0.14 | -2.81 | 8.37E-79 |
| *CCRL2* | 4.93 | 2.30 | 3.48 | 1.80 | 4.14 | 2.05 | 4.00E-78 |
| *C4orf36* | 4.88 | 2.29 | 3.55 | 1.83 | 4.16 | 2.06 | 5.66E-78 |
| *ITIH4* | 2.53 | 1.34 | 2.38 | 1.25 | 2.45 | 1.30 | 1.03E-77 |
| *ALDH2* | 0.21 | -2.28 | 0.23 | -2.11 | 0.22 | -2.20 | 1.32E-77 |
| *CPEB3* | 2.78 | 1.47 | 2.68 | 1.42 | 2.73 | 1.45 | 1.91E-77 |
| *SPINT2* | 0.45 | -1.16 | 0.51 | -0.97 | 0.48 | -1.06 | 2.62E-77 |
| *RNF125* | 0.38 | -1.38 | 0.44 | -1.18 | 0.41 | -1.28 | 3.82E-77 |
| *CBLN3* | 3.50 | 1.81 | 3.07 | 1.62 | 3.27 | 1.71 | 4.20E-77 |
| *TRIP6* | 3.49 | 1.80 | 3.26 | 1.70 | 3.37 | 1.75 | 5.31E-77 |
| *GTPBP1* | 3.40 | 1.77 | 3.01 | 1.59 | 3.20 | 1.68 | 6.92E-77 |
| *LRRN2* | 3.78 | 1.92 | 3.23 | 1.69 | 3.50 | 1.81 | 7.43E-77 |
| *TMEM45B* | 0.39 | -1.34 | 0.36 | -1.48 | 0.38 | -1.41 | 9.60E-77 |
| *PKDREJ* | 0.32 | -1.66 | 0.34 | -1.57 | 0.33 | -1.61 | 1.01E-76 |
| *VWF* | 0.19 | -2.39 | 0.16 | -2.60 | 0.18 | -2.50 | 1.03E-76 |
| *ADAM22* | 0.47 | -1.09 | 0.51 | -0.97 | 0.49 | -1.03 | 1.04E-76 |
| *NCRNA00086* | 0.30 | -1.75 | 0.32 | -1.63 | 0.31 | -1.69 | 1.18E-76 |
| *LAG3* | 10.54 | 3.40 | 9.14 | 3.19 | 9.82 | 3.30 | 1.47E-76 |
| *RORC* | 0.39 | -1.35 | 0.44 | -1.19 | 0.42 | -1.27 | 1.53E-76 |
| *ABCD1* | 4.94 | 2.31 | 3.98 | 1.99 | 4.44 | 2.15 | 1.69E-76 |
| *KPTN* | 2.08 | 1.06 | 2.06 | 1.05 | 2.07 | 1.05 | 3.02E-76 |
| *SLCO2B1* | 0.13 | -2.91 | 0.15 | -2.70 | 0.14 | -2.80 | 3.53E-76 |
| *TRIM69* | 5.08 | 2.34 | 3.51 | 1.81 | 4.22 | 2.08 | 7.20E-76 |
| *SRD5A1* | 3.58 | 1.84 | 2.84 | 1.51 | 3.19 | 1.67 | 7.64E-76 |
| *APBA1* | 0.09 | -3.48 | 0.15 | -2.77 | 0.11 | -3.13 | 1.24E-75 |
| *DNM1* | 0.31 | -1.67 | 0.32 | -1.63 | 0.32 | -1.65 | 1.42E-75 |
| *BUB1* | 3.29 | 1.72 | 2.87 | 1.52 | 3.07 | 1.62 | 1.61E-75 |
| *CD38* | 20.50 | 4.36 | 18.15 | 4.18 | 19.29 | 4.27 | 1.65E-75 |
| *C1orf170* | 5.42 | 2.44 | 4.47 | 2.16 | 4.92 | 2.30 | 1.76E-75 |
| *SCML4* | 2.25 | 1.17 | 2.01 | 1.01 | 2.13 | 1.09 | 2.45E-75 |
| *GBP3* | 3.91 | 1.97 | 3.76 | 1.91 | 3.83 | 1.94 | 2.66E-75 |
| *EMR4P* | 0.22 | -2.20 | 0.24 | -2.08 | 0.23 | -2.14 | 2.76E-75 |
| *PDZD4* | 0.37 | -1.45 | 0.41 | -1.29 | 0.39 | -1.37 | 2.79E-75 |
| *GBP4* | 6.48 | 2.70 | 6.03 | 2.59 | 6.25 | 2.64 | 3.65E-75 |
| *PLEKHN1* | 5.40 | 2.43 | 4.42 | 2.15 | 4.89 | 2.29 | 5.27E-75 |
| *KIAA1217* | 4.97 | 2.31 | 4.03 | 2.01 | 4.47 | 2.16 | 6.61E-75 |
| *MSR1* | 10.95 | 3.45 | 9.16 | 3.20 | 10.01 | 3.32 | 7.24E-75 |
| *IL15RA* | 10.25 | 3.36 | 8.53 | 3.09 | 9.35 | 3.23 | 1.20E-74 |
| *BRI3BP* | 0.40 | -1.32 | 0.44 | -1.19 | 0.42 | -1.26 | 1.38E-74 |
| *ITGAM* | 0.21 | -2.26 | 0.23 | -2.10 | 0.22 | -2.18 | 1.42E-74 |
| *GRAMD4* | 0.17 | -2.55 | 0.22 | -2.19 | 0.19 | -2.37 | 1.45E-74 |
| *POU5F1B* | 2.68 | 1.42 | 2.54 | 1.35 | 2.61 | 1.38 | 1.78E-74 |
| *HIRA* | 2.11 | 1.07 | 2.05 | 1.04 | 2.08 | 1.06 | 3.17E-74 |
| *AIM2* | 8.84 | 3.14 | 7.46 | 2.90 | 8.12 | 3.02 | 3.77E-74 |
| *9-Sep* | 0.46 | -1.13 | 0.49 | -1.03 | 0.47 | -1.08 | 5.21E-74 |
| *ARID5B* | 2.22 | 1.15 | 2.05 | 1.04 | 2.13 | 1.09 | 5.31E-74 |
| *PPP2R2A* | 2.29 | 1.19 | 2.02 | 1.01 | 2.15 | 1.10 | 7.08E-74 |
| *CHAC1* | 5.02 | 2.33 | 3.94 | 1.98 | 4.44 | 2.15 | 8.61E-74 |
| *ABHD16B* | 2.35 | 1.23 | 1.97 | 0.98 | 2.15 | 1.11 | 1.19E-73 |
| *CASP4* | 2.52 | 1.33 | 2.20 | 1.14 | 2.35 | 1.24 | 1.49E-73 |
| *TM6SF1* | 0.18 | -2.47 | 0.20 | -2.30 | 0.19 | -2.38 | 1.51E-73 |
| *PRSS36* | 0.35 | -1.49 | 0.36 | -1.49 | 0.36 | -1.49 | 2.39E-73 |
| *PDCD1LG2* | 6.63 | 2.73 | 5.84 | 2.55 | 6.22 | 2.64 | 3.15E-73 |
| *ESCO2* | 0.34 | -1.54 | 0.42 | -1.26 | 0.38 | -1.40 | 3.44E-73 |
| *HIP1* | 0.34 | -1.56 | 0.41 | -1.28 | 0.37 | -1.42 | 8.00E-73 |
| *C3orf52* | 2.84 | 1.51 | 3.00 | 1.59 | 2.92 | 1.55 | 8.10E-73 |
| *NUDT9P1* | 2.32 | 1.21 | 2.21 | 1.15 | 2.27 | 1.18 | 9.40E-73 |
| *TMEM187* | 2.69 | 1.43 | 2.43 | 1.28 | 2.56 | 1.36 | 2.06E-72 |
| *SRGAP2P2* | 4.81 | 2.26 | 4.12 | 2.04 | 4.45 | 2.15 | 2.17E-72 |
| *TNNI2* | 0.36 | -1.46 | 0.35 | -1.50 | 0.36 | -1.48 | 2.49E-72 |
| *CD300LF* | 0.19 | -2.43 | 0.23 | -2.15 | 0.20 | -2.29 | 3.03E-72 |
| *LIPA* | 0.27 | -1.87 | 0.26 | -1.92 | 0.27 | -1.90 | 3.13E-72 |
| *CD69* | 3.42 | 1.77 | 2.68 | 1.42 | 3.03 | 1.60 | 3.15E-72 |
| *MAML3* | 0.20 | -2.35 | 0.27 | -1.89 | 0.23 | -2.12 | 3.96E-72 |
| *CARD9* | 0.21 | -2.28 | 0.24 | -2.08 | 0.22 | -2.18 | 4.53E-72 |
| *LOC100506033* | 2.15 | 1.11 | 1.93 | 0.95 | 2.04 | 1.03 | 6.56E-72 |
| *CASP10* | 2.23 | 1.16 | 2.21 | 1.15 | 2.22 | 1.15 | 9.05E-72 |
| *TMEM71* | 0.36 | -1.46 | 0.40 | -1.30 | 0.38 | -1.38 | 9.32E-72 |
| *HLA-L* | 2.47 | 1.30 | 2.13 | 1.09 | 2.29 | 1.20 | 9.33E-72 |
| *DFNB31* | 3.51 | 1.81 | 2.78 | 1.48 | 3.12 | 1.64 | 1.03E-71 |
| *FAM109A* | 0.40 | -1.31 | 0.44 | -1.19 | 0.42 | -1.25 | 2.47E-71 |
| *MAML2* | 2.08 | 1.05 | 1.94 | 0.96 | 2.01 | 1.00 | 2.54E-71 |
| *IRF4* | 2.15 | 1.10 | 1.97 | 0.98 | 2.05 | 1.04 | 2.68E-71 |
| *TRAF2* | 2.19 | 1.13 | 2.03 | 1.02 | 2.11 | 1.08 | 3.33E-71 |
| *ESYT1* | 0.44 | -1.17 | 0.48 | -1.07 | 0.46 | -1.12 | 3.89E-71 |
| *PLSCR4* | 5.49 | 2.46 | 6.74 | 2.75 | 6.08 | 2.60 | 4.68E-71 |
| *BAZ1A* | 2.09 | 1.06 | 2.00 | 1.00 | 2.05 | 1.03 | 5.70E-71 |
| *CREBL2* | 0.43 | -1.21 | 0.51 | -0.98 | 0.47 | -1.09 | 2.09E-70 |
| *LOC100129550* | 0.37 | -1.43 | 0.39 | -1.35 | 0.38 | -1.39 | 3.21E-70 |
| *HMMR* | 2.12 | 1.09 | 1.92 | 0.94 | 2.02 | 1.01 | 4.58E-70 |
| *SLC37A2* | 0.28 | -1.82 | 0.29 | -1.77 | 0.29 | -1.80 | 5.33E-70 |
| *TMEM86A* | 0.36 | -1.48 | 0.38 | -1.39 | 0.37 | -1.43 | 6.89E-70 |
| *ANK3* | 2.05 | 1.04 | 2.00 | 1.00 | 2.03 | 1.02 | 7.34E-70 |
| *SLC27A2* | 2.41 | 1.27 | 2.06 | 1.04 | 2.22 | 1.15 | 9.51E-70 |
| *RAB3A* | 0.41 | -1.28 | 0.47 | -1.09 | 0.44 | -1.18 | 1.07E-69 |
| *FAT4* | 0.46 | -1.12 | 0.49 | -1.04 | 0.47 | -1.08 | 1.46E-69 |
| *STAB1* | 0.15 | -2.78 | 0.13 | -2.90 | 0.14 | -2.84 | 1.48E-69 |
| *FCRL6* | 0.42 | -1.25 | 0.40 | -1.31 | 0.41 | -1.28 | 1.52E-69 |
| *OXER1* | 0.29 | -1.80 | 0.30 | -1.76 | 0.29 | -1.78 | 1.56E-69 |
| *PRAM1* | 0.15 | -2.77 | 0.13 | -2.93 | 0.14 | -2.85 | 2.22E-69 |
| *BLVRB* | 0.27 | -1.87 | 0.36 | -1.46 | 0.31 | -1.67 | 3.78E-69 |
| *C1orf93* | 0.46 | -1.12 | 0.49 | -1.03 | 0.47 | -1.08 | 4.71E-69 |
| *KLF2* | 0.43 | -1.20 | 0.45 | -1.15 | 0.44 | -1.18 | 5.83E-69 |
| *RPL22* | 0.47 | -1.10 | 0.49 | -1.02 | 0.48 | -1.06 | 6.04E-69 |
| *MRC2* | 0.23 | -2.11 | 0.29 | -1.77 | 0.26 | -1.94 | 6.37E-69 |
| *PSTPIP1* | 0.50 | -1.00 | 0.47 | -1.08 | 0.49 | -1.04 | 8.34E-69 |
| *SP140* | 3.55 | 1.83 | 3.15 | 1.66 | 3.34 | 1.74 | 1.02E-68 |
| *SPIRE1* | 0.36 | -1.46 | 0.45 | -1.14 | 0.40 | -1.30 | 1.02E-68 |
| *JAK2* | 3.77 | 1.91 | 3.70 | 1.89 | 3.74 | 1.90 | 1.09E-68 |
| *CCL22* | 0.36 | -1.48 | 0.34 | -1.57 | 0.35 | -1.52 | 1.63E-68 |
| *MICB* | 2.89 | 1.53 | 2.55 | 1.35 | 2.72 | 1.44 | 1.71E-68 |
| *DPCD* | 0.42 | -1.24 | 0.41 | -1.28 | 0.42 | -1.26 | 2.05E-68 |
| *CD83* | 3.36 | 1.75 | 2.89 | 1.53 | 3.12 | 1.64 | 3.65E-68 |
| *RFPL3-AS1* | 2.67 | 1.41 | 2.16 | 1.11 | 2.40 | 1.26 | 4.25E-68 |
| *PKI55* | 0.47 | -1.09 | 0.47 | -1.07 | 0.47 | -1.08 | 4.72E-68 |
| *YEATS2* | 2.84 | 1.51 | 2.66 | 1.41 | 2.75 | 1.46 | 5.80E-68 |
| *CALU* | 0.35 | -1.53 | 0.41 | -1.27 | 0.38 | -1.40 | 6.34E-68 |
| *CMYA5* | 2.59 | 1.37 | 2.34 | 1.22 | 2.46 | 1.30 | 6.81E-68 |
| *LOC653786* | 0.11 | -3.20 | 0.14 | -2.86 | 0.12 | -3.03 | 7.82E-68 |
| *MCOLN2* | 4.49 | 2.17 | 4.53 | 2.18 | 4.51 | 2.17 | 9.47E-68 |
| *TMEM107* | 0.46 | -1.13 | 0.48 | -1.05 | 0.47 | -1.09 | 1.08E-67 |
| *ARID5A* | 2.32 | 1.21 | 2.04 | 1.03 | 2.17 | 1.12 | 1.20E-67 |
| *PVRL3* | 2.82 | 1.50 | 2.34 | 1.23 | 2.57 | 1.36 | 1.27E-67 |
| *HAL* | 0.18 | -2.49 | 0.19 | -2.43 | 0.18 | -2.46 | 1.28E-67 |
| *NOG* | 0.44 | -1.19 | 0.46 | -1.11 | 0.45 | -1.15 | 1.62E-67 |
| *CYFIP1* | 0.36 | -1.49 | 0.38 | -1.38 | 0.37 | -1.43 | 1.67E-67 |
| *CD52* | 0.38 | -1.41 | 0.36 | -1.47 | 0.37 | -1.44 | 2.43E-67 |
| *FGD4* | 0.32 | -1.66 | 0.32 | -1.65 | 0.32 | -1.66 | 2.44E-67 |
| *KIAA0226* | 2.50 | 1.32 | 2.29 | 1.19 | 2.39 | 1.26 | 2.54E-67 |
| *FLJ23867* | 0.43 | -1.22 | 0.44 | -1.19 | 0.43 | -1.21 | 2.57E-67 |
| *BLZF1* | 3.86 | 1.95 | 3.14 | 1.65 | 3.48 | 1.80 | 2.66E-67 |
| *MLEC* | 0.47 | -1.08 | 0.50 | -0.99 | 0.49 | -1.04 | 3.54E-67 |
| *CTSA* | 0.35 | -1.50 | 0.44 | -1.18 | 0.40 | -1.34 | 4.22E-67 |
| *CHN2* | 0.43 | -1.22 | 0.49 | -1.04 | 0.46 | -1.13 | 4.57E-67 |
| *ANKRD58* | 0.32 | -1.63 | 0.32 | -1.63 | 0.32 | -1.63 | 6.10E-67 |
| *NINJ2* | 0.40 | -1.32 | 0.49 | -1.02 | 0.44 | -1.17 | 8.16E-67 |
| *TFEC* | 2.28 | 1.19 | 2.16 | 1.11 | 2.22 | 1.15 | 9.47E-67 |
| *EVI2B* | 0.45 | -1.16 | 0.45 | -1.14 | 0.45 | -1.15 | 1.14E-66 |
| *LOC653653* | 0.38 | -1.38 | 0.43 | -1.23 | 0.41 | -1.30 | 1.26E-66 |
| *RUNX2* | 2.65 | 1.41 | 2.41 | 1.27 | 2.53 | 1.34 | 1.35E-66 |
| *FAM125B* | 3.27 | 1.71 | 3.15 | 1.66 | 3.21 | 1.68 | 1.80E-66 |
| *PLLP* | 0.24 | -2.05 | 0.28 | -1.82 | 0.26 | -1.94 | 2.34E-66 |
| *C14orf132* | 0.31 | -1.69 | 0.35 | -1.52 | 0.33 | -1.61 | 2.88E-66 |
| *CTTNBP2* | 0.08 | -3.61 | 0.07 | -3.90 | 0.07 | -3.75 | 3.13E-66 |
| *TMEM139* | 4.25 | 2.09 | 3.54 | 1.82 | 3.88 | 1.96 | 3.18E-66 |
| *GLB1L* | 0.46 | -1.13 | 0.51 | -0.98 | 0.48 | -1.06 | 4.73E-66 |
| *CLEC4A* | 0.21 | -2.22 | 0.27 | -1.90 | 0.24 | -2.06 | 5.03E-66 |
| *GPR15* | 2.40 | 1.27 | 2.13 | 1.09 | 2.26 | 1.18 | 5.18E-66 |
| *GSTA4* | 0.43 | -1.23 | 0.46 | -1.11 | 0.44 | -1.17 | 5.38E-66 |
| *C9orf91* | 3.40 | 1.76 | 3.38 | 1.76 | 3.39 | 1.76 | 6.59E-66 |
| *GALNT8* | 2.37 | 1.24 | 2.34 | 1.22 | 2.35 | 1.23 | 8.31E-66 |
| *MGST2* | 0.27 | -1.91 | 0.35 | -1.54 | 0.30 | -1.72 | 8.43E-66 |
| *HCG4* | 2.37 | 1.25 | 2.36 | 1.24 | 2.37 | 1.24 | 1.26E-65 |
| *CASP1* | 2.22 | 1.15 | 2.27 | 1.18 | 2.25 | 1.17 | 1.33E-65 |
| *TPX2* | 2.68 | 1.42 | 2.45 | 1.29 | 2.56 | 1.36 | 1.60E-65 |
| *TRAFD1* | 4.36 | 2.13 | 4.15 | 2.05 | 4.26 | 2.09 | 1.63E-65 |
| *RAB19* | 2.44 | 1.29 | 2.13 | 1.09 | 2.28 | 1.19 | 2.33E-65 |
| *BAK1* | 2.25 | 1.17 | 2.11 | 1.08 | 2.18 | 1.12 | 2.43E-65 |
| *GAS7* | 0.45 | -1.16 | 0.43 | -1.20 | 0.44 | -1.18 | 2.66E-65 |
| *GRIP1* | 3.05 | 1.61 | 2.55 | 1.35 | 2.79 | 1.48 | 4.53E-65 |
| *CHI3L2* | 2.87 | 1.52 | 2.65 | 1.41 | 2.76 | 1.46 | 6.59E-65 |
| *SPATA6* | 0.38 | -1.40 | 0.42 | -1.25 | 0.40 | -1.33 | 7.32E-65 |
| *ZNF462* | 2.34 | 1.23 | 2.47 | 1.30 | 2.40 | 1.27 | 7.97E-65 |
| *C5* | 2.39 | 1.26 | 2.39 | 1.26 | 2.39 | 1.26 | 8.23E-65 |
| *BRIP1* | 5.47 | 2.45 | 4.50 | 2.17 | 4.96 | 2.31 | 8.41E-65 |
| *LOC100506585* | 0.05 | -4.37 | 0.06 | -4.07 | 0.05 | -4.22 | 1.02E-64 |
| *CARD17* | 8.60 | 3.10 | 9.37 | 3.23 | 8.98 | 3.17 | 1.03E-64 |
| *C5orf56* | 3.52 | 1.81 | 2.93 | 1.55 | 3.21 | 1.68 | 1.08E-64 |
| *HTRA1* | 0.07 | -3.91 | 0.08 | -3.69 | 0.07 | -3.80 | 1.10E-64 |
| *GPR141* | 2.88 | 1.53 | 2.83 | 1.50 | 2.86 | 1.51 | 1.21E-64 |
| *ODZ1* | 0.48 | -1.06 | 0.49 | -1.04 | 0.48 | -1.05 | 1.37E-64 |
| *GPR172B* | 3.00 | 1.58 | 2.63 | 1.39 | 2.81 | 1.49 | 1.94E-64 |
| *FOXRED2* | 0.35 | -1.52 | 0.44 | -1.20 | 0.39 | -1.36 | 2.38E-64 |
| *ADK* | 0.35 | -1.51 | 0.39 | -1.35 | 0.37 | -1.43 | 4.05E-64 |
| *SEPP1* | 0.02 | -5.74 | 0.03 | -5.10 | 0.02 | -5.42 | 4.71E-64 |
| *STOM* | 2.05 | 1.04 | 2.27 | 1.18 | 2.16 | 1.11 | 5.72E-64 |
| *ZNF267* | 2.56 | 1.36 | 2.08 | 1.06 | 2.31 | 1.21 | 5.92E-64 |
| *MIR155HG* | 4.03 | 2.01 | 3.01 | 1.59 | 3.48 | 1.80 | 7.85E-64 |
| *RGS18* | 0.13 | -2.90 | 0.18 | -2.48 | 0.16 | -2.69 | 8.46E-64 |
| *AP2S1* | 0.47 | -1.08 | 0.52 | -0.95 | 0.49 | -1.02 | 1.03E-63 |
| *NCRNA00173* | 2.95 | 1.56 | 2.91 | 1.54 | 2.93 | 1.55 | 1.16E-63 |
| *GCNT2* | 2.40 | 1.26 | 2.33 | 1.22 | 2.36 | 1.24 | 1.22E-63 |
| *ST8SIA4* | 2.34 | 1.22 | 2.24 | 1.16 | 2.29 | 1.19 | 1.40E-63 |
| *NR4A3* | 5.76 | 2.52 | 4.47 | 2.16 | 5.07 | 2.34 | 2.16E-63 |
| *C6orf191* | 3.33 | 1.73 | 2.99 | 1.58 | 3.15 | 1.66 | 3.08E-63 |
| *SRGAP2* | 4.80 | 2.26 | 4.16 | 2.06 | 4.47 | 2.16 | 4.05E-63 |
| *NOD2* | 3.21 | 1.68 | 2.82 | 1.49 | 3.01 | 1.59 | 5.88E-63 |
| *MYO1G* | 2.65 | 1.41 | 2.39 | 1.25 | 2.51 | 1.33 | 6.24E-63 |
| *C5orf20* | 0.24 | -2.07 | 0.28 | -1.84 | 0.26 | -1.95 | 8.49E-63 |
| *BAALC* | 7.48 | 2.90 | 5.13 | 2.36 | 6.19 | 2.63 | 1.05E-62 |
| *NAA25* | 3.06 | 1.61 | 2.65 | 1.40 | 2.84 | 1.51 | 1.71E-62 |
| *KLHL14* | 0.46 | -1.12 | 0.50 | -1.01 | 0.48 | -1.06 | 1.92E-62 |
| *AMOTL2* | 27.33 | 4.77 | 20.05 | 4.33 | 23.41 | 4.55 | 2.24E-62 |
| *C8orf56* | 29.32 | 4.87 | 12.63 | 3.66 | 19.23 | 4.27 | 2.24E-62 |
| *MYCL1* | 0.28 | -1.84 | 0.29 | -1.78 | 0.28 | -1.81 | 2.62E-62 |
| *SH3RF3* | 0.45 | -1.15 | 0.50 | -1.01 | 0.47 | -1.08 | 2.70E-62 |
| *CFLAR-AS1* | 2.32 | 1.22 | 1.99 | 1.00 | 2.15 | 1.11 | 3.05E-62 |
| *STK32C* | 0.47 | -1.10 | 0.53 | -0.91 | 0.50 | -1.00 | 3.19E-62 |
| *B3GNT2* | 2.84 | 1.51 | 2.33 | 1.22 | 2.57 | 1.36 | 3.58E-62 |
| *FANCA* | 2.97 | 1.57 | 2.63 | 1.39 | 2.79 | 1.48 | 5.10E-62 |
| *SLC46A3* | 0.35 | -1.50 | 0.43 | -1.22 | 0.39 | -1.36 | 5.13E-62 |
| *PPP1R15A* | 2.52 | 1.33 | 2.04 | 1.03 | 2.27 | 1.18 | 5.25E-62 |
| *NHS* | 0.06 | -4.11 | 0.09 | -3.48 | 0.07 | -3.80 | 5.69E-62 |
| *GCH1* | 8.98 | 3.17 | 7.29 | 2.87 | 8.09 | 3.02 | 7.03E-62 |
| *TMEM62* | 3.60 | 1.85 | 2.99 | 1.58 | 3.28 | 1.71 | 7.93E-62 |
| *ADAM15* | 0.39 | -1.37 | 0.43 | -1.22 | 0.41 | -1.29 | 9.74E-62 |
| *ZBTB42* | 2.47 | 1.30 | 2.44 | 1.29 | 2.46 | 1.30 | 1.19E-61 |
| *TSPAN18* | 0.29 | -1.78 | 0.37 | -1.45 | 0.33 | -1.61 | 1.41E-61 |
| *ATF3* | 15.68 | 3.97 | 12.15 | 3.60 | 13.80 | 3.79 | 1.56E-61 |
| *LOC100507421* | 9.37 | 3.23 | 8.27 | 3.05 | 8.80 | 3.14 | 1.72E-61 |
| *BAG1* | 2.35 | 1.23 | 2.22 | 1.15 | 2.29 | 1.19 | 2.92E-61 |
| *SLC27A1* | 0.26 | -1.95 | 0.29 | -1.78 | 0.27 | -1.87 | 3.82E-61 |
| *C15orf38* | 0.45 | -1.14 | 0.50 | -1.00 | 0.48 | -1.07 | 4.40E-61 |
| *CSRP2* | 10.30 | 3.36 | 6.80 | 2.76 | 8.37 | 3.06 | 4.96E-61 |
| *ECE1* | 3.69 | 1.88 | 3.24 | 1.69 | 3.45 | 1.79 | 6.56E-61 |
| *TAP1* | 3.58 | 1.84 | 3.30 | 1.72 | 3.44 | 1.78 | 8.95E-61 |
| *ICAM4* | 0.33 | -1.58 | 0.35 | -1.52 | 0.34 | -1.55 | 9.19E-61 |
| *RBL2* | 0.43 | -1.21 | 0.45 | -1.15 | 0.44 | -1.18 | 1.47E-60 |
| *PROS1* | 0.21 | -2.26 | 0.21 | -2.23 | 0.21 | -2.24 | 1.50E-60 |
| *ARHGAP6* | 0.18 | -2.44 | 0.21 | -2.28 | 0.19 | -2.36 | 1.57E-60 |
| *EIF3F* | 0.48 | -1.05 | 0.51 | -0.97 | 0.50 | -1.01 | 1.69E-60 |
| *RBM11* | 3.15 | 1.65 | 2.85 | 1.51 | 3.00 | 1.58 | 2.19E-60 |
| *TTC39B* | 2.80 | 1.49 | 2.46 | 1.30 | 2.62 | 1.39 | 2.53E-60 |
| *CKB* | 12.56 | 3.65 | 8.99 | 3.17 | 10.63 | 3.41 | 2.79E-60 |
| *ERP27* | 0.41 | -1.29 | 0.42 | -1.26 | 0.41 | -1.28 | 2.79E-60 |
| *DOCK3* | 0.45 | -1.15 | 0.48 | -1.06 | 0.47 | -1.10 | 3.01E-60 |
| *CAT* | 0.29 | -1.79 | 0.31 | -1.69 | 0.30 | -1.74 | 4.21E-60 |
| *FAM115C* | 2.14 | 1.10 | 1.89 | 0.92 | 2.01 | 1.01 | 4.69E-60 |
| *LGALS3* | 0.48 | -1.06 | 0.48 | -1.04 | 0.48 | -1.05 | 5.19E-60 |
| *ARL5B* | 2.74 | 1.45 | 2.53 | 1.34 | 2.63 | 1.40 | 6.27E-60 |
| *PLEKHA4* | 3.30 | 1.72 | 2.68 | 1.42 | 2.97 | 1.57 | 7.42E-60 |
| *NINJ1* | 2.83 | 1.50 | 2.50 | 1.32 | 2.66 | 1.41 | 8.70E-60 |
| *WDR17* | 0.49 | -1.03 | 0.51 | -0.98 | 0.50 | -1.01 | 8.80E-60 |
| *EIF4B* | 0.45 | -1.16 | 0.47 | -1.08 | 0.46 | -1.12 | 1.23E-59 |
| *SIGLEC9* | 0.33 | -1.61 | 0.41 | -1.30 | 0.37 | -1.45 | 2.91E-59 |
| *VWA5B1* | 3.09 | 1.63 | 2.62 | 1.39 | 2.85 | 1.51 | 3.39E-59 |
| *GZMA* | 0.39 | -1.37 | 0.40 | -1.34 | 0.39 | -1.35 | 4.51E-59 |
| *FADS1* | 0.27 | -1.90 | 0.32 | -1.63 | 0.29 | -1.77 | 5.85E-59 |
| *PKIB* | 0.17 | -2.57 | 0.22 | -2.17 | 0.19 | -2.37 | 7.67E-59 |
| *CCDC9* | 2.04 | 1.03 | 2.03 | 1.02 | 2.03 | 1.02 | 8.41E-59 |
| *ARHGEF11* | 2.78 | 1.47 | 2.60 | 1.38 | 2.68 | 1.42 | 8.87E-59 |
| *SORL1* | 0.28 | -1.83 | 0.33 | -1.60 | 0.30 | -1.72 | 8.90E-59 |
| *DNER* | 0.25 | -2.03 | 0.31 | -1.70 | 0.29 | -1.77 | 1.05E-58 |
| *MGC16275* | 2.34 | 1.23 | 1.93 | 0.95 | 2.12 | 1.09 | 1.05E-58 |
| *SELO* | 2.10 | 1.07 | 2.03 | 1.02 | 2.06 | 1.04 | 1.13E-58 |
| *CXCL12* | 15.06 | 3.91 | 26.09 | 4.71 | 19.82 | 4.31 | 1.16E-58 |
| *RNF165* | 3.95 | 1.98 | 3.48 | 1.80 | 3.71 | 1.89 | 1.30E-58 |
| *RGS1* | 4.33 | 2.11 | 3.23 | 1.69 | 3.74 | 1.90 | 1.32E-58 |
| *HIST1H4H* | 5.55 | 2.47 | 3.83 | 1.94 | 4.61 | 2.20 | 2.00E-58 |
| *TMEM144* | 0.45 | -1.14 | 0.49 | -1.04 | 0.47 | -1.09 | 2.18E-58 |
| *DSC2* | 0.39 | -1.38 | 0.42 | -1.24 | 0.40 | -1.31 | 2.19E-58 |
| *IRS1* | 3.28 | 1.71 | 3.07 | 1.62 | 3.17 | 1.67 | 2.46E-58 |
| *LDLRAP1* | 0.41 | -1.28 | 0.44 | -1.18 | 0.43 | -1.23 | 2.48E-58 |
| *PDE4B* | 2.45 | 1.29 | 2.31 | 1.20 | 2.37 | 1.25 | 2.57E-58 |
| *APOC1* | 0.03 | -5.04 | 0.03 | -4.93 | 0.03 | -4.98 | 2.96E-58 |
| *MELK* | 4.73 | 2.24 | 3.57 | 1.84 | 4.11 | 2.04 | 3.62E-58 |
| *DNAH12* | 9.57 | 3.26 | 5.38 | 2.43 | 7.18 | 2.84 | 4.09E-58 |
| *TOP2B* | 0.48 | -1.06 | 0.51 | -0.97 | 0.49 | -1.02 | 4.12E-58 |
| *ABCC5* | 0.26 | -1.97 | 0.30 | -1.76 | 0.27 | -1.86 | 4.23E-58 |
| *ASGR1* | 0.09 | -3.53 | 0.09 | -3.46 | 0.09 | -3.50 | 4.35E-58 |
| *PPARG* | 0.31 | -1.68 | 0.29 | -1.80 | 0.30 | -1.74 | 6.88E-58 |
| *FRMD3* | 7.13 | 2.83 | 6.90 | 2.79 | 7.01 | 2.81 | 8.14E-58 |
| *DBP* | 0.45 | -1.16 | 0.50 | -0.99 | 0.47 | -1.08 | 9.15E-58 |
| *RNASE1* | 0.12 | -3.00 | 0.12 | -3.06 | 0.12 | -3.03 | 9.59E-58 |
| *DBH* | 0.42 | -1.24 | 0.43 | -1.23 | 0.42 | -1.23 | 1.40E-57 |
| *SLAMF7* | 3.66 | 1.87 | 3.23 | 1.69 | 3.44 | 1.78 | 1.58E-57 |
| *ITGA11* | 0.17 | -2.57 | 0.18 | -2.46 | 0.17 | -2.52 | 1.78E-57 |
| *PYCR1* | 3.22 | 1.69 | 2.69 | 1.43 | 2.94 | 1.56 | 1.81E-57 |
| *ACADVL* | 0.43 | -1.22 | 0.47 | -1.10 | 0.45 | -1.16 | 2.07E-57 |
| *RPGRIP1* | 0.15 | -2.77 | 0.18 | -2.51 | 0.16 | -2.64 | 2.15E-57 |
| *TMCC2* | 0.47 | -1.09 | 0.51 | -0.97 | 0.49 | -1.03 | 2.95E-57 |
| *GNB3* | 0.38 | -1.40 | 0.36 | -1.48 | 0.37 | -1.44 | 3.71E-57 |
| *SPTA1* | 5.43 | 2.44 | 4.37 | 2.13 | 4.87 | 2.28 | 4.24E-57 |
| *LOC344887* | 3.84 | 1.94 | 2.99 | 1.58 | 3.39 | 1.76 | 5.27E-57 |
| *DAB2* | 0.13 | -2.92 | 0.21 | -2.26 | 0.17 | -2.59 | 6.36E-57 |
| *AKT1S1* | 6.56 | 2.71 | 4.67 | 2.22 | 5.54 | 2.47 | 6.85E-57 |
| *LMNB1* | 4.10 | 2.04 | 3.66 | 1.87 | 3.88 | 1.95 | 1.02E-56 |
| *TOMM7* | 0.46 | -1.13 | 0.50 | -1.01 | 0.48 | -1.07 | 1.24E-56 |
| *SNX10* | 4.88 | 2.29 | 4.19 | 2.07 | 4.53 | 2.18 | 1.31E-56 |
| *MESDC1* | 2.17 | 1.12 | 2.08 | 1.06 | 2.13 | 1.09 | 1.77E-56 |
| *DNAJB13* | 0.35 | -1.51 | 0.41 | -1.29 | 0.38 | -1.40 | 2.19E-56 |
| *CASP7* | 2.63 | 1.40 | 2.39 | 1.26 | 2.51 | 1.33 | 2.39E-56 |
| *RNF43* | 3.63 | 1.86 | 3.20 | 1.68 | 3.41 | 1.77 | 2.53E-56 |
| *DTX1* | 0.41 | -1.28 | 0.48 | -1.07 | 0.44 | -1.17 | 3.23E-56 |
| *IL12A* | 3.09 | 1.63 | 2.79 | 1.48 | 2.94 | 1.55 | 4.62E-56 |
| *ACTA2* | 2.29 | 1.19 | 2.11 | 1.08 | 2.20 | 1.14 | 5.83E-56 |
| *BSPRY* | 3.06 | 1.61 | 2.82 | 1.50 | 2.94 | 1.56 | 7.86E-56 |
| *PSMB9* | 2.41 | 1.27 | 2.22 | 1.15 | 2.31 | 1.21 | 9.03E-56 |
| *NADK* | 2.26 | 1.18 | 2.11 | 1.08 | 2.19 | 1.13 | 1.01E-55 |
| *RHOC* | 2.25 | 1.17 | 1.94 | 0.95 | 2.09 | 1.06 | 1.21E-55 |
| *PANX1* | 3.29 | 1.72 | 2.64 | 1.40 | 2.95 | 1.56 | 1.46E-55 |
| *RASGEF1B* | 5.06 | 2.34 | 4.03 | 2.01 | 4.51 | 2.17 | 1.53E-55 |
| *OLFM2* | 0.46 | -1.13 | 0.48 | -1.06 | 0.47 | -1.09 | 1.70E-55 |
| *ADAM19* | 6.14 | 2.62 | 4.33 | 2.11 | 5.16 | 2.37 | 1.71E-55 |
| *ACP5* | 0.38 | -1.41 | 0.35 | -1.52 | 0.36 | -1.46 | 1.96E-55 |
| *C1GALT1* | 2.35 | 1.23 | 2.14 | 1.10 | 2.24 | 1.16 | 2.15E-55 |
| *GTPBP2* | 2.48 | 1.31 | 2.31 | 1.21 | 2.39 | 1.26 | 2.51E-55 |
| *ASPHD2* | 2.63 | 1.39 | 2.28 | 1.19 | 2.45 | 1.29 | 4.10E-55 |
| *FBP1* | 0.14 | -2.86 | 0.14 | -2.81 | 0.14 | -2.83 | 4.64E-55 |
| *TJP2* | 2.36 | 1.24 | 2.19 | 1.13 | 2.27 | 1.18 | 5.64E-55 |
| *CD70* | 2.23 | 1.16 | 1.87 | 0.90 | 2.04 | 1.03 | 5.90E-55 |
| *DPEP2* | 0.26 | -1.92 | 0.30 | -1.75 | 0.28 | -1.84 | 6.34E-55 |
| *MDK* | 28.37 | 4.83 | 25.68 | 4.68 | 26.99 | 4.75 | 6.45E-55 |
| *DNAH8* | 3.74 | 1.90 | 3.29 | 1.72 | 3.51 | 1.81 | 8.93E-55 |
| *DEPTOR* | 0.20 | -2.36 | 0.27 | -1.91 | 0.23 | -2.13 | 1.45E-54 |
| *MYO10* | 3.11 | 1.64 | 3.01 | 1.59 | 3.06 | 1.61 | 1.87E-54 |
| *TRPC1* | 0.47 | -1.08 | 0.52 | -0.93 | 0.50 | -1.01 | 2.14E-54 |
| *SLC16A10* | 0.46 | -1.11 | 0.48 | -1.05 | 0.47 | -1.08 | 2.71E-54 |
| *NUPR1* | 26.35 | 4.72 | 15.17 | 3.92 | 20.00 | 4.32 | 3.25E-54 |
| *KCNA2* | 2.49 | 1.32 | 2.24 | 1.16 | 2.36 | 1.24 | 3.55E-54 |
| *DMD* | 2.18 | 1.13 | 2.10 | 1.07 | 2.14 | 1.10 | 4.43E-54 |
| *CTSF* | 0.43 | -1.23 | 0.48 | -1.06 | 0.45 | -1.14 | 4.64E-54 |
| *PRKD2* | 3.10 | 1.63 | 2.81 | 1.49 | 2.95 | 1.56 | 5.31E-54 |
| *PSEN2* | 2.03 | 1.02 | 2.13 | 1.09 | 2.08 | 1.06 | 5.73E-54 |
| *LOC728175* | 4.53 | 2.18 | 3.47 | 1.80 | 3.97 | 1.99 | 6.84E-54 |
| *KITLG* | 11.47 | 3.52 | 8.73 | 3.13 | 10.01 | 3.32 | 7.34E-54 |
| *C10orf54* | 0.32 | -1.65 | 0.30 | -1.72 | 0.31 | -1.68 | 9.20E-54 |
| *FKBP1B* | 2.95 | 1.56 | 2.24 | 1.16 | 2.57 | 1.36 | 9.28E-54 |
| *ANKRD45* | 2.80 | 1.48 | 2.57 | 1.36 | 2.68 | 1.42 | 1.08E-53 |
| *TMPRSS3* | 2.22 | 1.15 | 1.82 | 0.86 | 2.01 | 1.01 | 1.11E-53 |
| *MICALL1* | 2.18 | 1.13 | 1.85 | 0.89 | 2.01 | 1.01 | 1.15E-53 |
| *TMC8* | 0.30 | -1.75 | 0.34 | -1.57 | 0.32 | -1.66 | 1.32E-53 |
| *DBF4B* | 4.12 | 2.04 | 3.60 | 1.85 | 3.85 | 1.95 | 1.40E-53 |
| *DYNLT1* | 3.68 | 1.88 | 3.44 | 1.78 | 3.56 | 1.83 | 1.44E-53 |
| *CDK18* | 3.81 | 1.93 | 3.47 | 1.79 | 3.64 | 1.86 | 1.62E-53 |
| *VIPR1* | 0.33 | -1.62 | 0.38 | -1.41 | 0.35 | -1.51 | 1.78E-53 |
| *TRANK1* | 3.57 | 1.84 | 3.13 | 1.65 | 3.35 | 1.74 | 1.90E-53 |
| *C15orf34* | 0.41 | -1.28 | 0.49 | -1.04 | 0.45 | -1.16 | 1.92E-53 |
| *OXNAD1* | 0.49 | -1.04 | 0.51 | -0.97 | 0.50 | -1.00 | 2.28E-53 |
| *RASGRP4* | 0.49 | -1.02 | 0.49 | -1.03 | 0.49 | -1.02 | 2.33E-53 |
| *PMFBP1* | 0.34 | -1.56 | 0.31 | -1.71 | 0.32 | -1.64 | 2.95E-53 |
| *TBC1D17* | 3.88 | 1.96 | 2.99 | 1.58 | 3.41 | 1.77 | 4.89E-53 |
| *AQP3* | 0.41 | -1.29 | 0.43 | -1.21 | 0.42 | -1.25 | 6.96E-53 |
| *YPEL3* | 0.46 | -1.11 | 0.48 | -1.05 | 0.47 | -1.08 | 7.90E-53 |
| *CD36* | 0.23 | -2.11 | 0.18 | -2.46 | 0.21 | -2.29 | 8.87E-53 |
| *ARRDC2* | 0.47 | -1.10 | 0.53 | -0.91 | 0.50 | -1.01 | 1.25E-52 |
| *TMEM106A* | 3.47 | 1.79 | 3.02 | 1.59 | 3.23 | 1.69 | 1.40E-52 |
| *FCRL4* | 3.39 | 1.76 | 2.56 | 1.36 | 2.94 | 1.56 | 1.51E-52 |
| *GSN* | 0.29 | -1.79 | 0.25 | -2.00 | 0.27 | -1.89 | 1.91E-52 |
| *STX11* | 4.14 | 2.05 | 3.21 | 1.68 | 3.65 | 1.87 | 1.93E-52 |
| *TNFSF12-TNFSF13* | 0.43 | -1.23 | 0.49 | -1.02 | 0.46 | -1.12 | 2.43E-52 |
| *PIWIL4* | 6.61 | 2.72 | 5.71 | 2.51 | 6.14 | 2.62 | 2.61E-52 |
| *TANK* | 2.50 | 1.32 | 2.02 | 1.02 | 2.25 | 1.17 | 3.12E-52 |
| *C8orf73* | 0.42 | -1.24 | 0.50 | -0.99 | 0.46 | -1.12 | 3.92E-52 |
| *CDKN1C* | 5.07 | 2.34 | 5.02 | 2.33 | 5.04 | 2.33 | 4.11E-52 |
| *TUBB2A* | 5.18 | 2.37 | 4.27 | 2.09 | 4.70 | 2.23 | 4.25E-52 |
| *IL27* | 33.49 | 5.07 | 24.74 | 4.63 | 28.78 | 4.85 | 5.16E-52 |
| *CYBB* | 0.30 | -1.76 | 0.31 | -1.70 | 0.30 | -1.73 | 5.97E-52 |
| *FOLR2* | 0.08 | -3.68 | 0.11 | -3.16 | 0.09 | -3.42 | 6.86E-52 |
| *VCAN* | 0.12 | -3.08 | 0.16 | -2.68 | 0.14 | -2.88 | 1.09E-51 |
| *RNF122* | 2.30 | 1.20 | 2.18 | 1.12 | 2.24 | 1.16 | 1.42E-51 |
| *SDS* | 3.89 | 1.96 | 3.64 | 1.86 | 3.76 | 1.91 | 1.42E-51 |
| *ABTB2* | 11.36 | 3.51 | 8.41 | 3.07 | 9.77 | 3.29 | 1.50E-51 |
| *RARRES1* | 0.10 | -3.35 | 0.13 | -2.95 | 0.11 | -3.15 | 1.56E-51 |
| *HLA-G* | 2.52 | 1.33 | 2.33 | 1.22 | 2.42 | 1.28 | 1.84E-51 |
| *MTHFD1L* | 3.08 | 1.62 | 2.60 | 1.38 | 2.83 | 1.50 | 3.40E-51 |
| *ADAM12* | 0.33 | -1.62 | 0.41 | -1.29 | 0.37 | -1.45 | 3.69E-51 |
| *UACA* | 0.32 | -1.66 | 0.37 | -1.43 | 0.34 | -1.55 | 6.18E-51 |
| *WDR63* | 4.43 | 2.15 | 4.24 | 2.08 | 4.33 | 2.12 | 6.61E-51 |
| *SLFN13* | 2.91 | 1.54 | 2.42 | 1.28 | 2.66 | 1.41 | 6.71E-51 |
| *ENDOU* | 2.40 | 1.26 | 2.04 | 1.03 | 2.21 | 1.14 | 7.38E-51 |
| *ANKRD34A* | 0.49 | -1.04 | 0.50 | -1.00 | 0.49 | -1.02 | 7.43E-51 |
| *KIAA2022* | 0.31 | -1.67 | 0.40 | -1.30 | 0.36 | -1.49 | 1.14E-50 |
| *EPX* | 0.40 | -1.32 | 0.46 | -1.13 | 0.43 | -1.22 | 1.17E-50 |
| *ACPP* | 0.11 | -3.13 | 0.15 | -2.73 | 0.13 | -2.93 | 1.58E-50 |
| *PLXDC2* | 0.16 | -2.69 | 0.14 | -2.79 | 0.15 | -2.74 | 1.60E-50 |
| *IL16* | 0.47 | -1.10 | 0.49 | -1.02 | 0.48 | -1.06 | 1.97E-50 |
| *APOE* | 0.04 | -4.82 | 0.04 | -4.73 | 0.04 | -4.78 | 2.33E-50 |
| *SMPD3* | 2.83 | 1.50 | 2.88 | 1.53 | 2.86 | 1.51 | 2.39E-50 |
| *SEMA4C* | 0.21 | -2.26 | 0.30 | -1.76 | 0.25 | -2.01 | 3.62E-50 |
| *FBXO39* | 9.27 | 3.21 | 6.46 | 2.69 | 7.74 | 2.95 | 3.83E-50 |
| *GPRIN1* | 4.21 | 2.07 | 3.57 | 1.84 | 3.87 | 1.95 | 3.90E-50 |
| *LRP1* | 0.38 | -1.40 | 0.33 | -1.59 | 0.35 | -1.50 | 4.92E-50 |
| *GBP5* | 6.57 | 2.72 | 6.03 | 2.59 | 6.30 | 2.65 | 5.03E-50 |
| *C5orf62* | 2.61 | 1.38 | 2.01 | 1.01 | 2.29 | 1.20 | 5.75E-50 |
| *C1orf204* | 0.37 | -1.43 | 0.39 | -1.36 | 0.38 | -1.40 | 5.86E-50 |
| *MEFV* | 5.15 | 2.37 | 4.76 | 2.25 | 4.95 | 2.31 | 6.13E-50 |
| *DNAJC6* | 5.78 | 2.53 | 3.95 | 1.98 | 4.78 | 2.26 | 6.61E-50 |
| *LHFP* | 3.43 | 1.78 | 2.82 | 1.50 | 3.11 | 1.64 | 7.91E-50 |
| *AMPD2* | 0.46 | -1.11 | 0.50 | -1.00 | 0.48 | -1.05 | 7.99E-50 |
| *USP6NL* | 2.93 | 1.55 | 2.68 | 1.42 | 2.80 | 1.49 | 8.07E-50 |
| *MB21D1* | 3.58 | 1.84 | 3.35 | 1.75 | 3.46 | 1.79 | 8.34E-50 |
| *APOL1* | 4.04 | 2.01 | 3.71 | 1.89 | 3.87 | 1.95 | 8.81E-50 |
| *CLECL1* | 2.17 | 1.11 | 1.90 | 0.92 | 2.03 | 1.02 | 1.05E-49 |
| *OPLAH* | 0.40 | -1.31 | 0.45 | -1.16 | 0.43 | -1.23 | 1.12E-49 |
| *SEMA6C* | 0.38 | -1.39 | 0.40 | -1.31 | 0.39 | -1.35 | 1.22E-49 |
| *ATP6V0A1* | 0.36 | -1.48 | 0.36 | -1.48 | 0.36 | -1.48 | 1.25E-49 |
| *FRMD4B* | 0.31 | -1.70 | 0.38 | -1.39 | 0.34 | -1.54 | 1.31E-49 |
| *NHSL2* | 0.37 | -1.45 | 0.36 | -1.49 | 0.36 | -1.47 | 2.01E-49 |
| *SIGLECP3* | 0.13 | -2.93 | 0.22 | -2.18 | 0.17 | -2.56 | 2.08E-49 |
| *MFI2* | 2.80 | 1.49 | 2.60 | 1.38 | 2.70 | 1.43 | 2.10E-49 |
| *LOC643988* | 0.47 | -1.09 | 0.50 | -1.00 | 0.48 | -1.04 | 2.14E-49 |
| *KRT72* | 0.17 | -2.56 | 0.18 | -2.49 | 0.17 | -2.52 | 2.66E-49 |
| *BBS12* | 3.54 | 1.82 | 2.78 | 1.47 | 3.13 | 1.65 | 2.70E-49 |
| *LY96* | 0.45 | -1.14 | 0.46 | -1.13 | 0.46 | -1.14 | 3.21E-49 |
| *IGF2BP2* | 2.23 | 1.15 | 2.21 | 1.14 | 2.22 | 1.15 | 3.29E-49 |
| *NRSN2* | 2.58 | 1.37 | 2.15 | 1.10 | 2.35 | 1.24 | 3.32E-49 |
| *NOXA1* | 0.31 | -1.68 | 0.36 | -1.47 | 0.34 | -1.57 | 4.25E-49 |
| *TTLL2* | 0.39 | -1.37 | 0.44 | -1.17 | 0.41 | -1.27 | 4.30E-49 |
| *LOC389333* | 3.59 | 1.84 | 3.11 | 1.64 | 3.34 | 1.74 | 4.45E-49 |
| *CYP27A1* | 0.29 | -1.76 | 0.29 | -1.79 | 0.29 | -1.78 | 4.62E-49 |
| *GBP1P1* | 23.48 | 4.55 | 25.99 | 4.70 | 24.70 | 4.63 | 5.10E-49 |
| *C6orf97* | 0.16 | -2.63 | 0.28 | -1.83 | 0.21 | -2.23 | 6.90E-49 |
| *MASTL* | 3.94 | 1.98 | 3.30 | 1.72 | 3.61 | 1.85 | 7.16E-49 |
| *PGLYRP2* | 0.50 | -0.99 | 0.44 | -1.18 | 0.47 | -1.08 | 1.06E-48 |
| *SLC2A12* | 2.54 | 1.34 | 2.30 | 1.20 | 2.42 | 1.27 | 1.23E-48 |
| *ZNFX1* | 3.79 | 1.92 | 3.41 | 1.77 | 3.60 | 1.85 | 1.67E-48 |
| *RAB20* | 2.87 | 1.52 | 2.36 | 1.24 | 2.60 | 1.38 | 1.76E-48 |
| *MRC1* | 0.16 | -2.60 | 0.18 | -2.46 | 0.17 | -2.53 | 1.86E-48 |
| *KLF5* | 4.91 | 2.30 | 4.38 | 2.13 | 4.64 | 2.21 | 1.90E-48 |
| *L3MBTL4* | 2.11 | 1.08 | 1.97 | 0.98 | 2.04 | 1.03 | 2.08E-48 |
| *KIAA0930* | 0.43 | -1.23 | 0.44 | -1.18 | 0.43 | -1.21 | 3.60E-48 |
| *SLCO5A1* | 4.46 | 2.16 | 3.50 | 1.81 | 3.95 | 1.98 | 4.01E-48 |
| *SGCD* | 0.33 | -1.62 | 0.46 | -1.12 | 0.39 | -1.37 | 4.57E-48 |
| *VAT1* | 0.41 | -1.28 | 0.43 | -1.21 | 0.42 | -1.24 | 5.49E-48 |
| *TRPM4* | 2.72 | 1.44 | 2.75 | 1.46 | 2.73 | 1.45 | 6.33E-48 |
| *PTGER2* | 3.98 | 1.99 | 3.19 | 1.67 | 3.56 | 1.83 | 6.77E-48 |
| *ATP2B1* | 0.43 | -1.22 | 0.50 | -0.99 | 0.46 | -1.11 | 9.96E-48 |
| *LAT2* | 0.34 | -1.57 | 0.36 | -1.47 | 0.35 | -1.52 | 1.22E-47 |
| *AKAP2* | 4.23 | 2.08 | 3.86 | 1.95 | 4.04 | 2.02 | 1.25E-47 |
| *NR1H3* | 9.82 | 3.30 | 6.20 | 2.63 | 7.80 | 2.96 | 1.73E-47 |
| *ALDH7A1* | 0.31 | -1.70 | 0.34 | -1.54 | 0.33 | -1.62 | 1.79E-47 |
| *STAP1* | 3.58 | 1.84 | 3.26 | 1.71 | 3.42 | 1.77 | 2.01E-47 |
| *VSIG8* | 0.46 | -1.11 | 0.48 | -1.06 | 0.47 | -1.09 | 2.04E-47 |
| *TAP2* | 3.17 | 1.66 | 2.90 | 1.54 | 3.03 | 1.60 | 2.06E-47 |
| *FAM108C1* | 12.47 | 3.64 | 6.21 | 2.64 | 8.80 | 3.14 | 2.33E-47 |
| *NAT8L* | 0.34 | -1.56 | 0.43 | -1.22 | 0.38 | -1.39 | 2.61E-47 |
| *KCNN1* | 6.71 | 2.75 | 3.83 | 1.94 | 5.07 | 2.34 | 2.70E-47 |
| *FCER2* | 0.34 | -1.55 | 0.36 | -1.46 | 0.35 | -1.50 | 2.77E-47 |
| *SIRPD* | 0.29 | -1.77 | 0.31 | -1.68 | 0.30 | -1.73 | 2.80E-47 |
| *LILRB4* | 4.83 | 2.27 | 3.84 | 1.94 | 4.31 | 2.11 | 2.84E-47 |
| *NEURL3* | 86.05 | 6.43 | 45.80 | 5.52 | 62.78 | 5.97 | 3.04E-47 |
| *PLA2G4C* | 2.93 | 1.55 | 2.48 | 1.31 | 2.69 | 1.43 | 4.60E-47 |
| *KLHDC7B* | 8.86 | 3.15 | 7.28 | 2.86 | 8.03 | 3.01 | 4.96E-47 |
| *ZFHX3* | 0.43 | -1.21 | 0.50 | -0.99 | 0.47 | -1.10 | 5.33E-47 |
| *BST1* | 0.21 | -2.25 | 0.26 | -1.92 | 0.24 | -2.08 | 5.45E-47 |
| *IRF1* | 2.28 | 1.19 | 2.14 | 1.10 | 2.21 | 1.14 | 5.61E-47 |
| *MGAM* | 0.18 | -2.47 | 0.18 | -2.50 | 0.18 | -2.49 | 5.74E-47 |
| *GBP2* | 2.04 | 1.03 | 2.05 | 1.04 | 2.04 | 1.03 | 6.01E-47 |
| *FONG* | 0.37 | -1.45 | 0.50 | -1.00 | 0.43 | -1.22 | 8.84E-47 |
| *CDK1* | 2.33 | 1.22 | 2.19 | 1.13 | 2.26 | 1.18 | 9.33E-47 |
| *UBE2S* | 3.70 | 1.89 | 3.12 | 1.64 | 3.40 | 1.77 | 1.11E-46 |
| *NMUR1* | 0.37 | -1.44 | 0.37 | -1.45 | 0.37 | -1.44 | 1.15E-46 |
| *ANTXRL* | 0.37 | -1.43 | 0.37 | -1.45 | 0.37 | -1.44 | 1.17E-46 |
| *RNASE2* | 4.28 | 2.10 | 2.91 | 1.54 | 3.53 | 1.82 | 1.40E-46 |
| *CR1L* | 10.88 | 3.44 | 9.19 | 3.20 | 10.00 | 3.32 | 1.43E-46 |
| *KY* | 9.33 | 3.22 | 5.63 | 2.49 | 7.25 | 2.86 | 1.51E-46 |
| *FAM127C* | 0.30 | -1.75 | 0.38 | -1.39 | 0.34 | -1.57 | 1.81E-46 |
| *PLCB2* | 0.32 | -1.62 | 0.36 | -1.46 | 0.34 | -1.54 | 2.89E-46 |
| *SOX8* | 2.82 | 1.49 | 2.71 | 1.44 | 2.76 | 1.47 | 3.14E-46 |
| *IL18BP* | 0.36 | -1.47 | 0.40 | -1.33 | 0.38 | -1.40 | 3.21E-46 |
| *HLF* | 0.44 | -1.20 | 0.52 | -0.94 | 0.48 | -1.07 | 3.27E-46 |
| *CD72* | 3.59 | 1.84 | 3.33 | 1.74 | 3.46 | 1.79 | 3.33E-46 |
| *PSRC1* | 0.21 | -2.23 | 0.28 | -1.83 | 0.25 | -2.03 | 3.70E-46 |
| *NFXL1* | 0.43 | -1.22 | 0.44 | -1.19 | 0.43 | -1.20 | 4.66E-46 |
| *LOC100506746* | 2.29 | 1.20 | 1.98 | 0.99 | 2.13 | 1.09 | 5.00E-46 |
| *ACHE* | 5.29 | 2.40 | 4.85 | 2.28 | 5.07 | 2.34 | 5.38E-46 |
| *SORT1* | 0.30 | -1.76 | 0.27 | -1.89 | 0.28 | -1.83 | 6.10E-46 |
| *SERPINH1* | 2.19 | 1.13 | 2.23 | 1.15 | 2.21 | 1.14 | 6.91E-46 |
| *RIMBP3* | 0.45 | -1.14 | 0.51 | -0.97 | 0.48 | -1.05 | 7.70E-46 |
| *SLC2A9* | 0.21 | -2.24 | 0.27 | -1.88 | 0.24 | -2.06 | 7.84E-46 |
| *RIN2* | 3.96 | 1.99 | 3.56 | 1.83 | 3.76 | 1.91 | 9.90E-46 |
| *ERBB3* | 0.38 | -1.40 | 0.36 | -1.47 | 0.37 | -1.43 | 1.08E-45 |
| *MOCOS* | 3.72 | 1.89 | 2.81 | 1.49 | 3.23 | 1.69 | 1.13E-45 |
| *TSPAN4* | 0.24 | -2.06 | 0.31 | -1.68 | 0.27 | -1.87 | 1.97E-45 |
| *BPGM* | 2.32 | 1.21 | 2.00 | 1.00 | 2.15 | 1.11 | 2.20E-45 |
| *NUDT6* | 0.46 | -1.12 | 0.53 | -0.90 | 0.50 | -1.01 | 2.29E-45 |
| *COL18A1* | 0.41 | -1.28 | 0.48 | -1.06 | 0.44 | -1.17 | 2.51E-45 |
| *FAM63A* | 0.43 | -1.20 | 0.51 | -0.98 | 0.47 | -1.09 | 2.86E-45 |
| *FAM40B* | 2.59 | 1.37 | 2.65 | 1.41 | 2.62 | 1.39 | 3.08E-45 |
| *RBCK1* | 2.57 | 1.36 | 2.32 | 1.21 | 2.44 | 1.29 | 3.29E-45 |
| *IGF2BP3* | 4.94 | 2.31 | 4.05 | 2.02 | 4.47 | 2.16 | 3.66E-45 |
| *CFB* | 13.52 | 3.76 | 16.15 | 4.01 | 14.78 | 3.89 | 5.41E-45 |
| *GBP6* | 2.42 | 1.27 | 2.09 | 1.07 | 2.25 | 1.17 | 5.43E-45 |
| *LOC100505702* | 0.10 | -3.30 | 0.12 | -3.02 | 0.11 | -3.16 | 5.50E-45 |
| *FAM72B* | 5.82 | 2.54 | 5.41 | 2.44 | 5.61 | 2.49 | 9.53E-45 |
| *GRIN3A* | 4.72 | 2.24 | 4.91 | 2.30 | 4.82 | 2.27 | 1.21E-44 |
| *SPON1* | 0.40 | -1.34 | 0.46 | -1.10 | 0.43 | -1.22 | 1.27E-44 |
| *HLA-DOB* | 2.31 | 1.21 | 2.19 | 1.13 | 2.25 | 1.17 | 1.75E-44 |
| *MUC1* | 6.76 | 2.76 | 5.29 | 2.40 | 5.98 | 2.58 | 1.93E-44 |
| *CALD1* | 16.73 | 4.06 | 9.57 | 3.26 | 12.65 | 3.66 | 2.27E-44 |
| *KIAA1522* | 5.04 | 2.33 | 4.24 | 2.09 | 4.63 | 2.21 | 2.37E-44 |
| *TNK2* | 2.83 | 1.50 | 2.55 | 1.35 | 2.68 | 1.42 | 2.50E-44 |
| *CBWD2* | 3.12 | 1.64 | 3.00 | 1.59 | 3.06 | 1.61 | 2.66E-44 |
| *ILDR1* | 5.53 | 2.47 | 3.71 | 1.89 | 4.53 | 2.18 | 2.78E-44 |
| *SERTAD1* | 2.19 | 1.13 | 1.94 | 0.96 | 2.06 | 1.05 | 3.18E-44 |
| *AANAT* | 2.37 | 1.25 | 1.95 | 0.96 | 2.15 | 1.11 | 3.21E-44 |
| *PAX5* | 2.48 | 1.31 | 2.44 | 1.28 | 2.46 | 1.30 | 3.80E-44 |
| *KLF7* | 0.48 | -1.06 | 0.51 | -0.97 | 0.50 | -1.01 | 4.06E-44 |
| *CLEC5A* | 0.05 | -4.28 | 0.10 | -3.29 | 0.07 | -3.79 | 4.80E-44 |
| *ADPRHL2* | 2.20 | 1.13 | 2.14 | 1.10 | 2.17 | 1.12 | 6.06E-44 |
| *TGFBI* | 0.36 | -1.47 | 0.36 | -1.47 | 0.36 | -1.47 | 6.09E-44 |
| *ADORA2B* | 0.26 | -1.94 | 0.26 | -1.92 | 0.26 | -1.93 | 8.23E-44 |
| *LRRC25* | 0.28 | -1.81 | 0.34 | -1.56 | 0.31 | -1.69 | 8.39E-44 |
| *CLEC7A* | 0.36 | -1.48 | 0.35 | -1.51 | 0.36 | -1.49 | 1.09E-43 |
| *KRT73* | 0.23 | -2.14 | 0.21 | -2.25 | 0.22 | -2.20 | 1.09E-43 |
| *ALPK2* | 0.46 | -1.12 | 0.50 | -1.01 | 0.48 | -1.07 | 1.13E-43 |
| *ANKRD33B* | 2.90 | 1.54 | 2.38 | 1.25 | 2.63 | 1.39 | 1.35E-43 |
| *DKFZp761E198* | 2.46 | 1.30 | 2.09 | 1.06 | 2.26 | 1.18 | 1.59E-43 |
| *RNF19B* | 5.39 | 2.43 | 3.89 | 1.96 | 4.58 | 2.19 | 1.63E-43 |
| *RET* | 14.60 | 3.87 | 10.56 | 3.40 | 12.41 | 3.63 | 1.70E-43 |
| *ALDH3A2* | 0.41 | -1.27 | 0.46 | -1.14 | 0.43 | -1.20 | 1.72E-43 |
| *SCARB2* | 2.47 | 1.30 | 2.16 | 1.11 | 2.31 | 1.21 | 1.86E-43 |
| *CLMP* | 6.03 | 2.59 | 5.12 | 2.36 | 5.56 | 2.47 | 3.15E-43 |
| *FRRS1* | 2.14 | 1.10 | 2.17 | 1.12 | 2.15 | 1.11 | 3.62E-43 |
| *ZMYND10* | 0.47 | -1.09 | 0.46 | -1.12 | 0.46 | -1.11 | 6.54E-43 |
| *FAM72D* | 4.80 | 2.26 | 4.38 | 2.13 | 4.59 | 2.20 | 6.72E-43 |
| *DPEP3* | 0.44 | -1.19 | 0.38 | -1.41 | 0.41 | -1.30 | 7.11E-43 |
| *SSTR3* | 7.31 | 2.87 | 7.15 | 2.84 | 7.23 | 2.85 | 8.47E-43 |
| *CD40* | 4.70 | 2.23 | 3.93 | 1.97 | 4.30 | 2.10 | 9.67E-43 |
| *ATP9A* | 0.44 | -1.18 | 0.50 | -1.00 | 0.47 | -1.09 | 9.84E-43 |
| *RELB* | 2.32 | 1.21 | 1.98 | 0.99 | 2.14 | 1.10 | 1.15E-42 |
| *LOC100505622* | 0.46 | -1.12 | 0.50 | -0.99 | 0.48 | -1.05 | 1.35E-42 |
| *DEPDC7* | 0.25 | -1.97 | 0.32 | -1.66 | 0.28 | -1.82 | 1.36E-42 |
| *SIAH3* | 0.36 | -1.47 | 0.40 | -1.31 | 0.38 | -1.39 | 1.39E-42 |
| *MIR548AN* | 0.34 | -1.55 | 0.42 | -1.26 | 0.38 | -1.41 | 1.51E-42 |
| *AMICA1* | 0.20 | -2.35 | 0.24 | -2.07 | 0.22 | -2.21 | 1.74E-42 |
| *H3F3C* | 2.01 | 1.01 | 2.02 | 1.01 | 2.01 | 1.01 | 2.18E-42 |
| *HDX* | 4.40 | 2.14 | 4.37 | 2.13 | 4.38 | 2.13 | 2.25E-42 |
| *ADM2* | 2.13 | 1.09 | 1.97 | 0.98 | 2.05 | 1.04 | 2.32E-42 |
| *NCF1B* | 5.07 | 2.34 | 4.03 | 2.01 | 4.52 | 2.18 | 2.32E-42 |
| *PTPRU* | 16.63 | 4.06 | 13.16 | 3.72 | 14.79 | 3.89 | 2.81E-42 |
| *SPAG8* | 0.42 | -1.25 | 0.45 | -1.16 | 0.43 | -1.21 | 2.84E-42 |
| *NFIX* | 2.54 | 1.35 | 2.34 | 1.23 | 2.44 | 1.29 | 3.98E-42 |
| *ODF2L* | 2.17 | 1.12 | 1.98 | 0.99 | 2.07 | 1.05 | 4.15E-42 |
| *S1PR1* | 0.45 | -1.17 | 0.49 | -1.02 | 0.47 | -1.09 | 4.28E-42 |
| *MGC39372* | 4.47 | 2.16 | 3.65 | 1.87 | 4.04 | 2.01 | 7.20E-42 |
| *OTOF* | 44.28 | 5.47 | 27.99 | 4.81 | 35.21 | 5.14 | 8.35E-42 |
| *LRP12* | 3.62 | 1.85 | 2.92 | 1.54 | 3.25 | 1.70 | 8.49E-42 |
| *FGF9* | 0.34 | -1.55 | 0.40 | -1.31 | 0.37 | -1.43 | 8.97E-42 |
| *GPR180* | 2.56 | 1.35 | 2.38 | 1.25 | 2.47 | 1.30 | 9.63E-42 |
| *GBP7* | 12.67 | 3.66 | 8.64 | 3.11 | 10.46 | 3.39 | 1.20E-41 |
| *WNK2* | 4.59 | 2.20 | 3.47 | 1.79 | 3.99 | 2.00 | 1.23E-41 |
| *TRIM56* | 2.18 | 1.12 | 2.00 | 1.00 | 2.09 | 1.06 | 1.25E-41 |
| *DRAM1* | 4.43 | 2.15 | 3.66 | 1.87 | 4.03 | 2.01 | 1.32E-41 |
| *ACAN* | 2.65 | 1.40 | 2.34 | 1.23 | 2.49 | 1.32 | 1.50E-41 |
| *LOC100287216* | 0.49 | -1.04 | 0.51 | -0.97 | 0.50 | -1.00 | 1.53E-41 |
| *VENTX* | 0.44 | -1.17 | 0.50 | -1.00 | 0.47 | -1.08 | 1.77E-41 |
| *RBM43* | 2.30 | 1.20 | 2.32 | 1.22 | 2.31 | 1.21 | 2.02E-41 |
| *SLFN12* | 2.52 | 1.34 | 2.34 | 1.23 | 2.43 | 1.28 | 2.73E-41 |
| *JAG1* | 2.91 | 1.54 | 2.69 | 1.43 | 2.80 | 1.49 | 3.09E-41 |
| *GIPC3* | 0.47 | -1.09 | 0.51 | -0.96 | 0.49 | -1.02 | 3.18E-41 |
| *APOL6* | 3.35 | 1.75 | 3.22 | 1.69 | 3.29 | 1.72 | 3.45E-41 |
| *ANKRD18A* | 0.38 | -1.39 | 0.36 | -1.46 | 0.37 | -1.42 | 5.10E-41 |
| *NRG1* | 0.10 | -3.33 | 0.11 | -3.20 | 0.10 | -3.26 | 5.20E-41 |
| *APOBEC3G* | 2.22 | 1.15 | 1.94 | 0.96 | 2.07 | 1.05 | 5.95E-41 |
| *PML* | 5.38 | 2.43 | 5.06 | 2.34 | 5.22 | 2.38 | 6.27E-41 |
| *ACSM1* | 2.57 | 1.36 | 2.54 | 1.35 | 2.56 | 1.35 | 6.86E-41 |
| *DSG2* | 0.38 | -1.38 | 0.49 | -1.02 | 0.44 | -1.20 | 9.88E-41 |
| *KANK2* | 0.21 | -2.26 | 0.32 | -1.65 | 0.26 | -1.96 | 9.89E-41 |
| *SIGLEC10* | 0.22 | -2.19 | 0.20 | -2.35 | 0.21 | -2.27 | 1.16E-40 |
| *ADAMTS10* | 0.36 | -1.47 | 0.39 | -1.35 | 0.38 | -1.41 | 1.50E-40 |
| *CSRNP1* | 4.35 | 2.12 | 3.45 | 1.79 | 3.87 | 1.95 | 1.53E-40 |
| *RGS20* | 3.14 | 1.65 | 2.37 | 1.25 | 2.73 | 1.45 | 1.60E-40 |
| *MYO1E* | 2.89 | 1.53 | 2.37 | 1.24 | 2.61 | 1.39 | 1.86E-40 |
| *LGALS17A* | 15.16 | 3.92 | 18.32 | 4.20 | 16.66 | 4.06 | 2.52E-40 |
| *C1R* | 4.28 | 2.10 | 3.90 | 1.96 | 4.09 | 2.03 | 3.26E-40 |
| *DOK2* | 0.29 | -1.79 | 0.30 | -1.73 | 0.30 | -1.76 | 3.79E-40 |
| *CLEC10A* | 0.05 | -4.27 | 0.03 | -4.85 | 0.04 | -4.56 | 3.85E-40 |
| *SMAD6* | 0.16 | -2.65 | 0.16 | -2.61 | 0.16 | -2.63 | 4.33E-40 |
| *CASS4* | 0.37 | -1.42 | 0.42 | -1.24 | 0.40 | -1.33 | 4.93E-40 |
| *CRIP3* | 0.35 | -1.53 | 0.41 | -1.30 | 0.37 | -1.42 | 9.75E-40 |
| *PRICKLE1* | 2.02 | 1.02 | 2.01 | 1.00 | 2.01 | 1.01 | 1.03E-39 |
| *CACNA1D* | 0.38 | -1.40 | 0.41 | -1.27 | 0.40 | -1.34 | 1.46E-39 |
| *BTG3* | 3.26 | 1.71 | 2.46 | 1.30 | 2.83 | 1.50 | 1.51E-39 |
| *C10orf128* | 0.31 | -1.70 | 0.44 | -1.19 | 0.37 | -1.44 | 1.51E-39 |
| *CDC45* | 2.41 | 1.27 | 1.97 | 0.98 | 2.18 | 1.12 | 1.59E-39 |
| *C21orf67* | 0.34 | -1.55 | 0.32 | -1.67 | 0.33 | -1.61 | 2.04E-39 |
| *MOV10* | 4.09 | 2.03 | 3.79 | 1.92 | 3.94 | 1.98 | 2.11E-39 |
| *ARHGAP23* | 12.03 | 3.59 | 9.81 | 3.29 | 10.86 | 3.44 | 2.16E-39 |
| *CTSL1* | 5.91 | 2.56 | 3.64 | 1.86 | 4.64 | 2.21 | 3.61E-39 |
| *CDC42EP1* | 4.90 | 2.29 | 5.72 | 2.52 | 5.30 | 2.41 | 3.80E-39 |
| *SYTL3* | 2.20 | 1.14 | 1.95 | 0.97 | 2.07 | 1.05 | 5.04E-39 |
| *FABP3* | 0.17 | -2.52 | 0.20 | -2.29 | 0.19 | -2.41 | 5.17E-39 |
| *SLC31A2* | 3.17 | 1.66 | 2.62 | 1.39 | 2.88 | 1.53 | 6.65E-39 |
| *RAB3IL1* | 0.15 | -2.72 | 0.28 | -1.86 | 0.20 | -2.29 | 7.29E-39 |
| *GUCY2C* | 0.34 | -1.55 | 0.30 | -1.74 | 0.32 | -1.65 | 9.58E-39 |
| *TDO2* | 31.98 | 5.00 | 13.80 | 3.79 | 21.01 | 4.39 | 9.64E-39 |
| *CD80* | 18.71 | 4.23 | 14.54 | 3.86 | 16.49 | 4.04 | 1.02E-38 |
| *CD200* | 2.12 | 1.09 | 1.99 | 0.99 | 2.06 | 1.04 | 1.31E-38 |
| *TSPAN32* | 0.27 | -1.88 | 0.28 | -1.83 | 0.28 | -1.86 | 1.31E-38 |
| *SNX18* | 0.40 | -1.34 | 0.51 | -0.96 | 0.45 | -1.15 | 1.40E-38 |
| *SPSB1* | 4.78 | 2.26 | 3.16 | 1.66 | 3.89 | 1.96 | 1.48E-38 |
| *SMCHD1* | 2.42 | 1.27 | 2.33 | 1.22 | 2.38 | 1.25 | 1.58E-38 |
| *SMTNL1* | 6.01 | 2.59 | 4.53 | 2.18 | 5.22 | 2.38 | 1.64E-38 |
| *ZNF704* | 0.19 | -2.42 | 0.29 | -1.77 | 0.23 | -2.09 | 1.69E-38 |
| *SLC29A3* | 0.38 | -1.41 | 0.39 | -1.36 | 0.38 | -1.38 | 1.75E-38 |
| *PDK4* | 0.15 | -2.72 | 0.16 | -2.63 | 0.16 | -2.68 | 2.19E-38 |
| *TMTC1* | 6.76 | 2.76 | 5.52 | 2.46 | 6.10 | 2.61 | 2.82E-38 |
| *FAH* | 0.29 | -1.77 | 0.38 | -1.41 | 0.33 | -1.59 | 3.10E-38 |
| *TRIM47* | 0.31 | -1.71 | 0.37 | -1.44 | 0.34 | -1.58 | 3.36E-38 |
| *ST18* | 0.15 | -2.78 | 0.21 | -2.27 | 0.17 | -2.52 | 3.48E-38 |
| *RASGRF1* | 0.42 | -1.24 | 0.38 | -1.40 | 0.40 | -1.32 | 3.74E-38 |
| *CACNA2D4* | 0.35 | -1.51 | 0.42 | -1.26 | 0.38 | -1.38 | 4.51E-38 |
| *EPS8* | 0.22 | -2.21 | 0.33 | -1.62 | 0.27 | -1.91 | 4.71E-38 |
| *CD274* | 12.42 | 3.63 | 8.28 | 3.05 | 10.14 | 3.34 | 6.48E-38 |
| *KISS1R* | 0.46 | -1.11 | 0.46 | -1.10 | 0.46 | -1.11 | 6.55E-38 |
| *TSC22D1* | 2.42 | 1.27 | 1.97 | 0.98 | 2.18 | 1.13 | 8.69E-38 |
| *RASGRP2* | 0.41 | -1.28 | 0.42 | -1.27 | 0.41 | -1.27 | 1.12E-37 |
| *MIR4712* | 0.47 | -1.10 | 0.48 | -1.05 | 0.47 | -1.07 | 1.12E-37 |
| *SIRPG* | 0.48 | -1.05 | 0.49 | -1.02 | 0.49 | -1.03 | 1.16E-37 |
| *PRSS23* | 0.45 | -1.16 | 0.50 | -1.00 | 0.47 | -1.08 | 1.20E-37 |
| *C6orf145* | 2.35 | 1.23 | 2.01 | 1.00 | 2.17 | 1.12 | 1.26E-37 |
| *LOC100506068* | 2.59 | 1.37 | 2.49 | 1.31 | 2.54 | 1.34 | 1.49E-37 |
| *LOC642826* | 2.52 | 1.33 | 2.40 | 1.26 | 2.46 | 1.30 | 1.72E-37 |
| *VAMP5* | 3.37 | 1.75 | 3.30 | 1.72 | 3.34 | 1.74 | 1.85E-37 |
| *NCF1C* | 5.61 | 2.49 | 4.44 | 2.15 | 4.99 | 2.32 | 2.03E-37 |
| *SLC16A6* | 0.39 | -1.36 | 0.35 | -1.51 | 0.37 | -1.43 | 2.08E-37 |
| *WDR52* | 0.47 | -1.09 | 0.52 | -0.94 | 0.49 | -1.02 | 2.11E-37 |
| *SCLT1* | 2.65 | 1.40 | 2.45 | 1.29 | 2.55 | 1.35 | 2.18E-37 |
| *N4BP1* | 2.94 | 1.56 | 2.40 | 1.26 | 2.66 | 1.41 | 2.47E-37 |
| *WARS* | 3.30 | 1.72 | 2.88 | 1.53 | 3.08 | 1.62 | 2.71E-37 |
| *SLC40A1* | 0.46 | -1.13 | 0.54 | -0.89 | 0.50 | -1.01 | 2.80E-37 |
| *GPER* | 2.83 | 1.50 | 2.07 | 1.05 | 2.42 | 1.27 | 3.26E-37 |
| *CIR1* | 2.90 | 1.53 | 2.30 | 1.20 | 2.58 | 1.37 | 3.37E-37 |
| *MGAT3* | 2.10 | 1.07 | 2.17 | 1.11 | 2.13 | 1.09 | 3.44E-37 |
| *LOC100507547* | 0.45 | -1.16 | 0.51 | -0.98 | 0.48 | -1.07 | 4.42E-37 |
| *TCN2* | 5.53 | 2.47 | 5.07 | 2.34 | 5.30 | 2.40 | 5.20E-37 |
| *NEFM* | 0.40 | -1.34 | 0.53 | -0.92 | 0.46 | -1.13 | 5.90E-37 |
| *MICALCL* | 0.44 | -1.19 | 0.48 | -1.06 | 0.46 | -1.12 | 6.06E-37 |
| *CCL3L3* | 2.69 | 1.43 | 2.32 | 1.21 | 2.45 | 1.29 | 7.91E-37 |
| *SERPING1* | 8.44 | 3.08 | 8.68 | 3.12 | 8.56 | 3.10 | 7.93E-37 |
| *ALDH1A1* | 0.16 | -2.67 | 0.15 | -2.74 | 0.15 | -2.70 | 8.42E-37 |
| *PAR5* | 0.42 | -1.24 | 0.42 | -1.26 | 0.42 | -1.25 | 8.46E-37 |
| *NCF1* | 4.44 | 2.15 | 3.74 | 1.90 | 4.08 | 2.03 | 9.30E-37 |
| *CACNA1A* | 15.62 | 3.96 | 12.63 | 3.66 | 14.04 | 3.81 | 9.30E-37 |
| *MPEG1* | 0.43 | -1.22 | 0.43 | -1.21 | 0.43 | -1.22 | 1.12E-36 |
| *CXCL11* | 56.73 | 5.83 | 61.67 | 5.95 | 59.15 | 5.89 | 1.17E-36 |
| *BRCA2* | 3.45 | 1.79 | 3.13 | 1.65 | 3.29 | 1.72 | 1.99E-36 |
| *LRRC6* | 0.36 | -1.47 | 0.46 | -1.13 | 0.41 | -1.30 | 2.01E-36 |
| *GAS2L3* | 0.34 | -1.56 | 0.36 | -1.46 | 0.35 | -1.51 | 2.02E-36 |
| *SGMS2* | 0.08 | -3.62 | 0.14 | -2.82 | 0.11 | -3.22 | 2.09E-36 |
| *IFITM2* | 3.69 | 1.88 | 3.81 | 1.93 | 3.75 | 1.91 | 2.09E-36 |
| *LOC100505666* | 0.39 | -1.37 | 0.48 | -1.07 | 0.43 | -1.22 | 2.31E-36 |
| *SEMA4A* | 4.55 | 2.19 | 3.68 | 1.88 | 4.09 | 2.03 | 2.38E-36 |
| *LOC100131347* | 0.37 | -1.45 | 0.38 | -1.40 | 0.37 | -1.43 | 3.04E-36 |
| *SH2B2* | 3.23 | 1.69 | 2.83 | 1.50 | 3.02 | 1.60 | 3.41E-36 |
| *KCTD19* | 6.34 | 2.66 | 7.55 | 2.92 | 6.92 | 2.79 | 3.76E-36 |
| *KIF5A* | 0.45 | -1.16 | 0.56 | -0.85 | 0.50 | -1.00 | 4.00E-36 |
| *C13orf15* | 0.45 | -1.17 | 0.47 | -1.09 | 0.46 | -1.13 | 4.41E-36 |
| *COL19A1* | 0.39 | -1.35 | 0.46 | -1.14 | 0.42 | -1.24 | 4.77E-36 |
| *DUSP5* | 8.51 | 3.09 | 5.95 | 2.57 | 7.12 | 2.83 | 5.06E-36 |
| *CNP* | 3.26 | 1.71 | 3.09 | 1.63 | 3.17 | 1.67 | 5.64E-36 |
| *TMEM229B* | 3.94 | 1.98 | 3.49 | 1.80 | 3.71 | 1.89 | 6.19E-36 |
| *SOCS2* | 3.71 | 1.89 | 2.71 | 1.44 | 3.17 | 1.66 | 7.75E-36 |
| *ATP10A* | 2.46 | 1.30 | 2.56 | 1.36 | 2.51 | 1.33 | 9.25E-36 |
| *GPR155* | 3.25 | 1.70 | 3.05 | 1.61 | 3.15 | 1.66 | 1.06E-35 |
| *IPW* | 0.49 | -1.02 | 0.50 | -1.01 | 0.50 | -1.01 | 1.10E-35 |
| *SMAGP* | 2.18 | 1.12 | 2.13 | 1.09 | 2.15 | 1.11 | 1.19E-35 |
| *MOBKL2C* | 2.35 | 1.23 | 2.10 | 1.07 | 2.22 | 1.15 | 1.23E-35 |
| *LOC113230* | 0.30 | -1.75 | 0.37 | -1.45 | 0.33 | -1.60 | 1.38E-35 |
| *CENPK* | 0.48 | -1.07 | 0.50 | -1.01 | 0.49 | -1.04 | 1.79E-35 |
| *ISG20* | 7.68 | 2.94 | 7.05 | 2.82 | 7.36 | 2.88 | 1.79E-35 |
| *SLFN12L* | 2.46 | 1.30 | 2.08 | 1.06 | 2.27 | 1.18 | 2.36E-35 |
| *TREX1* | 3.91 | 1.97 | 3.68 | 1.88 | 3.79 | 1.92 | 2.63E-35 |
| *SIGIRR* | 0.43 | -1.22 | 0.47 | -1.07 | 0.45 | -1.15 | 2.84E-35 |
| *ZNF702P* | 2.96 | 1.57 | 2.58 | 1.36 | 2.76 | 1.47 | 3.06E-35 |
| *FBXO6* | 4.73 | 2.24 | 4.16 | 2.06 | 4.43 | 2.15 | 3.24E-35 |
| *CHST7* | 3.42 | 1.78 | 2.08 | 1.05 | 2.67 | 1.41 | 3.37E-35 |
| *SLC1A4* | 5.40 | 2.43 | 3.39 | 1.76 | 4.28 | 2.10 | 3.52E-35 |
| *ST14* | 0.29 | -1.76 | 0.31 | -1.71 | 0.30 | -1.74 | 3.71E-35 |
| *NKAPL* | 2.66 | 1.41 | 2.15 | 1.10 | 2.39 | 1.26 | 3.83E-35 |
| *S100A8* | 0.25 | -2.02 | 0.24 | -2.06 | 0.24 | -2.04 | 4.22E-35 |
| *FLJ13197* | 0.42 | -1.26 | 0.43 | -1.20 | 0.43 | -1.23 | 4.79E-35 |
| *CHL1* | 0.47 | -1.10 | 0.47 | -1.10 | 0.47 | -1.10 | 4.92E-35 |
| *CDKN2B* | 4.00 | 2.00 | 3.11 | 1.64 | 3.52 | 1.82 | 5.11E-35 |
| *SULT1B1* | 0.41 | -1.29 | 0.49 | -1.04 | 0.45 | -1.16 | 6.02E-35 |
| *LOC283663* | 0.41 | -1.27 | 0.45 | -1.15 | 0.43 | -1.21 | 6.07E-35 |
| *CD163L1* | 0.25 | -1.99 | 0.33 | -1.58 | 0.29 | -1.79 | 6.33E-35 |
| *LOC286442* | 0.33 | -1.59 | 0.39 | -1.37 | 0.36 | -1.48 | 6.51E-35 |
| *LOC100131096* | 0.48 | -1.05 | 0.50 | -1.01 | 0.49 | -1.03 | 7.16E-35 |
| *NLRP12* | 0.44 | -1.19 | 0.32 | -1.64 | 0.37 | -1.42 | 7.72E-35 |
| *BAI1* | 3.94 | 1.98 | 3.79 | 1.92 | 3.86 | 1.95 | 1.02E-34 |
| *CDKN2A* | 2.18 | 1.13 | 1.96 | 0.97 | 2.07 | 1.05 | 1.09E-34 |
| *LOC100128682* | 2.72 | 1.44 | 2.28 | 1.19 | 2.49 | 1.32 | 1.37E-34 |
| *NACAD* | 2.57 | 1.36 | 2.08 | 1.06 | 2.31 | 1.21 | 1.48E-34 |
| *BACE2* | 2.15 | 1.10 | 1.89 | 0.92 | 2.02 | 1.01 | 1.50E-34 |
| *CREM* | 3.54 | 1.83 | 2.46 | 1.30 | 2.95 | 1.56 | 1.69E-34 |
| *STAT2* | 2.59 | 1.37 | 2.69 | 1.43 | 2.64 | 1.40 | 2.51E-34 |
| *HES4* | 46.33 | 5.53 | 31.94 | 5.00 | 38.46 | 5.27 | 2.62E-34 |
| *CLDN23* | 5.28 | 2.40 | 3.33 | 1.74 | 4.20 | 2.07 | 2.68E-34 |
| *BARD1* | 2.51 | 1.33 | 2.44 | 1.29 | 2.48 | 1.31 | 2.91E-34 |
| *FAM13A* | 0.47 | -1.08 | 0.52 | -0.93 | 0.50 | -1.01 | 3.44E-34 |
| *SLC48A1* | 0.37 | -1.43 | 0.50 | -1.00 | 0.43 | -1.22 | 3.50E-34 |
| *C1orf173* | 31.55 | 4.98 | 36.06 | 5.17 | 33.73 | 5.08 | 3.80E-34 |
| *PLIN2* | 0.36 | -1.46 | 0.39 | -1.37 | 0.37 | -1.42 | 4.19E-34 |
| *TRIM2* | 0.42 | -1.25 | 0.47 | -1.09 | 0.44 | -1.17 | 4.52E-34 |
| *HNMT* | 0.19 | -2.37 | 0.23 | -2.10 | 0.21 | -2.23 | 4.75E-34 |
| *AXL* | 9.19 | 3.20 | 9.38 | 3.23 | 9.28 | 3.21 | 4.82E-34 |
| *TICAM2* | 2.33 | 1.22 | 2.19 | 1.13 | 2.26 | 1.18 | 4.95E-34 |
| *SH3TC1* | 0.48 | -1.06 | 0.47 | -1.09 | 0.48 | -1.07 | 5.52E-34 |
| *TMEM217* | 3.09 | 1.63 | 2.43 | 1.28 | 2.74 | 1.45 | 5.89E-34 |
| *CBWD1* | 2.20 | 1.13 | 2.08 | 1.06 | 2.14 | 1.10 | 6.65E-34 |
| *SLC24A4* | 0.39 | -1.36 | 0.38 | -1.39 | 0.39 | -1.37 | 6.93E-34 |
| *LOC100130987* | 0.48 | -1.06 | 0.45 | -1.14 | 0.47 | -1.10 | 7.26E-34 |
| *SOAT1* | 2.29 | 1.20 | 2.17 | 1.12 | 2.23 | 1.16 | 1.07E-33 |
| *NMI* | 2.99 | 1.58 | 2.80 | 1.49 | 2.90 | 1.53 | 1.07E-33 |
| *CDHR2* | 0.45 | -1.17 | 0.52 | -0.95 | 0.48 | -1.06 | 1.14E-33 |
| *ITGA7* | 1.99 | 1.00 | 2.07 | 1.05 | 2.03 | 1.02 | 1.28E-33 |
| *XCR1* | 5.78 | 2.53 | 4.05 | 2.02 | 4.84 | 2.27 | 1.55E-33 |
| *CPNE9* | 0.41 | -1.28 | 0.47 | -1.10 | 0.44 | -1.19 | 1.70E-33 |
| *COBL* | 15.93 | 3.99 | 12.95 | 3.69 | 14.36 | 3.84 | 1.75E-33 |
| *ITIH1* | 2.41 | 1.27 | 1.96 | 0.97 | 2.17 | 1.12 | 1.94E-33 |
| *AVPR2* | 0.45 | -1.14 | 0.43 | -1.21 | 0.44 | -1.18 | 2.24E-33 |
| *NDUFC2-KCTD14* | 17.09 | 4.10 | 15.09 | 3.92 | 16.06 | 4.01 | 2.49E-33 |
| *C19orf66* | 3.13 | 1.65 | 2.85 | 1.51 | 2.99 | 1.58 | 2.50E-33 |
| *PHGDH* | 2.83 | 1.50 | 2.31 | 1.21 | 2.56 | 1.36 | 2.52E-33 |
| *ANKRD29* | 10.70 | 3.42 | 5.77 | 2.53 | 7.85 | 2.97 | 2.65E-33 |
| *ARHGEF10L* | 2.59 | 1.37 | 2.42 | 1.28 | 2.50 | 1.32 | 2.73E-33 |
| *TUBB2B* | 5.14 | 2.36 | 3.75 | 1.91 | 4.39 | 2.13 | 2.78E-33 |
| *LIME1* | 0.38 | -1.40 | 0.37 | -1.42 | 0.38 | -1.41 | 3.27E-33 |
| *IFITM1* | 4.30 | 2.10 | 4.09 | 2.03 | 4.19 | 2.07 | 3.35E-33 |
| *LOC84989* | 2.41 | 1.27 | 1.89 | 0.91 | 2.13 | 1.09 | 3.77E-33 |
| *ICAM5* | 0.41 | -1.30 | 0.39 | -1.35 | 0.40 | -1.32 | 3.90E-33 |
| *TRIM25* | 2.72 | 1.44 | 2.54 | 1.34 | 2.63 | 1.39 | 4.11E-33 |
| *ZBP1* | 7.65 | 2.94 | 6.34 | 2.66 | 6.96 | 2.80 | 4.31E-33 |
| *MAP2* | 3.59 | 1.84 | 3.78 | 1.92 | 3.68 | 1.88 | 5.21E-33 |
| *GPD2* | 2.34 | 1.23 | 2.11 | 1.08 | 2.23 | 1.15 | 5.54E-33 |
| *CD2AP* | 2.03 | 1.02 | 2.01 | 1.01 | 2.02 | 1.02 | 5.56E-33 |
| *TMEM171* | 2.84 | 1.51 | 3.46 | 1.79 | 3.14 | 1.65 | 6.12E-33 |
| *HIST1H4K* | 2.29 | 1.20 | 2.00 | 1.00 | 2.14 | 1.10 | 6.22E-33 |
| *SLC6A8* | 2.85 | 1.51 | 2.32 | 1.21 | 2.57 | 1.36 | 6.62E-33 |
| *TRIM36* | 2.51 | 1.33 | 2.25 | 1.17 | 2.38 | 1.25 | 7.04E-33 |
| *SCNN1A* | 0.41 | -1.29 | 0.45 | -1.17 | 0.43 | -1.23 | 7.19E-33 |
| *CYP1B1* | 0.39 | -1.38 | 0.47 | -1.10 | 0.42 | -1.24 | 7.76E-33 |
| *GBP1* | 6.74 | 2.75 | 7.30 | 2.87 | 7.02 | 2.81 | 8.18E-33 |
| *PTGDR* | 0.50 | -0.99 | 0.43 | -1.23 | 0.46 | -1.11 | 8.86E-33 |
| *CLDN7* | 0.43 | -1.21 | 0.48 | -1.04 | 0.46 | -1.13 | 1.04E-32 |
| *KRT1* | 0.19 | -2.40 | 0.24 | -2.06 | 0.21 | -2.23 | 1.27E-32 |
| *O3FAR1* | 0.30 | -1.73 | 0.32 | -1.64 | 0.31 | -1.68 | 1.44E-32 |
| *SYNPO2* | 4.18 | 2.06 | 3.23 | 1.69 | 3.67 | 1.88 | 1.64E-32 |
| *ARNT2* | 22.51 | 4.49 | 8.08 | 3.02 | 13.49 | 3.75 | 1.64E-32 |
| *GATM* | 0.28 | -1.82 | 0.45 | -1.16 | 0.36 | -1.49 | 1.90E-32 |
| *SATB2* | 0.32 | -1.64 | 0.43 | -1.23 | 0.37 | -1.43 | 2.16E-32 |
| *BRSK1* | 2.10 | 1.07 | 2.02 | 1.01 | 2.06 | 1.04 | 2.21E-32 |
| *NCRNA00161* | 4.25 | 2.09 | 3.26 | 1.70 | 3.72 | 1.90 | 2.22E-32 |
| *HDAC9* | 2.46 | 1.30 | 2.09 | 1.06 | 2.27 | 1.18 | 2.25E-32 |
| *PARP11* | 2.65 | 1.41 | 2.50 | 1.32 | 2.58 | 1.37 | 2.82E-32 |
| *RHBDF2* | 2.35 | 1.23 | 1.99 | 0.99 | 2.16 | 1.11 | 3.06E-32 |
| *SQRDL* | 2.54 | 1.34 | 2.05 | 1.04 | 2.28 | 1.19 | 3.30E-32 |
| *CD40LG* | 0.44 | -1.19 | 0.54 | -0.89 | 0.49 | -1.04 | 3.59E-32 |
| *FOSL1* | 6.18 | 2.63 | 4.29 | 2.10 | 5.15 | 2.36 | 3.87E-32 |
| *NAIP* | 0.41 | -1.28 | 0.42 | -1.27 | 0.41 | -1.27 | 4.00E-32 |
| *PHF16* | 2.10 | 1.07 | 2.00 | 1.00 | 2.05 | 1.04 | 4.05E-32 |
| *GPR44* | 0.44 | -1.18 | 0.52 | -0.95 | 0.48 | -1.06 | 4.08E-32 |
| *ATP13A2* | 3.22 | 1.69 | 3.05 | 1.61 | 3.13 | 1.65 | 4.14E-32 |
| *TBC1D10C* | 0.47 | -1.08 | 0.49 | -1.04 | 0.48 | -1.06 | 4.24E-32 |
| *PADI2* | 0.06 | -4.13 | 0.12 | -3.09 | 0.08 | -3.61 | 4.29E-32 |
| *LGR4* | 0.32 | -1.63 | 0.42 | -1.25 | 0.37 | -1.44 | 5.50E-32 |
| *EDNRB* | 0.08 | -3.70 | 0.15 | -2.75 | 0.11 | -3.23 | 5.95E-32 |
| *FLJ44635* | 0.38 | -1.38 | 0.39 | -1.35 | 0.39 | -1.36 | 6.83E-32 |
| *NFAM1* | 0.34 | -1.55 | 0.39 | -1.36 | 0.36 | -1.46 | 7.24E-32 |
| *PPM1K* | 3.13 | 1.65 | 2.81 | 1.49 | 2.97 | 1.57 | 7.59E-32 |
| *FAM72A* | 6.20 | 2.63 | 5.52 | 2.47 | 5.85 | 2.55 | 7.67E-32 |
| *LAMB1* | 2.42 | 1.28 | 1.96 | 0.97 | 2.18 | 1.12 | 8.45E-32 |
| *NCRNA00346* | 2.38 | 1.25 | 2.21 | 1.14 | 2.29 | 1.20 | 1.01E-31 |
| *NTN4* | 0.47 | -1.10 | 0.50 | -0.99 | 0.48 | -1.05 | 1.03E-31 |
| *CYP1B1-AS1* | 0.41 | -1.29 | 0.40 | -1.32 | 0.40 | -1.31 | 1.06E-31 |
| *SAMD4A* | 7.77 | 2.96 | 7.30 | 2.87 | 7.53 | 2.91 | 1.07E-31 |
| *ELOVL3* | 17.96 | 4.17 | 19.92 | 4.32 | 18.91 | 4.24 | 1.38E-31 |
| *LRRC70* | 0.43 | -1.21 | 0.52 | -0.93 | 0.47 | -1.07 | 1.66E-31 |
| *NEK6* | 0.45 | -1.15 | 0.42 | -1.24 | 0.44 | -1.19 | 1.75E-31 |
| *CTDSPL* | 0.47 | -1.08 | 0.47 | -1.10 | 0.47 | -1.09 | 1.84E-31 |
| *CCL24* | 0.02 | -5.70 | 0.07 | -3.93 | 0.04 | -4.81 | 1.89E-31 |
| *TLR3* | 5.82 | 2.54 | 5.56 | 2.47 | 5.69 | 2.51 | 2.34E-31 |
| *SLC7A5* | 2.77 | 1.47 | 2.21 | 1.14 | 2.47 | 1.31 | 2.41E-31 |
| *HDC* | 2.61 | 1.38 | 1.95 | 0.96 | 2.26 | 1.17 | 2.66E-31 |
| *RAPGEF5* | 2.21 | 1.14 | 2.23 | 1.16 | 2.22 | 1.15 | 3.19E-31 |
| *DSC1* | 0.28 | -1.83 | 0.31 | -1.68 | 0.30 | -1.75 | 3.35E-31 |
| *TRIM5* | 3.35 | 1.74 | 3.15 | 1.65 | 3.25 | 1.70 | 4.19E-31 |
| *ELL3* | 2.16 | 1.11 | 1.98 | 0.98 | 2.07 | 1.05 | 4.66E-31 |
| *SLAMF8* | 0.30 | -1.73 | 0.29 | -1.79 | 0.30 | -1.76 | 6.35E-31 |
| *ASCL2* | 3.20 | 1.68 | 3.52 | 1.82 | 3.36 | 1.75 | 7.21E-31 |
| *LYSMD2* | 2.64 | 1.40 | 2.26 | 1.18 | 2.44 | 1.29 | 7.23E-31 |
| *ST3GAL5* | 2.74 | 1.45 | 2.45 | 1.29 | 2.59 | 1.37 | 8.23E-31 |
| *PRRG4* | 1.89 | 0.92 | 2.14 | 1.10 | 2.01 | 1.01 | 9.22E-31 |
| *NT5C3* | 8.83 | 3.14 | 8.57 | 3.10 | 8.70 | 3.12 | 9.89E-31 |
| *LHFPL2* | 2.35 | 1.23 | 2.03 | 1.02 | 2.19 | 1.13 | 1.29E-30 |
| *ZNF618* | 3.52 | 1.81 | 3.35 | 1.75 | 3.44 | 1.78 | 1.47E-30 |
| *S100A12* | 0.25 | -2.00 | 0.17 | -2.57 | 0.21 | -2.28 | 1.49E-30 |
| *C2* | 3.04 | 1.61 | 3.25 | 1.70 | 3.15 | 1.65 | 2.15E-30 |
| *PLAC9* | 0.50 | -0.99 | 0.44 | -1.17 | 0.47 | -1.08 | 2.33E-30 |
| *NAPA* | 2.49 | 1.31 | 2.37 | 1.24 | 2.43 | 1.28 | 2.35E-30 |
| *NRGN* | 0.29 | -1.77 | 0.27 | -1.87 | 0.28 | -1.82 | 2.36E-30 |
| *LOC441617* | 0.36 | -1.46 | 0.36 | -1.48 | 0.36 | -1.47 | 2.72E-30 |
| *IFI27* | 17.05 | 4.09 | 14.59 | 3.87 | 15.77 | 3.98 | 3.08E-30 |
| *PILRA* | 2.49 | 1.32 | 1.94 | 0.95 | 2.20 | 1.14 | 3.33E-30 |
| *PI4K2B* | 3.71 | 1.89 | 3.37 | 1.75 | 3.54 | 1.82 | 3.42E-30 |
| *CPNE5* | 2.81 | 1.49 | 2.79 | 1.48 | 2.80 | 1.49 | 3.83E-30 |
| *TNFSF13B* | 3.34 | 1.74 | 3.34 | 1.74 | 3.34 | 1.74 | 3.87E-30 |
| *SERPINB9* | 2.42 | 1.27 | 1.98 | 0.98 | 2.19 | 1.13 | 4.13E-30 |
| *FAM59A* | 10.41 | 3.38 | 8.06 | 3.01 | 9.16 | 3.20 | 4.13E-30 |
| *FAR2* | 2.28 | 1.19 | 2.04 | 1.03 | 2.16 | 1.11 | 4.16E-30 |
| *FAM46A* | 2.23 | 1.16 | 2.26 | 1.17 | 2.24 | 1.17 | 4.41E-30 |
| *HSH2D* | 4.56 | 2.19 | 4.10 | 2.04 | 4.32 | 2.11 | 4.60E-30 |
| *C11orf21* | 0.41 | -1.28 | 0.39 | -1.35 | 0.40 | -1.31 | 6.54E-30 |
| *PHLDA2* | 3.74 | 1.90 | 3.29 | 1.72 | 3.51 | 1.81 | 7.92E-30 |
| *TUFT1* | 2.83 | 1.50 | 2.60 | 1.38 | 2.71 | 1.44 | 8.92E-30 |
| *RAB39* | 2.48 | 1.31 | 2.29 | 1.20 | 2.38 | 1.25 | 9.25E-30 |
| *ANKRD53* | 3.62 | 1.86 | 2.97 | 1.57 | 3.28 | 1.71 | 1.18E-29 |
| *HIST2H2BF* | 3.00 | 1.59 | 2.46 | 1.30 | 2.72 | 1.44 | 1.26E-29 |
| *TPSAB1* | 2.68 | 1.42 | 2.07 | 1.05 | 2.36 | 1.24 | 1.37E-29 |
| *SRC* | 4.23 | 2.08 | 2.91 | 1.54 | 3.51 | 1.81 | 1.39E-29 |
| *CD27* | 0.45 | -1.16 | 0.49 | -1.03 | 0.47 | -1.10 | 1.45E-29 |
| *SLC47A1* | 0.10 | -3.31 | 0.14 | -2.81 | 0.12 | -3.06 | 1.46E-29 |
| *BMF* | 0.27 | -1.87 | 0.40 | -1.32 | 0.33 | -1.59 | 1.62E-29 |
| *TRIM21* | 3.18 | 1.67 | 2.89 | 1.53 | 3.03 | 1.60 | 1.63E-29 |
| *SHISA8* | 0.43 | -1.20 | 0.47 | -1.08 | 0.45 | -1.14 | 1.67E-29 |
| *FAM26F* | 10.82 | 3.44 | 16.40 | 4.04 | 13.32 | 3.74 | 1.77E-29 |
| *ZNF185* | 0.48 | -1.06 | 0.50 | -1.00 | 0.49 | -1.03 | 2.40E-29 |
| *FOXC1* | 10.60 | 3.41 | 9.81 | 3.29 | 10.20 | 3.35 | 3.10E-29 |
| *CPM* | 0.27 | -1.90 | 0.37 | -1.42 | 0.32 | -1.66 | 3.10E-29 |
| *FTSJD2* | 2.58 | 1.37 | 2.49 | 1.32 | 2.53 | 1.34 | 3.25E-29 |
| *VAV3* | 2.32 | 1.21 | 1.88 | 0.91 | 2.09 | 1.06 | 3.48E-29 |
| *CD109* | 3.36 | 1.75 | 2.35 | 1.23 | 2.81 | 1.49 | 3.89E-29 |
| *RNF213* | 2.30 | 1.20 | 2.21 | 1.15 | 2.26 | 1.17 | 4.52E-29 |
| *CIB2* | 4.50 | 2.17 | 4.17 | 2.06 | 4.33 | 2.11 | 4.66E-29 |
| *TIAM2* | 3.10 | 1.63 | 3.31 | 1.73 | 3.20 | 1.68 | 4.98E-29 |
| *DCUN1D3* | 2.50 | 1.32 | 1.85 | 0.89 | 2.15 | 1.10 | 5.18E-29 |
| *FGD2* | 2.57 | 1.36 | 2.81 | 1.49 | 2.69 | 1.43 | 5.63E-29 |
| *CXCR2P1* | 12.54 | 3.65 | 14.98 | 3.91 | 13.71 | 3.78 | 6.92E-29 |
| *GJA3* | 15.85 | 3.99 | 8.16 | 3.03 | 11.37 | 3.51 | 7.08E-29 |
| *TMEM150B* | 2.09 | 1.06 | 1.93 | 0.95 | 2.01 | 1.01 | 8.93E-29 |
| *GLP1R* | 3.26 | 1.71 | 2.65 | 1.41 | 2.94 | 1.56 | 1.02E-28 |
| *NFE2L3* | 3.23 | 1.69 | 3.13 | 1.65 | 3.18 | 1.67 | 1.06E-28 |
| *TDRD7* | 3.82 | 1.93 | 3.34 | 1.74 | 3.57 | 1.84 | 1.20E-28 |
| *AGPAT9* | 0.35 | -1.53 | 0.45 | -1.14 | 0.40 | -1.34 | 1.21E-28 |
| *CACHD1* | 0.45 | -1.17 | 0.56 | -0.84 | 0.50 | -1.00 | 1.44E-28 |
| *IL1RN* | 23.33 | 4.54 | 9.57 | 3.26 | 14.94 | 3.90 | 1.66E-28 |
| *PGAP1* | 6.60 | 2.72 | 5.84 | 2.55 | 6.21 | 2.63 | 1.86E-28 |
| *PLSCR2* | 3.37 | 1.75 | 2.74 | 1.46 | 3.04 | 1.60 | 2.40E-28 |
| *ZCCHC2* | 3.04 | 1.60 | 2.68 | 1.42 | 2.85 | 1.51 | 2.53E-28 |
| *ITPKA* | 2.49 | 1.31 | 1.95 | 0.97 | 2.20 | 1.14 | 2.93E-28 |
| *SNCA* | 0.46 | -1.11 | 0.53 | -0.93 | 0.49 | -1.02 | 3.24E-28 |
| *HK2* | 3.47 | 1.80 | 2.52 | 1.33 | 2.96 | 1.56 | 3.86E-28 |
| *IL7* | 3.14 | 1.65 | 2.61 | 1.39 | 2.86 | 1.52 | 4.08E-28 |
| *FNDC3B* | 3.11 | 1.64 | 2.14 | 1.09 | 2.58 | 1.37 | 4.21E-28 |
| *LOC648809* | 2.53 | 1.34 | 2.18 | 1.12 | 2.35 | 1.23 | 4.52E-28 |
| *GUCY1A3* | 3.99 | 2.00 | 4.10 | 2.04 | 4.04 | 2.02 | 5.65E-28 |
| *EPHB3* | 0.36 | -1.49 | 0.35 | -1.52 | 0.35 | -1.51 | 6.14E-28 |
| *UBE2QL1* | 4.75 | 2.25 | 2.96 | 1.56 | 3.75 | 1.91 | 6.47E-28 |
| *FGFBP2* | 0.38 | -1.41 | 0.43 | -1.23 | 0.40 | -1.32 | 6.89E-28 |
| *TNF* | 3.56 | 1.83 | 2.58 | 1.37 | 3.03 | 1.60 | 6.94E-28 |
| *FUT4* | 3.50 | 1.81 | 2.98 | 1.57 | 3.23 | 1.69 | 7.12E-28 |
| *RILP* | 2.37 | 1.25 | 2.09 | 1.06 | 2.23 | 1.15 | 7.77E-28 |
| *RUFY4* | 9.69 | 3.28 | 15.76 | 3.98 | 12.36 | 3.63 | 8.88E-28 |
| *UNC5B* | 0.33 | -1.58 | 0.35 | -1.50 | 0.34 | -1.54 | 8.91E-28 |
| *FOLR3* | 0.33 | -1.61 | 0.39 | -1.34 | 0.36 | -1.48 | 9.25E-28 |
| *PTTG2* | 3.31 | 1.73 | 2.56 | 1.35 | 2.91 | 1.54 | 9.51E-28 |
| *CCL4* | 5.01 | 2.33 | 3.91 | 1.97 | 4.43 | 2.15 | 1.04E-27 |
| *C22orf28* | 2.61 | 1.38 | 2.54 | 1.34 | 2.57 | 1.36 | 1.15E-27 |
| *MIR4645* | 3.58 | 1.84 | 2.76 | 1.47 | 3.15 | 1.65 | 1.24E-27 |
| *SP100* | 2.37 | 1.25 | 2.19 | 1.13 | 2.28 | 1.19 | 1.59E-27 |
| *GDPD3* | 0.46 | -1.11 | 0.48 | -1.07 | 0.47 | -1.09 | 1.71E-27 |
| *F13A1* | 0.03 | -4.84 | 0.05 | -4.24 | 0.04 | -4.54 | 1.73E-27 |
| *TDRD6* | 0.50 | -1.00 | 0.47 | -1.08 | 0.49 | -1.04 | 1.96E-27 |
| *TNFSF10* | 10.30 | 3.36 | 12.21 | 3.61 | 11.21 | 3.49 | 2.11E-27 |
| *COLEC12* | 0.13 | -2.89 | 0.24 | -2.09 | 0.18 | -2.49 | 2.54E-27 |
| *VRK2* | 2.30 | 1.20 | 1.90 | 0.93 | 2.09 | 1.07 | 2.76E-27 |
| *OASL* | 9.70 | 3.28 | 7.94 | 2.99 | 8.78 | 3.13 | 2.87E-27 |
| *PHF11* | 2.50 | 1.32 | 2.35 | 1.23 | 2.43 | 1.28 | 3.04E-27 |
| *NTN1* | 4.10 | 2.03 | 3.44 | 1.78 | 3.76 | 1.91 | 3.13E-27 |
| *MEP1A* | 4.98 | 2.32 | 3.12 | 1.64 | 3.94 | 1.98 | 3.47E-27 |
| *PAX8* | 2.77 | 1.47 | 2.67 | 1.42 | 2.72 | 1.44 | 3.49E-27 |
| *ALOX5* | 0.43 | -1.21 | 0.36 | -1.46 | 0.40 | -1.34 | 3.55E-27 |
| *ACSBG1* | 2.32 | 1.22 | 2.23 | 1.16 | 2.28 | 1.19 | 4.92E-27 |
| *MZB1* | 2.48 | 1.31 | 2.68 | 1.42 | 2.58 | 1.37 | 5.73E-27 |
| *LDHAL6B* | 5.34 | 2.42 | 2.83 | 1.50 | 3.88 | 1.96 | 5.78E-27 |
| *SORBS1* | 2.15 | 1.10 | 1.89 | 0.92 | 2.01 | 1.01 | 6.00E-27 |
| *LY86* | 0.41 | -1.30 | 0.44 | -1.20 | 0.42 | -1.25 | 6.04E-27 |
| *OSBPL5* | 2.24 | 1.16 | 1.97 | 0.98 | 2.10 | 1.07 | 6.21E-27 |
| *NLRP7* | 2.81 | 1.49 | 2.40 | 1.26 | 2.59 | 1.37 | 6.28E-27 |
| *CHRNA6* | 9.63 | 3.27 | 4.60 | 2.20 | 6.65 | 2.73 | 8.29E-27 |
| *CETP* | 3.01 | 1.59 | 2.60 | 1.38 | 2.80 | 1.48 | 1.18E-26 |
| *GAS1* | 0.41 | -1.29 | 0.49 | -1.03 | 0.45 | -1.16 | 1.25E-26 |
| *RASGRP3* | 2.58 | 1.37 | 2.30 | 1.20 | 2.44 | 1.28 | 1.31E-26 |
| *GPC4* | 0.19 | -2.40 | 0.15 | -2.70 | 0.17 | -2.55 | 1.37E-26 |
| *LAMP3* | 4.86 | 2.28 | 3.80 | 1.93 | 4.30 | 2.10 | 1.45E-26 |
| *BHLHE41* | 0.26 | -1.94 | 0.37 | -1.43 | 0.31 | -1.69 | 1.55E-26 |
| *TTC21A* | 3.48 | 1.80 | 3.32 | 1.73 | 3.39 | 1.76 | 2.27E-26 |
| *CCR5* | 3.67 | 1.88 | 3.13 | 1.64 | 3.39 | 1.76 | 2.28E-26 |
| *IDO1* | 16.35 | 4.03 | 9.00 | 3.17 | 12.13 | 3.60 | 2.44E-26 |
| *ZC3HAV1L* | 2.84 | 1.51 | 2.33 | 1.22 | 2.57 | 1.36 | 2.50E-26 |
| *LOC285972* | 4.49 | 2.17 | 2.82 | 1.49 | 3.56 | 1.83 | 2.63E-26 |
| *BNC2* | 0.20 | -2.35 | 0.34 | -1.56 | 0.26 | -1.96 | 2.88E-26 |
| *KCNMB4* | 0.43 | -1.23 | 0.55 | -0.86 | 0.48 | -1.05 | 2.94E-26 |
| *FXYD6* | 3.47 | 1.80 | 3.72 | 1.90 | 3.59 | 1.85 | 3.45E-26 |
| *KCNMB1* | 4.43 | 2.15 | 3.54 | 1.82 | 3.96 | 1.98 | 3.49E-26 |
| *SDC2* | 0.44 | -1.20 | 0.37 | -1.42 | 0.40 | -1.31 | 4.00E-26 |
| *ACCN2* | 0.42 | -1.24 | 0.49 | -1.03 | 0.46 | -1.13 | 4.29E-26 |
| *RAPH1* | 0.41 | -1.30 | 0.49 | -1.02 | 0.45 | -1.16 | 4.36E-26 |
| *FLJ46906* | 0.41 | -1.29 | 0.42 | -1.24 | 0.42 | -1.27 | 4.84E-26 |
| *LGR6* | 0.45 | -1.14 | 0.50 | -1.00 | 0.48 | -1.07 | 5.60E-26 |
| *CCL4L1* | 3.97 | 1.99 | 3.20 | 1.68 | 3.56 | 1.83 | 5.62E-26 |
| *CD209* | 0.31 | -1.69 | 0.32 | -1.65 | 0.31 | -1.67 | 5.65E-26 |
| *DLGAP1* | 2.85 | 1.51 | 2.17 | 1.12 | 2.49 | 1.32 | 7.56E-26 |
| *PLS3* | 2.26 | 1.18 | 2.11 | 1.08 | 2.18 | 1.13 | 8.22E-26 |
| *TRIM78P* | 2.78 | 1.48 | 2.60 | 1.38 | 2.69 | 1.43 | 8.43E-26 |
| *SAMD9* | 4.78 | 2.26 | 4.37 | 2.13 | 4.57 | 2.19 | 8.71E-26 |
| *UBE2C* | 2.49 | 1.32 | 2.16 | 1.11 | 2.32 | 1.21 | 9.39E-26 |
| *ESPNL* | 3.41 | 1.77 | 2.61 | 1.38 | 2.98 | 1.58 | 9.82E-26 |
| *CDA* | 0.04 | -4.73 | 0.09 | -3.44 | 0.06 | -4.08 | 1.00E-25 |
| *DYRK3* | 2.19 | 1.13 | 2.43 | 1.28 | 2.31 | 1.21 | 1.07E-25 |
| *TXLNB* | 0.33 | -1.62 | 0.30 | -1.73 | 0.31 | -1.67 | 1.16E-25 |
| *ACP2* | 2.60 | 1.38 | 2.17 | 1.12 | 2.38 | 1.25 | 1.26E-25 |
| *IRF7* | 5.17 | 2.37 | 4.50 | 2.17 | 4.82 | 2.27 | 1.56E-25 |
| *FAM47E* | 2.30 | 1.20 | 2.07 | 1.05 | 2.19 | 1.13 | 1.92E-25 |
| *TRIM6* | 2.96 | 1.56 | 2.71 | 1.44 | 2.83 | 1.50 | 1.96E-25 |
| *SPATS2L* | 5.41 | 2.44 | 4.76 | 2.25 | 5.08 | 2.34 | 2.15E-25 |
| *BATF3* | 7.50 | 2.91 | 7.59 | 2.92 | 7.54 | 2.92 | 2.25E-25 |
| *GPR82* | 0.25 | -1.97 | 0.32 | -1.66 | 0.28 | -1.82 | 2.65E-25 |
| *CECR2* | 4.64 | 2.21 | 3.71 | 1.89 | 4.15 | 2.05 | 2.67E-25 |
| *LOC283194* | 0.41 | -1.27 | 0.43 | -1.23 | 0.42 | -1.25 | 2.67E-25 |
| *FCGR2B* | 0.18 | -2.46 | 0.17 | -2.58 | 0.17 | -2.52 | 2.76E-25 |
| *TP53INP2* | 2.25 | 1.17 | 1.81 | 0.86 | 2.02 | 1.02 | 3.45E-25 |
| *CCR1* | 2.53 | 1.34 | 2.40 | 1.26 | 2.46 | 1.30 | 3.88E-25 |
| *SLC2A6* | 2.91 | 1.54 | 2.44 | 1.29 | 2.66 | 1.41 | 3.95E-25 |
| *PLEKHG7* | 4.17 | 2.06 | 3.76 | 1.91 | 3.96 | 1.99 | 4.28E-25 |
| *BCL2L14* | 28.28 | 4.82 | 35.40 | 5.15 | 31.64 | 4.98 | 4.80E-25 |
| *C1orf61* | 4.71 | 2.24 | 2.81 | 1.49 | 3.64 | 1.86 | 5.05E-25 |
| *PVRL2* | 4.87 | 2.29 | 3.38 | 1.76 | 4.06 | 2.02 | 5.32E-25 |
| *ADPRH* | 2.56 | 1.36 | 2.39 | 1.26 | 2.47 | 1.31 | 5.41E-25 |
| *NRCAM* | 0.14 | -2.86 | 0.24 | -2.07 | 0.18 | -2.46 | 5.92E-25 |
| *LGALS3BP* | 4.56 | 2.19 | 4.47 | 2.16 | 4.52 | 2.18 | 6.67E-25 |
| *STK32B* | 0.31 | -1.68 | 0.39 | -1.35 | 0.35 | -1.51 | 7.58E-25 |
| *LRRC3* | 2.41 | 1.27 | 2.34 | 1.23 | 2.37 | 1.25 | 8.60E-25 |
| *PARP10* | 3.07 | 1.62 | 2.85 | 1.51 | 2.95 | 1.56 | 8.79E-25 |
| *EPHX4* | 0.45 | -1.15 | 0.44 | -1.20 | 0.44 | -1.17 | 9.32E-25 |
| *WNT1* | 0.45 | -1.14 | 0.50 | -1.01 | 0.47 | -1.08 | 1.13E-24 |
| *PLEKHA7* | 2.59 | 1.37 | 2.49 | 1.32 | 2.54 | 1.35 | 1.16E-24 |
| *KBTBD10* | 0.44 | -1.17 | 0.56 | -0.84 | 0.50 | -1.00 | 1.22E-24 |
| *CREB5* | 0.27 | -1.89 | 0.46 | -1.12 | 0.35 | -1.50 | 1.37E-24 |
| *NLRP1* | 0.47 | -1.08 | 0.49 | -1.02 | 0.48 | -1.05 | 1.50E-24 |
| *CELSR1* | 0.49 | -1.04 | 0.41 | -1.28 | 0.45 | -1.16 | 1.54E-24 |
| *POU5F1P3* | 0.43 | -1.22 | 0.47 | -1.09 | 0.45 | -1.15 | 1.56E-24 |
| *TIMP3* | 0.12 | -3.05 | 0.19 | -2.36 | 0.15 | -2.71 | 1.73E-24 |
| *MGC45922* | 2.22 | 1.15 | 1.85 | 0.89 | 2.03 | 1.02 | 2.13E-24 |
| *IQCK* | 0.38 | -1.41 | 0.38 | -1.39 | 0.38 | -1.40 | 2.62E-24 |
| *CDR2L* | 0.42 | -1.26 | 0.56 | -0.85 | 0.48 | -1.05 | 2.84E-24 |
| *FAM53A* | 2.07 | 1.05 | 2.35 | 1.23 | 2.20 | 1.14 | 2.93E-24 |
| *NT5DC2* | 0.39 | -1.35 | 0.27 | -1.87 | 0.33 | -1.61 | 3.24E-24 |
| *SLC6A10P* | 2.62 | 1.39 | 2.53 | 1.34 | 2.58 | 1.36 | 3.38E-24 |
| *MCM10* | 2.51 | 1.33 | 2.16 | 1.11 | 2.32 | 1.22 | 4.21E-24 |
| *PRRG1* | 2.78 | 1.48 | 2.29 | 1.19 | 2.52 | 1.34 | 4.63E-24 |
| *NAPSB* | 3.05 | 1.61 | 3.06 | 1.62 | 3.06 | 1.61 | 4.79E-24 |
| *TNFRSF10C* | 0.40 | -1.31 | 0.50 | -1.01 | 0.45 | -1.16 | 6.57E-24 |
| *LOC646329* | 2.69 | 1.43 | 2.71 | 1.44 | 2.70 | 1.43 | 6.90E-24 |
| *NCRNA00304* | 0.42 | -1.27 | 0.50 | -1.01 | 0.45 | -1.14 | 7.04E-24 |
| *SUSD1* | 2.46 | 1.30 | 2.06 | 1.04 | 2.25 | 1.17 | 7.06E-24 |
| *GPX3* | 0.25 | -1.99 | 0.32 | -1.63 | 0.29 | -1.81 | 7.50E-24 |
| *CCL19* | 13.77 | 3.78 | 7.92 | 2.98 | 10.44 | 3.38 | 7.71E-24 |
| *IFIH1* | 5.73 | 2.52 | 4.87 | 2.29 | 5.28 | 2.40 | 7.97E-24 |
| *GRHL1* | 2.08 | 1.05 | 2.11 | 1.08 | 2.09 | 1.07 | 9.53E-24 |
| *GPR161* | 6.05 | 2.60 | 4.15 | 2.05 | 5.01 | 2.32 | 1.30E-23 |
| *LOC100507387* | 0.42 | -1.27 | 0.40 | -1.31 | 0.41 | -1.29 | 1.43E-23 |
| *HIST1H2BD* | 3.33 | 1.74 | 2.35 | 1.23 | 2.80 | 1.48 | 1.58E-23 |
| *DRAP1* | 2.22 | 1.15 | 2.04 | 1.03 | 2.13 | 1.09 | 1.62E-23 |
| *TMEM26* | 7.71 | 2.95 | 4.78 | 2.26 | 6.07 | 2.60 | 1.67E-23 |
| *SLC1A5* | 0.44 | -1.19 | 0.49 | -1.03 | 0.46 | -1.11 | 1.89E-23 |
| *RGS13* | 2.70 | 1.43 | 2.88 | 1.53 | 2.79 | 1.48 | 1.90E-23 |
| *LGALS9B* | 2.70 | 1.43 | 2.64 | 1.40 | 2.67 | 1.42 | 1.91E-23 |
| *DHX58* | 4.65 | 2.22 | 4.51 | 2.17 | 4.58 | 2.20 | 1.97E-23 |
| *ATF5* | 6.29 | 2.65 | 4.19 | 2.07 | 5.14 | 2.36 | 1.98E-23 |
| *TLR2* | 2.89 | 1.53 | 2.09 | 1.06 | 2.46 | 1.30 | 1.98E-23 |
| *PKMYT1* | 2.26 | 1.18 | 2.06 | 1.04 | 2.16 | 1.11 | 2.00E-23 |
| *LYPD5* | 2.40 | 1.26 | 2.14 | 1.10 | 2.27 | 1.18 | 2.04E-23 |
| *PRDM16* | 7.41 | 2.89 | 3.80 | 1.92 | 5.30 | 2.41 | 2.35E-23 |
| *OAS1* | 6.21 | 2.63 | 5.56 | 2.48 | 5.88 | 2.55 | 2.39E-23 |
| *SLC41A2* | 3.83 | 1.94 | 2.60 | 1.38 | 3.15 | 1.66 | 2.43E-23 |
| *IL4I1* | 7.97 | 3.00 | 5.23 | 2.39 | 6.46 | 2.69 | 2.71E-23 |
| *GSTM2* | 0.44 | -1.17 | 0.49 | -1.04 | 0.46 | -1.11 | 2.77E-23 |
| *TJP1* | 4.05 | 2.02 | 2.86 | 1.52 | 3.40 | 1.77 | 2.82E-23 |
| *BST2* | 2.18 | 1.12 | 2.28 | 1.19 | 2.23 | 1.16 | 3.15E-23 |
| *TTC7B* | 3.71 | 1.89 | 2.60 | 1.38 | 3.11 | 1.64 | 3.19E-23 |
| *ADAMTS7* | 0.44 | -1.19 | 0.53 | -0.93 | 0.48 | -1.06 | 3.23E-23 |
| *UBE2L6* | 3.11 | 1.64 | 2.94 | 1.56 | 3.03 | 1.60 | 3.34E-23 |
| *SLC30A4* | 2.34 | 1.22 | 1.89 | 0.92 | 2.10 | 1.07 | 3.49E-23 |
| *HIST1H2AC* | 2.23 | 1.15 | 1.88 | 0.91 | 2.04 | 1.03 | 3.63E-23 |
| *FAS-AS1* | 2.28 | 1.19 | 1.85 | 0.88 | 2.05 | 1.04 | 3.78E-23 |
| *SPHK1* | 4.31 | 2.11 | 2.71 | 1.44 | 3.41 | 1.77 | 3.97E-23 |
| *FAM153B* | 0.43 | -1.23 | 0.44 | -1.20 | 0.43 | -1.22 | 4.37E-23 |
| *SCT* | 4.35 | 2.12 | 5.34 | 2.42 | 4.82 | 2.27 | 4.60E-23 |
| *ZNF365* | 6.18 | 2.63 | 3.71 | 1.89 | 4.79 | 2.26 | 4.81E-23 |
| *RTP4* | 6.48 | 2.70 | 5.26 | 2.39 | 5.84 | 2.55 | 6.48E-23 |
| *EHD4* | 2.28 | 1.19 | 2.10 | 1.07 | 2.19 | 1.13 | 6.52E-23 |
| *EPB41L1* | 0.43 | -1.23 | 0.44 | -1.19 | 0.43 | -1.21 | 7.30E-23 |
| *KCNJ15* | 0.08 | -3.68 | 0.19 | -2.40 | 0.12 | -3.04 | 7.94E-23 |
| *ANKRD22* | 13.19 | 3.72 | 12.78 | 3.68 | 12.99 | 3.70 | 8.13E-23 |
| *PACSIN3* | 2.16 | 1.11 | 2.17 | 1.12 | 2.17 | 1.12 | 8.24E-23 |
| *LOC654433* | 2.26 | 1.18 | 2.43 | 1.28 | 2.34 | 1.23 | 8.61E-23 |
| *SSPN* | 0.42 | -1.24 | 0.38 | -1.40 | 0.40 | -1.32 | 8.84E-23 |
| *APOBEC3B* | 44.39 | 5.47 | 15.67 | 3.97 | 26.37 | 4.72 | 9.13E-23 |
| *NEURL* | 2.21 | 1.14 | 1.97 | 0.98 | 2.09 | 1.06 | 9.84E-23 |
| *NEXN* | 10.88 | 3.44 | 10.74 | 3.42 | 10.81 | 3.43 | 9.96E-23 |
| *ICAM1* | 2.88 | 1.53 | 2.27 | 1.18 | 2.56 | 1.36 | 1.01E-22 |
| *PPFIA4* | 0.33 | -1.58 | 0.42 | -1.27 | 0.37 | -1.42 | 1.14E-22 |
| *STAT1* | 2.55 | 1.35 | 2.38 | 1.25 | 2.46 | 1.30 | 1.18E-22 |
| *HIST1H2BJ* | 3.14 | 1.65 | 2.27 | 1.18 | 2.67 | 1.42 | 1.45E-22 |
| *ETV7* | 11.99 | 3.58 | 10.75 | 3.43 | 11.35 | 3.50 | 1.69E-22 |
| *UBQLNL* | 3.11 | 1.64 | 2.72 | 1.44 | 2.90 | 1.54 | 1.72E-22 |
| *C1S* | 5.08 | 2.34 | 4.13 | 2.05 | 4.58 | 2.20 | 2.01E-22 |
| *SAMSN1* | 2.46 | 1.30 | 1.94 | 0.96 | 2.19 | 1.13 | 2.04E-22 |
| *IRAK2* | 4.94 | 2.30 | 2.83 | 1.50 | 3.74 | 1.90 | 2.07E-22 |
| *C5orf4* | 0.38 | -1.40 | 0.43 | -1.21 | 0.41 | -1.30 | 2.25E-22 |
| *IGSF22* | 0.34 | -1.55 | 0.39 | -1.35 | 0.37 | -1.45 | 2.48E-22 |
| *TRIM22* | 2.68 | 1.42 | 2.69 | 1.43 | 2.68 | 1.42 | 2.49E-22 |
| *MACROD2* | 2.82 | 1.49 | 2.74 | 1.45 | 2.78 | 1.47 | 2.54E-22 |
| *ARHGAP18* | 0.43 | -1.21 | 0.40 | -1.34 | 0.41 | -1.27 | 2.75E-22 |
| *KCNJ2* | 3.47 | 1.79 | 3.39 | 1.76 | 3.43 | 1.78 | 2.82E-22 |
| *DNAJC5B* | 2.67 | 1.42 | 2.01 | 1.01 | 2.32 | 1.21 | 3.41E-22 |
| *MIR4750* | 8.25 | 3.04 | 4.49 | 2.17 | 6.08 | 2.60 | 3.88E-22 |
| *BAMBI* | 5.77 | 2.53 | 5.50 | 2.46 | 5.64 | 2.49 | 4.46E-22 |
| *PRG4* | 2.69 | 1.43 | 2.66 | 1.41 | 2.67 | 1.42 | 5.36E-22 |
| *FOS* | 0.31 | -1.69 | 0.33 | -1.61 | 0.32 | -1.65 | 5.78E-22 |
| *CHST13* | 0.12 | -3.06 | 0.17 | -2.59 | 0.14 | -2.82 | 5.99E-22 |
| *AGAP1* | 0.52 | -0.95 | 0.47 | -1.08 | 0.49 | -1.02 | 6.55E-22 |
| *DTX3L* | 3.05 | 1.61 | 2.90 | 1.53 | 2.97 | 1.57 | 6.90E-22 |
| *MIR320E* | 2.70 | 1.43 | 2.10 | 1.07 | 2.38 | 1.25 | 6.94E-22 |
| *APOBEC3A* | 18.80 | 4.23 | 16.24 | 4.02 | 17.48 | 4.13 | 8.05E-22 |
| *KLHL3* | 0.47 | -1.10 | 0.52 | -0.95 | 0.49 | -1.02 | 8.66E-22 |
| *C19orf59* | 0.08 | -3.71 | 0.16 | -2.68 | 0.11 | -3.19 | 8.69E-22 |
| *SRCRB4D* | 0.48 | -1.06 | 0.46 | -1.13 | 0.47 | -1.09 | 9.05E-22 |
| *PARP12* | 3.89 | 1.96 | 3.40 | 1.76 | 3.63 | 1.86 | 9.74E-22 |
| *TESC* | 0.41 | -1.30 | 0.53 | -0.91 | 0.46 | -1.11 | 1.34E-21 |
| *NCRNA00321* | 0.50 | -1.00 | 0.48 | -1.07 | 0.49 | -1.03 | 1.42E-21 |
| *IFIT2* | 10.59 | 3.41 | 10.32 | 3.37 | 10.45 | 3.39 | 1.47E-21 |
| *PARP14* | 2.93 | 1.55 | 2.66 | 1.41 | 2.79 | 1.48 | 1.57E-21 |
| *IGFBP3* | 2.22 | 1.15 | 2.02 | 1.02 | 2.12 | 1.08 | 1.70E-21 |
| *CCDC68* | 0.40 | -1.31 | 0.41 | -1.30 | 0.40 | -1.31 | 1.79E-21 |
| *CXCL10* | 19.91 | 4.32 | 22.12 | 4.47 | 20.98 | 4.39 | 1.86E-21 |
| *SP110* | 2.94 | 1.56 | 2.77 | 1.47 | 2.86 | 1.51 | 1.91E-21 |
| *UPB1* | 3.61 | 1.85 | 2.60 | 1.38 | 3.07 | 1.62 | 2.00E-21 |
| *TRAF1* | 2.23 | 1.15 | 1.91 | 0.93 | 2.06 | 1.04 | 4.04E-21 |
| *RTN4R* | 0.30 | -1.73 | 0.33 | -1.60 | 0.32 | -1.67 | 4.11E-21 |
| *ADM* | 5.73 | 2.52 | 3.22 | 1.69 | 4.29 | 2.10 | 4.12E-21 |
| *DDX58* | 5.80 | 2.54 | 5.41 | 2.43 | 5.60 | 2.49 | 4.58E-21 |
| *FAM70A* | 3.55 | 1.83 | 2.68 | 1.42 | 3.08 | 1.63 | 5.95E-21 |
| *OR52K2* | 7.54 | 2.91 | 5.37 | 2.42 | 6.36 | 2.67 | 6.26E-21 |
| *FMNL2* | 2.89 | 1.53 | 2.76 | 1.46 | 2.82 | 1.50 | 6.67E-21 |
| *LPAR3* | 4.12 | 2.04 | 2.76 | 1.46 | 3.37 | 1.75 | 7.74E-21 |
| *NOV* | 0.23 | -2.11 | 0.27 | -1.87 | 0.25 | -1.99 | 8.41E-21 |
| *SLC39A8* | 2.14 | 1.09 | 3.20 | 1.68 | 2.62 | 1.39 | 9.19E-21 |
| *CDKN1A* | 4.74 | 2.25 | 3.01 | 1.59 | 3.78 | 1.92 | 9.77E-21 |
| *CYP2J2* | 8.23 | 3.04 | 6.69 | 2.74 | 7.42 | 2.89 | 1.20E-20 |
| *HIST1H3D* | 4.04 | 2.02 | 2.68 | 1.42 | 3.29 | 1.72 | 1.33E-20 |
| *SECTM1* | 2.77 | 1.47 | 3.19 | 1.67 | 2.97 | 1.57 | 1.50E-20 |
| *MYBL2* | 4.71 | 2.24 | 4.80 | 2.26 | 4.76 | 2.25 | 1.50E-20 |
| *CXCL13* | 18.21 | 4.19 | 5.94 | 2.57 | 10.40 | 3.38 | 1.51E-20 |
| *HESX1* | 11.83 | 3.56 | 12.38 | 3.63 | 12.10 | 3.60 | 1.75E-20 |
| *C15orf52* | 0.33 | -1.58 | 0.37 | -1.42 | 0.35 | -1.50 | 1.78E-20 |
| *TOR1B* | 2.50 | 1.32 | 2.38 | 1.25 | 2.44 | 1.29 | 1.81E-20 |
| *SAMD9L* | 4.78 | 2.26 | 4.59 | 2.20 | 4.69 | 2.23 | 1.83E-20 |
| *PPBP* | 0.18 | -2.46 | 0.29 | -1.77 | 0.23 | -2.12 | 1.93E-20 |
| *C17orf96* | 3.00 | 1.59 | 2.69 | 1.43 | 2.84 | 1.51 | 2.44E-20 |
| *SCARB1* | 0.35 | -1.53 | 0.33 | -1.58 | 0.34 | -1.55 | 2.66E-20 |
| *CHSY1* | 2.19 | 1.13 | 1.87 | 0.90 | 2.02 | 1.02 | 2.77E-20 |
| *OTOA* | 0.45 | -1.15 | 0.39 | -1.36 | 0.42 | -1.25 | 2.79E-20 |
| *PROC* | 0.32 | -1.63 | 0.43 | -1.23 | 0.37 | -1.43 | 2.89E-20 |
| *HIST1H2AD* | 2.70 | 1.44 | 2.37 | 1.25 | 2.53 | 1.34 | 2.97E-20 |
| *FFAR2* | 13.55 | 3.76 | 6.30 | 2.66 | 9.24 | 3.21 | 3.07E-20 |
| *IFIT5* | 3.61 | 1.85 | 3.30 | 1.72 | 3.46 | 1.79 | 3.40E-20 |
| *P2RY2* | 0.25 | -2.02 | 0.35 | -1.52 | 0.29 | -1.77 | 3.59E-20 |
| *PAPLN* | 2.94 | 1.55 | 2.09 | 1.07 | 2.48 | 1.31 | 4.06E-20 |
| *CEACAM19* | 0.29 | -1.78 | 0.40 | -1.32 | 0.34 | -1.55 | 4.33E-20 |
| *TUBA1C* | 2.22 | 1.15 | 1.84 | 0.88 | 2.02 | 1.02 | 4.34E-20 |
| *LOC401074* | 2.69 | 1.43 | 2.61 | 1.38 | 2.65 | 1.40 | 4.44E-20 |
| *HLX* | 2.21 | 1.15 | 1.83 | 0.88 | 2.01 | 1.01 | 4.74E-20 |
| *ITGA2* | 2.40 | 1.26 | 2.29 | 1.20 | 2.34 | 1.23 | 5.33E-20 |
| *SOBP* | 7.39 | 2.88 | 6.12 | 2.61 | 6.72 | 2.75 | 5.38E-20 |
| *KCNS3* | 2.15 | 1.10 | 2.61 | 1.38 | 2.37 | 1.24 | 5.81E-20 |
| *OSBPL1A* | 2.18 | 1.13 | 2.00 | 1.00 | 2.09 | 1.06 | 5.93E-20 |
| *PRR16* | 5.31 | 2.41 | 2.91 | 1.54 | 3.93 | 1.97 | 6.43E-20 |
| *C10orf55* | 0.47 | -1.09 | 0.44 | -1.19 | 0.45 | -1.14 | 6.70E-20 |
| *PNPLA7* | 0.44 | -1.17 | 0.49 | -1.02 | 0.47 | -1.10 | 7.02E-20 |
| *CCDC144C* | 0.50 | -0.99 | 0.50 | -1.01 | 0.50 | -1.00 | 7.21E-20 |
| *FLJ22447* | 0.42 | -1.25 | 0.57 | -0.80 | 0.49 | -1.02 | 1.03E-19 |
| *PARM1* | 0.36 | -1.48 | 0.47 | -1.10 | 0.41 | -1.29 | 1.18E-19 |
| *SOCS1* | 7.11 | 2.83 | 3.56 | 1.83 | 5.03 | 2.33 | 1.33E-19 |
| *LOC728723* | 0.29 | -1.79 | 0.38 | -1.41 | 0.33 | -1.60 | 1.42E-19 |
| *KIAA1199* | 42.68 | 5.42 | 9.16 | 3.20 | 19.78 | 4.31 | 1.73E-19 |
| *MAPK11* | 2.57 | 1.36 | 2.19 | 1.13 | 2.37 | 1.25 | 1.81E-19 |
| *SYNPO* | 2.47 | 1.31 | 2.08 | 1.05 | 2.27 | 1.18 | 1.93E-19 |
| *PLEKHH1* | 2.97 | 1.57 | 2.29 | 1.20 | 2.61 | 1.38 | 2.09E-19 |
| *FAM153A* | 0.46 | -1.13 | 0.45 | -1.15 | 0.45 | -1.14 | 2.24E-19 |
| *MSC* | 8.36 | 3.06 | 3.18 | 1.67 | 5.16 | 2.37 | 2.31E-19 |
| *PRKCDBP* | 3.77 | 1.91 | 2.95 | 1.56 | 3.34 | 1.74 | 2.34E-19 |
| *CKAP4* | 5.84 | 2.55 | 3.18 | 1.67 | 4.31 | 2.11 | 2.40E-19 |
| *A2M* | 0.43 | -1.23 | 0.35 | -1.50 | 0.39 | -1.37 | 2.85E-19 |
| *MYO7A* | 0.38 | -1.39 | 0.41 | -1.28 | 0.40 | -1.34 | 2.90E-19 |
| *C5orf32* | 2.37 | 1.25 | 1.97 | 0.98 | 2.16 | 1.11 | 2.90E-19 |
| *SCG3* | 2.21 | 1.14 | 2.13 | 1.09 | 2.17 | 1.12 | 3.00E-19 |
| *CES1P1* | 2.22 | 1.15 | 2.40 | 1.26 | 2.31 | 1.21 | 3.58E-19 |
| *TNFSF11* | 0.42 | -1.25 | 0.55 | -0.87 | 0.48 | -1.06 | 3.93E-19 |
| *TRIM14* | 2.11 | 1.08 | 2.06 | 1.04 | 2.09 | 1.06 | 4.85E-19 |
| *PDGFRL* | 14.66 | 3.87 | 19.09 | 4.25 | 16.73 | 4.06 | 5.63E-19 |
| *EPSTI1* | 4.04 | 2.01 | 3.80 | 1.93 | 3.92 | 1.97 | 7.27E-19 |
| *SCO2* | 2.28 | 1.19 | 2.59 | 1.38 | 2.43 | 1.28 | 7.94E-19 |
| *S100A9* | 0.32 | -1.63 | 0.33 | -1.61 | 0.32 | -1.62 | 9.96E-19 |
| *RSAD2* | 10.20 | 3.35 | 9.45 | 3.24 | 9.81 | 3.29 | 1.05E-18 |
| *ISG15* | 7.72 | 2.95 | 7.01 | 2.81 | 7.36 | 2.88 | 1.13E-18 |
| *ANKRD1* | 64.94 | 6.02 | 16.81 | 4.07 | 33.02 | 5.05 | 1.20E-18 |
| *TMEM200A* | 2.74 | 1.46 | 1.99 | 0.99 | 2.34 | 1.22 | 1.43E-18 |
| *LOC147646* | 0.48 | -1.07 | 0.50 | -1.00 | 0.49 | -1.03 | 1.99E-18 |
| *CCDC149* | 0.47 | -1.09 | 0.45 | -1.15 | 0.46 | -1.12 | 2.16E-18 |
| *CNRIP1* | 0.18 | -2.51 | 0.39 | -1.36 | 0.26 | -1.93 | 2.27E-18 |
| *IER5* | 2.41 | 1.27 | 1.73 | 0.79 | 2.04 | 1.03 | 2.35E-18 |
| *MAFF* | 3.27 | 1.71 | 2.07 | 1.05 | 2.60 | 1.38 | 2.40E-18 |
| *GUCY1B3* | 3.25 | 1.70 | 3.21 | 1.68 | 3.23 | 1.69 | 3.76E-18 |
| *LILRA5* | 6.83 | 2.77 | 5.41 | 2.44 | 6.08 | 2.60 | 4.24E-18 |
| *IL2RA* | 4.36 | 2.13 | 3.26 | 1.70 | 3.77 | 1.91 | 4.62E-18 |
| *TLN2* | 0.45 | -1.17 | 0.54 | -0.90 | 0.49 | -1.03 | 4.72E-18 |
| *LIM2* | 0.38 | -1.41 | 0.53 | -0.92 | 0.45 | -1.16 | 5.19E-18 |
| *ADAMTS2* | 5.38 | 2.43 | 3.00 | 1.59 | 4.02 | 2.01 | 5.66E-18 |
| *ENTHD1* | 2.65 | 1.41 | 1.93 | 0.95 | 2.26 | 1.18 | 5.71E-18 |
| *IFI35* | 3.79 | 1.92 | 3.69 | 1.88 | 3.74 | 1.90 | 8.02E-18 |
| *THBD* | 0.27 | -1.86 | 0.41 | -1.30 | 0.33 | -1.58 | 8.59E-18 |
| *USP18* | 9.75 | 3.29 | 8.13 | 3.02 | 8.91 | 3.15 | 9.28E-18 |
| *IFIT1* | 8.69 | 3.12 | 7.54 | 2.91 | 8.09 | 3.02 | 9.53E-18 |
| *PARP9* | 3.58 | 1.84 | 3.32 | 1.73 | 3.45 | 1.79 | 9.55E-18 |
| *DOC2A* | 0.42 | -1.24 | 0.51 | -0.97 | 0.46 | -1.11 | 1.06E-17 |
| *SERPINE1* | 0.18 | -2.48 | 0.18 | -2.50 | 0.18 | -2.49 | 1.08E-17 |
| *EMP1* | 2.74 | 1.45 | 1.96 | 0.97 | 2.32 | 1.21 | 1.18E-17 |
| *ANXA10* | 4.60 | 2.20 | 3.98 | 1.99 | 4.28 | 2.10 | 1.23E-17 |
| *HIST1H2BC* | 6.72 | 2.75 | 3.03 | 1.60 | 4.51 | 2.17 | 1.33E-17 |
| *WNT10A* | 2.42 | 1.27 | 2.22 | 1.15 | 2.31 | 1.21 | 1.34E-17 |
| *IL36RN* | 78.19 | 6.29 | 12.19 | 3.61 | 30.62 | 4.94 | 1.83E-17 |
| *AATK* | 0.43 | -1.21 | 0.42 | -1.24 | 0.43 | -1.22 | 2.03E-17 |
| *TMOD1* | 2.92 | 1.54 | 1.96 | 0.97 | 2.39 | 1.26 | 2.77E-17 |
| *FCER1A* | 0.03 | -5.30 | 0.06 | -4.17 | 0.04 | -4.73 | 2.92E-17 |
| *PLSCR1* | 3.94 | 1.98 | 3.01 | 1.59 | 3.44 | 1.78 | 3.01E-17 |
| *CES1P2* | 2.39 | 1.25 | 2.43 | 1.28 | 2.41 | 1.27 | 3.06E-17 |
| *3-Sep* | 0.40 | -1.31 | 0.46 | -1.11 | 0.43 | -1.21 | 3.16E-17 |
| *NCEH1* | 0.55 | -0.86 | 0.45 | -1.17 | 0.49 | -1.02 | 3.19E-17 |
| *ARNTL2* | 3.34 | 1.74 | 2.25 | 1.17 | 2.74 | 1.45 | 3.42E-17 |
| *STEAP3* | 0.32 | -1.65 | 0.43 | -1.21 | 0.37 | -1.43 | 4.22E-17 |
| *GP1BA* | 0.46 | -1.12 | 0.39 | -1.38 | 0.42 | -1.25 | 4.81E-17 |
| *SIGLEC14* | 2.30 | 1.20 | 1.86 | 0.90 | 2.07 | 1.05 | 5.48E-17 |
| *FERMT2* | 3.40 | 1.76 | 2.23 | 1.16 | 2.75 | 1.46 | 6.04E-17 |
| *IFIT3* | 7.08 | 2.82 | 6.79 | 2.76 | 6.94 | 2.79 | 6.20E-17 |
| *HTR3A* | 0.48 | -1.05 | 0.50 | -1.00 | 0.49 | -1.02 | 6.47E-17 |
| *ZNF366* | 2.74 | 1.45 | 2.13 | 1.09 | 2.41 | 1.27 | 6.55E-17 |
| *DHRS9* | 0.37 | -1.45 | 0.40 | -1.32 | 0.38 | -1.38 | 6.72E-17 |
| *AK8* | 3.89 | 1.96 | 2.38 | 1.25 | 3.04 | 1.60 | 7.23E-17 |
| *QPCT* | 0.32 | -1.62 | 0.34 | -1.56 | 0.33 | -1.59 | 7.37E-17 |
| *MX2* | 4.02 | 2.01 | 3.90 | 1.96 | 3.96 | 1.99 | 7.86E-17 |
| *KCNJ5* | 0.20 | -2.30 | 0.29 | -1.81 | 0.24 | -2.05 | 9.96E-17 |
| *CSF1R* | 0.43 | -1.21 | 0.38 | -1.41 | 0.40 | -1.31 | 1.09E-16 |
| *FAM171B* | 0.43 | -1.23 | 0.58 | -0.78 | 0.50 | -1.01 | 1.28E-16 |
| *LDLRAD3* | 0.43 | -1.22 | 0.49 | -1.03 | 0.46 | -1.12 | 1.55E-16 |
| *HERC6* | 4.97 | 2.31 | 4.33 | 2.11 | 4.64 | 2.21 | 1.59E-16 |
| *LOC283440* | 0.50 | -0.99 | 0.48 | -1.05 | 0.49 | -1.02 | 1.90E-16 |
| *MXD1* | 4.58 | 2.19 | 2.30 | 1.20 | 3.25 | 1.70 | 1.92E-16 |
| *TFRC* | 0.42 | -1.26 | 0.44 | -1.20 | 0.43 | -1.23 | 2.00E-16 |
| *LRP5* | 0.48 | -1.07 | 0.48 | -1.06 | 0.48 | -1.06 | 2.08E-16 |
| *ADORA3* | 0.18 | -2.50 | 0.12 | -3.07 | 0.14 | -2.79 | 2.95E-16 |
| *BATF2* | 5.60 | 2.49 | 5.15 | 2.36 | 5.37 | 2.43 | 3.53E-16 |
| *HMGN2P46* | 4.39 | 2.13 | 2.40 | 1.26 | 3.24 | 1.70 | 3.83E-16 |
| *PNOC* | 2.04 | 1.03 | 2.21 | 1.14 | 2.12 | 1.08 | 3.95E-16 |
| *DDX60L* | 3.90 | 1.96 | 3.47 | 1.80 | 3.68 | 1.88 | 4.03E-16 |
| *PRKAG3* | 0.60 | -0.74 | 0.37 | -1.43 | 0.47 | -1.08 | 4.33E-16 |
| *ACSM5* | 5.64 | 2.49 | 4.02 | 2.01 | 4.76 | 2.25 | 4.34E-16 |
| *NBPF7* | 2.10 | 1.07 | 2.21 | 1.14 | 2.15 | 1.11 | 4.55E-16 |
| *LYN* | 3.04 | 1.60 | 2.14 | 1.10 | 2.55 | 1.35 | 4.77E-16 |
| *ACSL1* | 3.42 | 1.77 | 2.23 | 1.15 | 2.76 | 1.46 | 5.03E-16 |
| *DLL1* | 3.22 | 1.69 | 1.94 | 0.96 | 2.50 | 1.32 | 5.23E-16 |
| *CTGF* | 2.58 | 1.37 | 2.75 | 1.46 | 2.67 | 1.41 | 5.53E-16 |
| *HTR7* | 0.14 | -2.88 | 0.36 | -1.48 | 0.22 | -2.18 | 5.97E-16 |
| *LY6E* | 4.14 | 2.05 | 3.83 | 1.94 | 3.98 | 1.99 | 6.18E-16 |
| *OLIG1* | 4.22 | 2.08 | 2.23 | 1.15 | 3.07 | 1.62 | 6.47E-16 |
| *CDC42EP2* | 8.38 | 3.07 | 3.65 | 1.87 | 5.54 | 2.47 | 6.90E-16 |
| *LILRA2* | 0.36 | -1.46 | 0.46 | -1.13 | 0.41 | -1.30 | 7.00E-16 |
| *CMPK2* | 6.63 | 2.73 | 5.89 | 2.56 | 6.25 | 2.64 | 7.27E-16 |
| *OAS3* | 4.86 | 2.28 | 4.37 | 2.13 | 4.61 | 2.20 | 7.69E-16 |
| *CLCN4* | 0.32 | -1.65 | 0.29 | -1.80 | 0.30 | -1.73 | 8.61E-16 |
| *PRIC285* | 4.80 | 2.26 | 4.39 | 2.13 | 4.59 | 2.20 | 8.83E-16 |
| *HIST2H3D* | 2.94 | 1.56 | 1.96 | 0.97 | 2.40 | 1.26 | 1.25E-15 |
| *C1QC* | 0.40 | -1.33 | 0.55 | -0.87 | 0.47 | -1.10 | 1.32E-15 |
| *KIR2DL4* | 6.01 | 2.59 | 2.95 | 1.56 | 4.21 | 2.07 | 1.55E-15 |
| *BIRC5* | 2.60 | 1.38 | 2.27 | 1.18 | 2.43 | 1.28 | 1.58E-15 |
| *JUP* | 3.05 | 1.61 | 3.45 | 1.79 | 3.25 | 1.70 | 1.58E-15 |
| *DIRC3* | 4.14 | 2.05 | 2.46 | 1.30 | 3.19 | 1.67 | 2.04E-15 |
| *OLIG2* | 22.71 | 4.51 | 6.55 | 2.71 | 12.19 | 3.61 | 2.19E-15 |
| *LTBP2* | 0.29 | -1.78 | 0.24 | -2.06 | 0.26 | -1.92 | 2.25E-15 |
| *TBC1D30* | 0.43 | -1.23 | 0.58 | -0.77 | 0.50 | -1.00 | 2.39E-15 |
| *FAM3B* | 2.68 | 1.42 | 2.41 | 1.27 | 2.54 | 1.34 | 2.49E-15 |
| *ADORA2A* | 2.23 | 1.16 | 1.88 | 0.91 | 2.05 | 1.04 | 3.00E-15 |
| *GPRASP2* | 0.47 | -1.10 | 0.53 | -0.93 | 0.50 | -1.01 | 3.01E-15 |
| *DDX60* | 3.41 | 1.77 | 3.09 | 1.63 | 3.25 | 1.70 | 3.40E-15 |
| *KCNK5* | 2.87 | 1.52 | 2.70 | 1.44 | 2.78 | 1.48 | 3.73E-15 |
| *SLC17A7* | 0.44 | -1.17 | 0.51 | -0.97 | 0.48 | -1.07 | 3.87E-15 |
| *EEF1A2* | 2.38 | 1.25 | 1.94 | 0.96 | 2.15 | 1.10 | 4.05E-15 |
| *LOC100294362* | 2.77 | 1.47 | 2.20 | 1.14 | 2.47 | 1.30 | 5.13E-15 |
| *SLC47A2* | 0.51 | -0.96 | 0.47 | -1.08 | 0.49 | -1.02 | 5.70E-15 |
| *LGALS9* | 2.11 | 1.08 | 2.24 | 1.16 | 2.17 | 1.12 | 5.97E-15 |
| *MX1* | 4.55 | 2.19 | 4.35 | 2.12 | 4.45 | 2.15 | 6.49E-15 |
| *CORO2B* | 1.63 | 0.71 | 2.58 | 1.36 | 2.05 | 1.03 | 6.55E-15 |
| *SPARC* | 0.24 | -2.05 | 0.21 | -2.25 | 0.23 | -2.15 | 6.78E-15 |
| *FAM110C* | 0.47 | -1.09 | 0.51 | -0.98 | 0.49 | -1.04 | 7.04E-15 |
| *DUSP1* | 3.04 | 1.60 | 1.95 | 0.96 | 2.43 | 1.28 | 7.05E-15 |
| *EIF2AK2* | 3.58 | 1.84 | 3.18 | 1.67 | 3.38 | 1.76 | 7.32E-15 |
| *CES1* | 2.31 | 1.21 | 1.87 | 0.90 | 2.08 | 1.05 | 7.58E-15 |
| *HERC5* | 5.32 | 2.41 | 4.81 | 2.27 | 5.06 | 2.34 | 8.56E-15 |
| *LPL* | 0.15 | -2.69 | 0.11 | -3.15 | 0.13 | -2.92 | 1.06E-14 |
| *MAP1A* | 4.79 | 2.26 | 5.11 | 2.35 | 4.95 | 2.31 | 1.35E-14 |
| *PNPT1* | 3.40 | 1.76 | 3.07 | 1.62 | 3.23 | 1.69 | 1.39E-14 |
| *DPYS* | 2.08 | 1.06 | 2.17 | 1.12 | 2.12 | 1.09 | 1.62E-14 |
| *RGMA* | 6.58 | 2.72 | 6.84 | 2.77 | 6.71 | 2.75 | 1.72E-14 |
| *TNFAIP6* | 21.97 | 4.46 | 8.41 | 3.07 | 13.59 | 3.76 | 1.94E-14 |
| *LOC100133669* | 6.03 | 2.59 | 3.31 | 1.73 | 4.47 | 2.16 | 1.97E-14 |
| *PPM1H* | 0.47 | -1.10 | 0.45 | -1.14 | 0.46 | -1.12 | 2.19E-14 |
| *HSD3BP4* | 0.42 | -1.26 | 0.41 | -1.29 | 0.41 | -1.28 | 2.42E-14 |
| *CHRNB2* | 2.72 | 1.44 | 2.26 | 1.17 | 2.48 | 1.31 | 2.54E-14 |
| *ADAMDEC1* | 2.45 | 1.30 | 1.89 | 0.92 | 2.16 | 1.11 | 2.73E-14 |
| *KCNJ10* | 3.23 | 1.69 | 2.84 | 1.51 | 3.03 | 1.60 | 3.15E-14 |
| *LRRC32* | 4.33 | 2.12 | 2.57 | 1.36 | 3.34 | 1.74 | 3.40E-14 |
| *EMR1* | 2.99 | 1.58 | 2.22 | 1.15 | 2.58 | 1.37 | 3.42E-14 |
| *OAS2* | 3.29 | 1.72 | 3.31 | 1.73 | 3.30 | 1.72 | 3.50E-14 |
| *C1QA* | 0.34 | -1.57 | 0.53 | -0.92 | 0.42 | -1.25 | 4.09E-14 |
| *ELOVL7* | 9.53 | 3.25 | 3.61 | 1.85 | 5.86 | 2.55 | 4.35E-14 |
| *CPAMD8* | 0.39 | -1.35 | 0.59 | -0.75 | 0.48 | -1.05 | 4.46E-14 |
| *EPHB1* | 3.83 | 1.94 | 3.47 | 1.79 | 3.64 | 1.87 | 4.65E-14 |
| *FSCN1* | 3.50 | 1.81 | 2.12 | 1.08 | 2.72 | 1.45 | 5.22E-14 |
| *GMPR* | 6.45 | 2.69 | 6.06 | 2.60 | 6.25 | 2.64 | 5.59E-14 |
| *SHOX2* | 3.42 | 1.78 | 2.04 | 1.03 | 2.64 | 1.40 | 5.95E-14 |
| *P2RY13* | 0.12 | -3.02 | 0.08 | -3.64 | 0.10 | -3.33 | 7.11E-14 |
| *SLITRK4* | 0.48 | -1.05 | 0.44 | -1.18 | 0.46 | -1.11 | 8.55E-14 |
| *CH25H* | 15.84 | 3.99 | 7.94 | 2.99 | 11.21 | 3.49 | 1.07E-13 |
| *CCR2* | 0.21 | -2.27 | 0.12 | -3.11 | 0.15 | -2.69 | 1.42E-13 |
| *SDC3* | 2.96 | 1.56 | 3.01 | 1.59 | 2.98 | 1.58 | 1.43E-13 |
| *LAP3* | 2.13 | 1.09 | 2.26 | 1.17 | 2.19 | 1.13 | 1.48E-13 |
| *MMP9* | 0.31 | -1.68 | 0.36 | -1.49 | 0.33 | -1.59 | 1.55E-13 |
| *RBP1* | 0.41 | -1.27 | 0.49 | -1.04 | 0.45 | -1.16 | 1.64E-13 |
| *NMB* | 3.31 | 1.73 | 2.05 | 1.03 | 2.60 | 1.38 | 1.77E-13 |
| *XAF1* | 3.14 | 1.65 | 2.91 | 1.54 | 3.03 | 1.60 | 1.78E-13 |
| *NRXN2* | 3.40 | 1.77 | 2.29 | 1.20 | 2.79 | 1.48 | 1.86E-13 |
| *SLAMF9* | 0.40 | -1.31 | 0.38 | -1.40 | 0.39 | -1.35 | 2.09E-13 |
| *OR52B6* | 3.06 | 1.61 | 2.78 | 1.47 | 2.91 | 1.54 | 2.18E-13 |
| *FZD4* | 3.27 | 1.71 | 2.04 | 1.03 | 2.58 | 1.37 | 2.50E-13 |
| *NCRNA00256A* | 3.29 | 1.72 | 3.16 | 1.66 | 3.22 | 1.69 | 2.50E-13 |
| *GCNT1* | 2.73 | 1.45 | 2.56 | 1.36 | 2.65 | 1.40 | 2.66E-13 |
| *SCAMP5* | 2.46 | 1.30 | 3.59 | 1.85 | 2.97 | 1.57 | 2.75E-13 |
| *MMRN1* | 0.37 | -1.45 | 0.62 | -0.68 | 0.48 | -1.07 | 2.83E-13 |
| *CLLU1OS* | 3.90 | 1.96 | 3.41 | 1.77 | 3.64 | 1.87 | 3.06E-13 |
| *HS3ST3A1* | 2.06 | 1.04 | 2.04 | 1.03 | 2.05 | 1.04 | 3.37E-13 |
| *SLC22A16* | 3.54 | 1.82 | 2.21 | 1.14 | 2.80 | 1.48 | 3.59E-13 |
| *TSPAN7* | 0.46 | -1.11 | 0.48 | -1.06 | 0.47 | -1.08 | 3.67E-13 |
| *HAMP* | 0.33 | -1.59 | 0.38 | -1.41 | 0.35 | -1.50 | 4.41E-13 |
| *SEMA3A* | 0.21 | -2.28 | 0.48 | -1.06 | 0.31 | -1.67 | 4.82E-13 |
| *NOTCH3* | 0.42 | -1.24 | 0.30 | -1.74 | 0.36 | -1.49 | 5.16E-13 |
| *KCNE1L* | 12.42 | 3.63 | 5.40 | 2.43 | 8.19 | 3.03 | 5.44E-13 |
| *LILRB2* | 2.20 | 1.13 | 1.87 | 0.90 | 2.03 | 1.02 | 5.78E-13 |
| *LILRA1* | 0.44 | -1.19 | 0.41 | -1.29 | 0.42 | -1.24 | 6.18E-13 |
| *PTPRS* | 2.56 | 1.36 | 2.75 | 1.46 | 2.65 | 1.41 | 6.28E-13 |
| *CD14* | 0.13 | -2.95 | 0.24 | -2.05 | 0.18 | -2.50 | 6.49E-13 |
| *TCHH* | 8.75 | 3.13 | 3.51 | 1.81 | 5.54 | 2.47 | 6.57E-13 |
| *PID1* | 0.01 | -6.50 | 0.14 | -2.83 | 0.04 | -4.66 | 7.36E-13 |
| *NSUN7* | 1.98 | 0.99 | 3.17 | 1.66 | 2.50 | 1.32 | 7.63E-13 |
| *LEP* | 0.22 | -2.22 | 0.34 | -1.57 | 0.27 | -1.89 | 8.14E-13 |
| *SLC24A3* | 2.38 | 1.25 | 2.74 | 1.46 | 2.56 | 1.35 | 9.00E-13 |
| *PLVAP* | 5.18 | 2.37 | 5.02 | 2.33 | 5.10 | 2.35 | 9.24E-13 |
| *TSPAN13* | 4.57 | 2.19 | 3.96 | 1.98 | 4.25 | 2.09 | 9.29E-13 |
| *NGFR* | 4.77 | 2.25 | 3.28 | 1.71 | 3.95 | 1.98 | 9.92E-13 |
| *IFITM3* | 3.61 | 1.85 | 4.08 | 2.03 | 3.83 | 1.94 | 1.22E-12 |
| *STEAP4* | 0.15 | -2.70 | 0.20 | -2.33 | 0.18 | -2.51 | 1.76E-12 |
| *COL24A1* | 2.22 | 1.15 | 2.16 | 1.11 | 2.19 | 1.13 | 2.10E-12 |
| *RASL10A* | 0.32 | -1.63 | 0.34 | -1.56 | 0.33 | -1.59 | 2.20E-12 |
| *STON2* | 2.68 | 1.42 | 4.16 | 2.06 | 3.34 | 1.74 | 2.27E-12 |
| *GLI3* | 2.54 | 1.34 | 2.20 | 1.14 | 2.36 | 1.24 | 2.31E-12 |
| *COL4A2* | 3.93 | 1.97 | 1.98 | 0.99 | 2.79 | 1.48 | 2.34E-12 |
| *CD300C* | 0.37 | -1.45 | 0.39 | -1.35 | 0.38 | -1.40 | 2.36E-12 |
| *SYPL2* | 1.99 | 0.99 | 2.36 | 1.24 | 2.17 | 1.12 | 2.52E-12 |
| *LGALS2* | 2.58 | 1.37 | 2.36 | 1.24 | 2.47 | 1.30 | 2.58E-12 |
| *MPZL2* | 0.35 | -1.51 | 0.43 | -1.21 | 0.39 | -1.36 | 2.62E-12 |
| *MMP19* | 4.45 | 2.15 | 2.06 | 1.05 | 3.03 | 1.60 | 2.65E-12 |
| *HRH2* | 0.48 | -1.05 | 0.44 | -1.19 | 0.46 | -1.12 | 2.79E-12 |
| *LILRA6* | 2.12 | 1.09 | 1.96 | 0.97 | 2.04 | 1.03 | 2.91E-12 |
| *CCL8* | 19.93 | 4.32 | 13.43 | 3.75 | 16.36 | 4.03 | 2.99E-12 |
| *TFCP2L1* | 0.34 | -1.55 | 0.45 | -1.17 | 0.39 | -1.36 | 4.23E-12 |
| *HRH1* | 2.98 | 1.57 | 2.16 | 1.11 | 2.54 | 1.34 | 5.53E-12 |
| *TNFRSF8* | 0.45 | -1.16 | 0.51 | -0.96 | 0.48 | -1.06 | 6.40E-12 |
| *KIAA1274* | 0.36 | -1.47 | 0.53 | -0.93 | 0.44 | -1.20 | 8.70E-12 |
| *MS4A6A* | 0.31 | -1.70 | 0.28 | -1.83 | 0.29 | -1.76 | 9.00E-12 |
| *ITGB8* | 3.73 | 1.90 | 1.71 | 0.77 | 2.52 | 1.33 | 1.07E-11 |
| *FLJ42418* | 4.54 | 2.18 | 4.97 | 2.31 | 4.75 | 2.25 | 1.18E-11 |
| *SLC22A4* | 2.50 | 1.32 | 1.71 | 0.78 | 2.07 | 1.05 | 1.28E-11 |
| *FCGR2A* | 0.42 | -1.24 | 0.44 | -1.19 | 0.43 | -1.21 | 1.47E-11 |
| *DSCAML1* | 0.45 | -1.14 | 0.48 | -1.06 | 0.47 | -1.10 | 1.50E-11 |
| *AGRN* | 2.52 | 1.33 | 2.51 | 1.33 | 2.52 | 1.33 | 1.72E-11 |
| *IFI44* | 3.60 | 1.85 | 3.34 | 1.74 | 3.47 | 1.80 | 2.13E-11 |
| *CCL3* | 7.19 | 2.85 | 2.95 | 1.56 | 4.61 | 2.20 | 2.48E-11 |
| *PRG2* | 2.51 | 1.33 | 2.15 | 1.10 | 2.32 | 1.22 | 2.73E-11 |
| *LOC731424* | 14.97 | 3.90 | 4.13 | 2.05 | 7.86 | 2.97 | 2.81E-11 |
| *IL3RA* | 3.64 | 1.86 | 1.98 | 0.99 | 2.68 | 1.42 | 3.25E-11 |
| *EXOC3L1* | 3.30 | 1.72 | 3.79 | 1.92 | 3.53 | 1.82 | 3.30E-11 |
| *TTC39A* | 5.08 | 2.34 | 5.38 | 2.43 | 5.23 | 2.39 | 3.39E-11 |
| *MOXD1* | 2.24 | 1.16 | 2.32 | 1.21 | 2.28 | 1.19 | 3.70E-11 |
| *CTNND2* | 6.57 | 2.72 | 3.41 | 1.77 | 4.73 | 2.24 | 4.14E-11 |
| *SLC8A1* | 2.10 | 1.07 | 2.08 | 1.06 | 2.09 | 1.07 | 4.15E-11 |
| *SIGLEC1* | 2.84 | 1.51 | 3.14 | 1.65 | 2.99 | 1.58 | 4.28E-11 |
| *CLEC4C* | 4.73 | 2.24 | 8.05 | 3.01 | 6.17 | 2.62 | 4.43E-11 |
| *ZSWIM4* | 3.43 | 1.78 | 1.80 | 0.84 | 2.48 | 1.31 | 4.86E-11 |
| *DTNA* | 0.22 | -2.21 | 0.43 | -1.23 | 0.30 | -1.72 | 5.48E-11 |
| *E2F7* | 2.10 | 1.07 | 3.76 | 1.91 | 2.81 | 1.49 | 5.53E-11 |
| *FLT3* | 2.61 | 1.38 | 1.76 | 0.81 | 2.14 | 1.10 | 5.70E-11 |
| *MTVR2* | 3.03 | 1.60 | 2.17 | 1.12 | 2.57 | 1.36 | 6.35E-11 |
| *DUSP6* | 2.39 | 1.26 | 1.83 | 0.87 | 2.09 | 1.06 | 6.62E-11 |
| *NRIP3* | 2.82 | 1.50 | 1.61 | 0.69 | 2.14 | 1.09 | 7.10E-11 |
| *DCDC5* | 2.50 | 1.32 | 1.81 | 0.86 | 2.13 | 1.09 | 7.50E-11 |
| *ENPP2* | 3.10 | 1.63 | 4.12 | 2.04 | 3.57 | 1.84 | 8.46E-11 |
| *C20orf195* | 4.25 | 2.09 | 3.33 | 1.74 | 3.76 | 1.91 | 1.01E-10 |
| *SLC9A7P1* | 0.39 | -1.36 | 0.36 | -1.46 | 0.38 | -1.41 | 1.14E-10 |
| *PPM1J* | 2.08 | 1.05 | 2.17 | 1.12 | 2.12 | 1.08 | 1.25E-10 |
| *EGR2* | 3.39 | 1.76 | 2.06 | 1.04 | 2.64 | 1.40 | 1.38E-10 |
| *SASH1* | 2.29 | 1.20 | 1.97 | 0.98 | 2.13 | 1.09 | 1.72E-10 |
| *LOC644242* | 0.34 | -1.54 | 0.40 | -1.31 | 0.37 | -1.42 | 2.17E-10 |
| *CLEC12B* | 2.73 | 1.45 | 2.40 | 1.26 | 2.56 | 1.36 | 2.32E-10 |
| *IFI6* | 3.24 | 1.70 | 3.33 | 1.74 | 3.28 | 1.72 | 2.54E-10 |
| *MYO7B* | 2.62 | 1.39 | 2.10 | 1.07 | 2.34 | 1.23 | 2.54E-10 |
| *CYP27B1* | 6.41 | 2.68 | 3.06 | 1.61 | 4.42 | 2.15 | 2.88E-10 |
| *NIPAL4* | 2.61 | 1.39 | 5.62 | 2.49 | 3.83 | 1.94 | 2.93E-10 |
| *P4HA2* | 2.09 | 1.07 | 1.94 | 0.95 | 2.01 | 1.01 | 3.05E-10 |
| *RAI14* | 0.57 | -0.81 | 0.40 | -1.32 | 0.48 | -1.07 | 3.05E-10 |
| *PAPSS2* | 0.23 | -2.10 | 0.49 | -1.04 | 0.34 | -1.57 | 3.23E-10 |
| *SLC11A1* | 0.13 | -2.89 | 0.36 | -1.49 | 0.22 | -2.19 | 3.36E-10 |
| *PHEX* | 2.15 | 1.10 | 3.48 | 1.80 | 2.73 | 1.45 | 3.64E-10 |
| *IFI44L* | 3.80 | 1.93 | 3.41 | 1.77 | 3.60 | 1.85 | 3.90E-10 |
| *CBR1* | 2.09 | 1.06 | 2.10 | 1.07 | 2.09 | 1.07 | 4.24E-10 |
| *LOC144571* | 0.45 | -1.14 | 0.32 | -1.66 | 0.38 | -1.40 | 5.82E-10 |
| *TGFA* | 2.53 | 1.34 | 1.62 | 0.70 | 2.02 | 1.02 | 6.16E-10 |
| *LPAR1* | 2.30 | 1.20 | 2.41 | 1.27 | 2.35 | 1.23 | 6.47E-10 |
| *CCL7* | 19.95 | 4.32 | 6.02 | 2.59 | 10.96 | 3.45 | 7.85E-10 |
| *CLDN14* | 3.87 | 1.95 | 1.80 | 0.85 | 2.64 | 1.40 | 8.37E-10 |
| *DYSF* | 2.26 | 1.17 | 2.31 | 1.21 | 2.28 | 1.19 | 1.17E-09 |
| *CCDC80* | 3.14 | 1.65 | 2.20 | 1.14 | 2.63 | 1.40 | 1.36E-09 |
| *KCNE1* | 3.18 | 1.67 | 2.09 | 1.06 | 2.58 | 1.37 | 1.72E-09 |
| *MT2A* | 4.17 | 2.06 | 2.56 | 1.36 | 3.27 | 1.71 | 1.90E-09 |
| *ARHGAP20* | 0.34 | -1.57 | 0.48 | -1.06 | 0.40 | -1.32 | 1.93E-09 |
| *MMP7* | 0.37 | -1.44 | 0.52 | -0.95 | 0.44 | -1.20 | 2.34E-09 |
| *MEIS3* | 3.70 | 1.89 | 2.11 | 1.07 | 2.79 | 1.48 | 2.54E-09 |
| *FPR1* | 0.31 | -1.70 | 0.18 | -2.45 | 0.24 | -2.08 | 2.79E-09 |
| *NCRNA00189* | 3.74 | 1.90 | 2.01 | 1.01 | 2.74 | 1.46 | 3.02E-09 |
| *FCGR2C* | 0.35 | -1.50 | 0.26 | -1.97 | 0.30 | -1.73 | 3.27E-09 |
| *SLC12A3* | 2.72 | 1.44 | 2.89 | 1.53 | 2.81 | 1.49 | 3.89E-09 |
| *LILRA4* | 2.36 | 1.24 | 3.41 | 1.77 | 2.84 | 1.50 | 4.14E-09 |
| *AK4* | 6.65 | 2.73 | 1.98 | 0.98 | 3.63 | 1.86 | 4.45E-09 |
| *CCR10* | 2.27 | 1.18 | 2.15 | 1.11 | 2.21 | 1.14 | 4.48E-09 |
| *SPECC1* | 0.43 | -1.21 | 0.57 | -0.80 | 0.50 | -1.01 | 5.12E-09 |
| *CHI3L1* | 0.30 | -1.74 | 0.25 | -2.01 | 0.27 | -1.88 | 5.83E-09 |
| *LIMCH1* | 2.55 | 1.35 | 1.84 | 0.88 | 2.17 | 1.12 | 6.36E-09 |
| *C20orf103* | 2.56 | 1.36 | 3.42 | 1.77 | 2.96 | 1.56 | 6.76E-09 |
| *DLEU7* | 0.49 | -1.02 | 0.35 | -1.49 | 0.42 | -1.26 | 1.00E-08 |
| *IFNG* | 4.84 | 2.27 | 2.64 | 1.40 | 3.57 | 1.84 | 1.32E-08 |
| *PANX2* | 5.37 | 2.42 | 2.00 | 1.00 | 3.27 | 1.71 | 1.44E-08 |
| *C15orf48* | 4.46 | 2.16 | 1.98 | 0.99 | 2.97 | 1.57 | 1.44E-08 |
| *HSPA7* | 0.36 | -1.46 | 0.29 | -1.81 | 0.32 | -1.64 | 1.60E-08 |
| *PLEKHD1* | 3.30 | 1.72 | 4.54 | 2.18 | 3.87 | 1.95 | 2.61E-08 |
| *KAL1* | 0.43 | -1.21 | 0.56 | -0.85 | 0.49 | -1.03 | 3.74E-08 |
| *CUX2* | 1.99 | 0.99 | 2.49 | 1.32 | 2.22 | 1.15 | 4.02E-08 |
| *ALK* | 0.36 | -1.48 | 0.43 | -1.23 | 0.39 | -1.35 | 4.03E-08 |
| *OSBPL6* | 2.70 | 1.44 | 2.95 | 1.56 | 2.82 | 1.50 | 4.16E-08 |
| *CDH1* | 2.83 | 1.50 | 2.06 | 1.04 | 2.41 | 1.27 | 4.91E-08 |
| *CCL2* | 3.78 | 1.92 | 1.81 | 0.85 | 2.61 | 1.39 | 5.08E-08 |
| *CYP4F11* | 2.51 | 1.33 | 1.76 | 0.81 | 2.10 | 1.07 | 7.81E-08 |
| *LOC285758* | 3.25 | 1.70 | 1.78 | 0.83 | 2.41 | 1.27 | 8.79E-08 |
| *CYP1A1* | 4.60 | 2.20 | 1.65 | 0.72 | 2.75 | 1.46 | 9.00E-08 |
| *GPBAR1* | 2.38 | 1.25 | 2.24 | 1.16 | 2.31 | 1.21 | 9.07E-08 |
| *EPHA2* | 5.02 | 2.33 | 7.54 | 2.92 | 6.15 | 2.62 | 9.14E-08 |
| *PTGDS* | 2.49 | 1.32 | 3.26 | 1.71 | 2.85 | 1.51 | 1.12E-07 |
| *ELFN1* | 3.30 | 1.72 | 1.69 | 0.76 | 2.36 | 1.24 | 1.14E-07 |
| *INHBA* | 14.74 | 3.88 | 3.42 | 1.77 | 7.09 | 2.83 | 1.33E-07 |
| *PRUNE2* | 2.02 | 1.02 | 2.13 | 1.09 | 2.07 | 1.05 | 1.47E-07 |
| *ATP6V0D2* | 0.49 | -1.03 | 0.43 | -1.21 | 0.46 | -1.12 | 1.49E-07 |
| *BMP2* | 0.41 | -1.28 | 0.60 | -0.74 | 0.50 | -1.01 | 2.09E-07 |
| *HCAR3* | 3.14 | 1.65 | 2.21 | 1.15 | 2.64 | 1.40 | 2.16E-07 |
| *CEACAM3* | 0.28 | -1.82 | 0.48 | -1.07 | 0.37 | -1.44 | 2.26E-07 |
| *GJB2* | 3.87 | 1.95 | 2.46 | 1.30 | 3.09 | 1.63 | 2.42E-07 |
| *CLEC4F* | 3.73 | 1.90 | 3.51 | 1.81 | 3.62 | 1.86 | 3.55E-07 |
| *ODZ4* | 0.37 | -1.43 | 0.52 | -0.93 | 0.44 | -1.18 | 4.08E-07 |
| *CD93* | 0.06 | -3.99 | 0.41 | -1.28 | 0.16 | -2.63 | 4.51E-07 |
| *HPSE* | 3.25 | 1.70 | 1.84 | 0.88 | 2.45 | 1.29 | 4.67E-07 |
| *PCSK6* | 0.35 | -1.50 | 0.63 | -0.66 | 0.47 | -1.08 | 5.54E-07 |
| *C1orf226* | 2.87 | 1.52 | 2.35 | 1.23 | 2.59 | 1.38 | 5.69E-07 |
| *LILRA3* | 6.36 | 2.67 | 2.46 | 1.30 | 3.96 | 1.98 | 6.26E-07 |
| *FFAR3* | 6.68 | 2.74 | 2.16 | 1.11 | 3.79 | 1.92 | 9.59E-07 |
| *IL6* | 3.10 | 1.63 | 1.53 | 0.61 | 2.16 | 1.11 | 9.69E-07 |
| *HSD11B1* | 0.13 | -2.97 | 0.37 | -1.42 | 0.22 | -2.19 | 1.18E-06 |
| *CRISPLD2* | 0.56 | -0.85 | 0.28 | -1.83 | 0.40 | -1.34 | 1.39E-06 |
| *DNASE1L3* | 3.55 | 1.83 | 6.12 | 2.61 | 4.66 | 2.22 | 1.78E-06 |
| *CASP5* | 3.17 | 1.66 | 1.98 | 0.99 | 2.50 | 1.32 | 4.01E-06 |
| *KRT5* | 4.61 | 2.21 | 4.45 | 2.15 | 4.53 | 2.18 | 4.04E-06 |
| *ADAMTS14* | 3.25 | 1.70 | 2.70 | 1.43 | 2.96 | 1.57 | 5.02E-06 |
| *FN1* | 0.38 | -1.41 | 0.28 | -1.83 | 0.33 | -1.62 | 5.40E-06 |
| *HCAR2* | 4.17 | 2.06 | 2.30 | 1.20 | 3.10 | 1.63 | 5.80E-06 |
| *VNN1* | 0.04 | -4.56 | 0.34 | -1.54 | 0.12 | -3.04 | 5.93E-06 |
| *CYP7B1* | 2.67 | 1.42 | 1.54 | 0.62 | 2.02 | 1.02 | 6.78E-06 |
| *SLC35F3* | 2.46 | 1.30 | 2.04 | 1.03 | 2.24 | 1.16 | 8.30E-06 |
| *IL1R1* | 0.27 | -1.87 | 0.58 | -0.78 | 0.40 | -1.32 | 8.43E-06 |
| *SIGLEC12* | 0.45 | -1.16 | 0.39 | -1.37 | 0.42 | -1.26 | 9.31E-06 |
| *TM7SF4* | 3.47 | 1.80 | 1.77 | 0.82 | 2.48 | 1.31 | 1.08E-05 |
| *MT1E* | 2.59 | 1.37 | 2.55 | 1.35 | 2.57 | 1.36 | 1.27E-05 |
| *DLL4* | 2.84 | 1.51 | 1.45 | 0.54 | 2.03 | 1.02 | 3.42E-05 |
| *LAMB3* | 0.28 | -1.81 | 0.68 | -0.56 | 0.44 | -1.19 | 3.55E-05 |
| *GPRC5B* | 0.36 | -1.46 | 0.41 | -1.27 | 0.39 | -1.36 | 3.61E-05 |
| *CD207* | 0.33 | -1.60 | 0.59 | -0.77 | 0.44 | -1.18 | 5.41E-05 |
| *CSTA* | 0.31 | -1.70 | 0.65 | -0.63 | 0.45 | -1.17 | 5.67E-05 |
| *GPR124* | 0.26 | -1.93 | 0.70 | -0.51 | 0.43 | -1.22 | 6.41E-05 |
| *ANGPT1* | 0.51 | -0.96 | 0.47 | -1.09 | 0.49 | -1.02 | 0.0001 |
| *PROCR* | 3.33 | 1.73 | 1.34 | 0.42 | 2.11 | 1.08 | 0.0002 |
| *OSM* | 3.53 | 1.82 | 1.52 | 0.61 | 2.31 | 1.21 | 0.0002 |
| *C6orf223* | 3.81 | 1.93 | 1.39 | 0.48 | 2.30 | 1.20 | 0.0003 |
| *MAMLD1* | 3.49 | 1.80 | 1.55 | 0.64 | 2.33 | 1.22 | 0.0003 |
| *WNT5A* | 4.20 | 2.07 | 1.66 | 0.73 | 2.64 | 1.40 | 0.0003 |
| *CSF1* | 2.86 | 1.51 | 1.55 | 0.63 | 2.10 | 1.07 | 0.0004 |
| *LIF* | 5.23 | 2.39 | 1.37 | 0.45 | 2.67 | 1.42 | 0.0004 |
| *LILRB5* | 0.42 | -1.25 | 0.44 | -1.18 | 0.43 | -1.22 | 0.0004 |
| *C1QTNF1* | 4.60 | 2.20 | 1.29 | 0.37 | 2.43 | 1.28 | 0.0004 |
| *CCL18* | 1.38 | 0.47 | 3.80 | 1.93 | 2.29 | 1.20 | 0.0005 |
| *CXCL6* | 0.13 | -2.92 | 0.42 | -1.26 | 0.24 | -2.08 | 0.0007 |
| *FCGR1A* | 0.47 | -1.09 | 0.49 | -1.02 | 0.48 | -1.06 | 0.0009 |
| *MT1M* | 2.57 | 1.36 | 3.36 | 1.75 | 2.94 | 1.55 | 0.0009 |
| *STAC* | 0.41 | -1.28 | 0.36 | -1.49 | 0.38 | -1.39 | 0.0009 |
| *TUBB3* | 3.42 | 1.77 | 1.19 | 0.24 | 2.01 | 1.01 | 0.0011 |
| *CXCL9* | 2.91 | 1.54 | 6.87 | 2.78 | 4.47 | 2.16 | 0.0012 |
| *DSP* | 2.36 | 1.24 | 6.44 | 2.69 | 3.89 | 1.96 | 0.0016 |
| *IL1A* | 0.28 | -1.83 | 0.55 | -0.87 | 0.39 | -1.35 | 0.0023 |
| *CXCL5* | 0.14 | -2.79 | 0.53 | -0.93 | 0.28 | -1.86 | 0.0030 |
| *SIGLEC15* | 0.14 | -2.87 | 0.83 | -0.27 | 0.34 | -1.57 | 0.0115 |
| *CXCL1* | 0.36 | -1.48 | 0.69 | -0.53 | 0.50 | -1.00 | 0.0329 |

^a^Fold change gene expression for the virus-stimulated PBMCs (low responders) relative to the unstimulated PBMCs (low responders} ^b^Log_2_ of the fold change, described in (a). ^c^Fold change gene expression for the virus-stimulated PBMCs (high responders) relative to the unstimulated PBMCs (high responders}. ^d^Log_2_ of the fold change, described in (c). ^e^Fold change gene expression for the virus-stimulated PBMCs (all subjects) relative to the unstimulated PBMCs (all subjects) ^f^Log_2_ of the fold change, described in (e).^g^P-value associated with the test for a differential gene expression in all subjects (response to viral stimulation).
