# Supplementary material for: Whole Transcriptome Profiling Identifies CD93 and Other Plasma Cell Survival Factor Genes Associated with Measles-Specific Antibody Response after Vaccination
Source: PLoS One. 2016 Aug 16;11(8):e0160970. doi: 10.1371/journal.pone.0160970 (PMC4987012; doi:10.1371/journal.pone.0160970)
Supplement: S3 Table — (DOCX) [file pone.0160970.s003.docx]

**S3 Table.** Significant genesets, in high vs low antibody vaccine responders (response to viral stimulation)

| **Geneset Description** | **PMID Number** | **p-value** |
| --- | --- | --- |
| SHETH_LIVER_CANCER_VS_TXNIP_LOSS_PAM2 | 16607285 | 0.001 |
| GSE14000_4H_VS_16H_LPS_DC_TRANSLATED_RNA_UP | 19943945 | 0.001 |
| GSE15750_WT_VS_TRAF6KO_DAY6_EFF_CD8_TCELL_DN | 19494812 | 0.001 |
| CELL_CELL_ADHESION |  | 0.002 |
| GSE360_CTRL_VS_B_MALAYI_LOW_DOSE_DC_DN | 12663451 | 0.002 |
| GSE30962_PRIM_VS_SECON_CHRONIC_LCMV_INF_CD8_TCELL_DN | 21856186 | 0.002 |
| GSE6269_HEALTHY_VS_STREP_PNEUMO_INF_PBMC_DN | 17105821 | 0.002 |

The description is the geneset name, as identified in the MSigDB (Subramanian A. *et al*., Gene set enrichment analysis: a knowledge-based approach for interpreting genome-wide expression profiles. Proc NatlAcadSci US A 2005; 102:15545). For more information, the table also provides the PubMed identification numbers for published papers associated with each of the statistically significant genesets.
